# Deep Sequencing of the Murine Olfactory Receptor Neuron Transcriptome

**DOI:** 10.1371/journal.pone.0113170

**Published:** 2015-01-15

**Authors:** Ninthujah Kanageswaran, Marilen Demond, Maximilian Nagel, Benjamin S. P. Schreiner, Sabrina Baumgart, Paul Scholz, Janine Altmüller, Christian Becker, Julia F. Doerner, Heike Conrad, Sonja Oberland, Christian H. Wetzel, Eva M. Neuhaus, Hanns Hatt, Günter Gisselmann

**Affiliations:** 1 Ruhr-University Bochum, Department of Cell Physiology, Bochum, Germany; 2 University Duisburg-Essen, Institute of Medical Radiation Biology, Essen, Germany; 3 University of Köln, Cologne Center for Genomics, Köln, Germany; 4 Cluster of Excellence and DFG Research Center Nanoscale Microscopy and Molecular Physiology of the Brain (CNMPB), Göttingen, Germany; 5 Pharmacology and Toxicology, University Hospital Jena, Drackendorfer Str. 1, 07747 Jena, Germany; 6 Cluster of Excellence NeuroCure, Charité-Universitätsmedizin Berlin, Charitéplatz 1, 10117 Berlin, Germany; 7 University of Regensburg, Department of Psychiatry and Psychotherapy, Molecular Neurosciences, Regensburg, Germany; Monell Chemical Senses Center, UNITED STATES

## Abstract

The ability of animals to sense and differentiate among thousands of odorants relies on a large set of olfactory receptors (OR) and a multitude of accessory proteins within the olfactory epithelium (OE). ORs and related signaling mechanisms have been the subject of intensive studies over the past years, but our knowledge regarding olfactory processing remains limited. The recent development of next generation sequencing (NGS) techniques encouraged us to assess the transcriptome of the murine OE. We analyzed RNA from OEs of female and male adult mice and from fluorescence-activated cell sorting (FACS)-sorted olfactory receptor neurons (ORNs) obtained from transgenic OMP-GFP mice. The Illumina RNA-Seq protocol was utilized to generate up to 86 million reads per transcriptome. In OE samples, nearly all OR and trace amine-associated receptor (TAAR) genes involved in the perception of volatile amines were detectably expressed. Other genes known to participate in olfactory signaling pathways were among the 200 genes with the highest expression levels in the OE. To identify OE-specific genes, we compared olfactory neuron expression profiles with RNA-Seq transcriptome data from different murine tissues. By analyzing different transcript classes, we detected the expression of non-olfactory GPCRs in ORNs and established an expression ranking for GPCRs detected in the OE. We also identified other previously undescribed membrane proteins as potential new players in olfaction. The quantitative and comprehensive transcriptome data provide a virtually complete catalogue of genes expressed in the OE and present a useful tool to uncover candidate genes involved in, for example, olfactory signaling, OR trafficking and recycling, and proliferation.

## Introduction

The sense of smell has been perfected to detect a tremendous range of different volatile chemical substances. Olfactory receptors (OR) constitute the largest superfamily of mammalian G protein-coupled receptors (GPCRs) and account for the vast discriminatory power of the olfactory system. In humans, this discriminatory power is achieved by the relatively small number of approximately 400 functional ORs, whereas mice possess a considerably larger number of OR genes with approximately 900 functional ORs [1–6]. Despite their lower number of functional genes, humans can discriminate more than 10,000 different odors. Presumably, this discrimination is achieved by a combinatorial code, in which single receptor types are able to respond to several different odorant molecules, and single odorant compounds are recognized by a number of different receptor types [7]. According to the classical olfactory signal transduction scheme, receptor-ligand interactions in the ciliary membranes of the olfactory receptor neurons (ORNs) lead to activation of olfactory-specific G-proteins (Gα_olf_) [8], stimulation of adenylyl cyclase type III (ACIII), production of cyclic adenosin monophosphate (cAMP) [9], and activation of cyclic nucleotide-gated (CNG) channels that are composed of CNGA2, CNGA4 and CNGB1b [10–12]. The initial depolarization produced by the CNG channels is enhanced by subsequent activation of the calcium activated chloride channel (CaCC) ANO2 [13–18]. Focused research on this model of olfactory perception has provided more detail, and the participation of many more components has been reported. The action of the Na^+/^K^+/^Cl^-^-cotransporter Nkcc1, most likely together with Slc4a1 [17] and other transporters, provides the high intracellular chloride concentration necessary for signal amplification [19, 20]. The trafficking of ORs is accomplished by receptor transporting proteins (RTP) [21]. After OR activation, the signal termination and internalization processes are achieved through G protein-coupled receptor kinase (GRK) [22–24]and possibly β-arrestins [25–27]. ORs and accessory proteins bind to the scaffolding multi PDZ-domain proteins (MUPP) to form an assembled transduction complex [28]. The efficacy of the signal transduction cascade is enhanced by the nucleotide exchange factor Ric8b [29–31]. In contrast, signal adaptation is achieved through reduced ACIII activity mediated by calmodulin (CaM) kinases [32], degradation of cAMP by specific phosphodiesterases (PDEs) [33–37], and desensitization of CNG-channels by binding of CaM [38–41].

Stephan et al. (2012) showed that the principal Na^+^/Ca^2+^ exchanger Nckx4 is involved in rapid response termination and proper adaptation [42]. However, adaptation processes are complex, and there are many more proteins involved in the regulation of this mechanism.

Further, an inhibitory signal transduction cascade that involves phosphoinositide 3-kinase (PI3K) has been proposed [43–47]. Although the basic mechanisms involved in signal processing are known, many issues require further exploration before understanding of the complex olfactory perception process on molecular level will be achieved. Hence, it is desirable to identify the plenary repertoire of ORN-specific proteins to fully uncover the factors involved in olfactory signaling pathways.

In recent publications [48, 49], the transcriptome of the murine OE was characterized by microarray analysis. According to this DNA-array study, more than 10,000 genes are expressed in ORNs. ORN-specific genes were identified by the differential analysis of OMP-positive versus OMP-negative neurons using a transgenic OMP-GFP mouse [50], and the specific expression of 300 genes in ORNs was verified by *in situ* hybridization (ISH). Most recently, next generation sequencing (NGS)-based OE transcriptome data focused on sex-specific differences in OR genes were generated for BALB/c mice [51], and the study by Keydar et al. (2013) [52] generated a more general catalogue of genes based on NGS from data of C57BL/6J mice.

Additionally, transcriptome analyses are complemented by proteomic studies of OE plasma and ciliary membranes [53–56, 16, 15] that have led to the detection of approximately 2,346 proteins. Nevertheless, the detection of OR gene expression, which is fundamental for an olfactory tissue, was incomplete because membrane proteins are difficult to detect in such proteome studies, and in some of these studies, ORs failed to be detected at all.

Here, we present the first NGS based transcriptome of fluorescence-activated cell sorting- (FACS)-sorted ORNs in combination with a comprehensive transcriptome study of the murine OE. Our data provide a comprehensive list of transcripts for membrane proteins that included established and previously described proteins and new, previously unrecognized proteins. We present a detailed expression ranking for GPCRs and additionally analyze the expression patterns of several newly identified membrane proteins in the OE.

Compared with microarray [48, 49] and proteome data [53–56, 16, 15] from the OE, deep sequencing of murine the OE transcriptome detects more expressed genes and nearly all known ORs and allows better quantification of expression levels, as verified by quantitative PCR.

Our data provide an important new and sensitive tool to guide novel approaches and advance research on olfactory signaling mechanisms in the OE and, especially, ORNs.

## Materials and Methods

### Animals

CD1 and C57BL/6J mice were obtained from Charles River (Sulzfeld, Germany) and transgenic OMP-GFP mice [50] were kindly provided by Dr. Peter Mombaerts, Max Planck Institute of Biophysics, Frankfurt.

Mice were offered normal laboratory chow and water ad libitum in standard cages. All animal experiments were carried out in accordance with the European Union Community Council guidelines and approved by the competent state office of the Federal Land of Northrhine Westphalia (file number 87–51.04.2010.A180).

### Preparation of mice OE and FACS of ORNs

OE of 4 weeks old male and female CD1 or C57BL/6J mice was prepared and subsequently RNA was isolated; each RNA sample (male and female CD1, female C57BL/6J) was prepared from an OE pool from eight different mice for CD1 mice; in case of C57BL/6J three mice were used for OE preparation. ORNs were obtained from homo- or heterozygous OMP-GFP mice [50]. OE was prepared and collected in 300 µl cold Ringer’s solution. Epithelia were minced and ORNs dissociated by adding papain followed by an incubation of 15 minutes at 37°C. Reaction was stopped by washing with Ringer’s solution; cell suspension was centrifuged 10 minutes at 121 g and cell pellet was resuspended in phosphate buffered saline. Enrichment of ORNs from cell suspension was done by FACS. Altogether, six (homozygous) or eight (heterozygous) adult mice were used to obtain about 100,000 sorted, fluorescent ORNs.

### RNA isolation

Total RNA was isolated either out of the pooled OE or of the sorted ORN sample with the *RNeasy Mini Kit* (Qiagen, Hilden, Germany) according to the manufacturer’s protocol including the optional on-column DNaseI digestion.

### NGS library preparation and Illumina sequencing

Libraries for NGS sequencing were prepared from total RNA and subjected to DSN normalization by standard Illumina protocols. Afterwards, Illumina sequencing was performed on a Genome Analyzer IIx using single end 36-bp (CD1 OE male and female) respectively 75-bp (ORNs homozygous) reads. RNA for heterozygous ORNs was sequenced on a HiSeq-2000 by standard Illumina protocols (101-bp, paired end). In case of C57BL/6J mice and additional probes of CD1 OE, mRNA was sequenced on HiSeq-2000 (101-bp, paired end).

### Alignment of RNA-Seq reads using TopHat

We analyzed the raw sequence data in fastq format as previously described [57]. RNA-Seq reads were aligned to version mm9 of mouse reference genome and transcriptome using TopHat (v2.0.7) [58] which utilizes the ultra-fast short–read mapping program Bowtie to arrange the alignment [59]. TopHat output files in BAM format were sorted and indexed with SAMtools [60]. In order to reduce the alignment of repetitive reads a multiread-correction was used allowing up to 5 hits per read.

### Gene expression calculation using Cufflinks

Aligned RNA-Seq reads for each sample were assembled into transcripts and their abundance was estimated by the program Cufflinks (v1.3.3) [61] using the RefSeq mm9 reference transcriptome in Gene Transfer Format (GTF) obtained from the UCSC Genome Bioinformatics database (University of California Santa Cruz). In order to estimate transcript expression, the GTF-file was supplied to Cufflinks. The parameter—compatible-hits-norm was set to ensure that FPKM normalization was performed based on reference transcriptome only.

Cufflinks was provided with a multifasta file (mm9.fa) to improve accuracy of the relatively transcript abundance estimation [62]. We further used a masked command –M and the mask GTF rmsk.gtf to mask all possible reads from RNA repeats (including tRNA, snRNA, scRNA, srpRNA) short and long interspersed nuclear elements (SINE, LINE) and other classes of repeats.

Cufflinks indicates and quantifies the relative abundances of transcripts in the unit FPKM [61]. For comparison to olfactory tissue, we reanalyzed already published raw RNA-Seq data from brain, liver, muscle and testes [63, 64] in the same manner as our own data. The data sets were visualized and investigated by the Integrative Genomic Viewer (www.broadinstitue.org/igv) for proving sequence alignements and correct mapping of reads for the top expressed genes. While the raw data analysis was performed on a Linux based computer further calculations were carried out with Microsoft Excel 2010. In this study, our intention was to monitor the expression of protein coding genes only. Therefore, we removed all entries for non-polyadenylated transcripts from our analyzed data including microRNA (miRNA) and small nucleolar RNA (snoRNAs). This also improves the comparability to other data sets such as mRNA-Seq versus total-RNA-Seq. DSN-normalization and different types of RNA preparation lead to differences especially in such small RNA species.

For a differential gene expression analysis, we used the program Cuffdiff, which identifies significant changes in transcript expression between two datasets [57].

**Availability of raw data sets.** The raw RNA-Seq data sets (FASTQ file format) for FACS-sorted ORNs and OE were deposited in Gene Expression Omnibus (GEO) repository (www.ncbi.nlm.nih.gov/geo/) under the following accession number: GSE53793.

### Quantitative PCR

Mice were decapitated and OE was collected from septum und turbinates. Total RNA from single mouse samples was isolated with RNeasy Mini Kit (Qiagen, Hilden, Germany) including optional DNaseI digestion and cDNA was prepared using iScript cDNA Synthesis Kit (Bio-Rad, München, Germany). PCR reactions (1 min, 95°C; 1 min, 63°C; 1 min, 72°C; 35 cycles) were performed on an iQ5 thermal cycler using iQ SYBR Green Supermix (both Bio-Rad, München, Germany). At least three independent runs with three technical replicates each were performed and expression levels were calculated using the ddCt method.

Since OR genes contain no introns in their coding sequence, we used genomic DNA as efficiency control and normalized OR cDNA data to OR genomic DNA data.

Primers for housekeeping gene α1b-tubulin were used as described [65], others were designed with Primer-BLAST (sequence 5’ to 3’):

a1b-tubulin long rv (AGCAATGGCTGTGGTGTTGCTCA)
ACIII (ACGACCACAAGCGCTTTCAGG; ACTTGGAAGGCACCAGGGGCA)
mOR-EG short (TGACAGGTTTGTGGCCATCCGC; CAGTATCAAGGAGCATACCACCCCC)
mOR-EG long (TCTCCTCTCTCCTTTCACTTTCACGC; AGGCTTTGCGGCGTCCACTT)
Olfr2 (GGAGCGAAGGAACCACACTGGG; TCAGCACCAACACGTAGGCCA)
Olfr31 (TGCTCGTCTCACATGACTGTGGT; CCCAGTGCCCTCCTCAGACCT)
Olfr169 (TCCTTTTCCACCTGTTCCTTCCACA; TGCCAAAAATTTATCCTGCCCTGGA)
Olfr259 (TCAGAACACAAGTCTACGAGGCTGT; TGCCACATAGCGATCATAGGCCA)
Olfr309 (CTGACTTTCTGTGGGCCGAA; CTCCCACAGCCAGGGTGATA)
Olfr314 (ACCCCCATGCTGAACCCCCT; GGTCCAAGCCAGCCAAGGCA)
Olfr355 (TCGGCCTGAGGACCAAAAACCA; AGGGTCAGAACGGATGGCCAGA)
Olfr411 (CATGCGGCTGGGGTCAGTGG; ATGCCAAGTCTCGCTTTCCTGTGA)
Olfr525 (AGCTGCTGCTGCTCACTGCC; TGCACTGATGACCCACACACTGC)
Olfr533 (GTGACCCCATCTCTGAACCC; GCTTTTAGAGTTCATCTCCAAGCTC)
Olfr545 (GGCTCGCACTGCCTCCCAA; TCAGGGGCCTGCACTCAGGA)
Olfr632 (GGGCCCCGTGTGGCATTGAT; GCGGGTGTCAGAACAGGCCA)
Olfr705 (CCTGGCACTGACACTGGGTGG; TGTGGCCACCATAAGCCAGCA)
Olfr1126 (TGTGGTGAGCTGTGCCACACAA; AGGCTGCTGCCAGCTGAGTG)
Olfr1301 (TGCAACTGCCTCTCTGTGGTCAT; GCTCTGGCGAACAGTGAGAAGGA)
Olfr1347 (TTGACCAACATGACCAGAGTCCAAC; TCTCCAGGAGGGTCAGCAGGT)
Olfr1348 (ATGGACGGGGCCCTACCACA; TGTAGTGGGCGGCAGATGGC)
Olfr1349 (CACCAGCCTGGACTTCGGCC; GGCTGCAGCAGGGTCACCAG)
Olfr1395 (TCCTGGTGGCCCTCTCTGGC; GGAGCCACGGCCCAGGAGAA)
Olfr1507 (GGGCCATGTGGACAGCAGGG; AGGCCAGTTCGATCACCTGAGGT)
Olfr1508 (TGGCTTGCACTGACACCCACA; ACCACGGTGAGATGGGCTGC)
Adipor1 (CGGCAGACAAGAGCAGGAGTGT; AGAACCAGCCCATCTGGCCCA)
Gpr108 (TGAGTCCCGTGAGGAGGGTGC; TGCAGATGCCGAATGGACCAGA)
Gpr177 (AAAGGGGGTCGCAGAAATGGCTG; GGGTGCTGGAGCGATCAAGCCT)
Stomatin (CAGCAACCCGTCTTTTGGCAC; TCCCCCAGTCATCCGTGGCAT)
Stoml2 (TGGACTGCCCCGAAACACCG; CCGGATTCGGTCTAACACGGGG)
Stoml3 (GCCACGTTCCTGCTGGCTCAG; ATTCCCCAGAGCTCGGTGGCA)
RTP1 (GTACCTGGAACTTCATGCCTCAGGC; CACGCATGCGCACCGAACCAG)
RTP2 (GCAGTACCTGGAGCAACATGCCT; CGCATGCGCACTGAGCCAACA)
RTP3 (CGTTTTGAAGCCAGGATGGACGC; GGCCTTCTTCTCACACCACCGC)
RTP4 (GCACCACCTGGCTCGAACCC; GCATCACCCAGTCTGCCACAGT)
REEP1 (ATGGTGCTCCTGCTCCCTCGG; AGGGTTCTAGGCGGTGCCCG)
REEP2 (AACAGCGGCTCCTGCTGCCT; GGCTGGGTACAGGGTGCCAA)
REEP3 (CGCTGAGGCGATCCCAGAGCA; TCTTGGGAAGGGCAAGGTGTAGACA)
REEP4 (CTGAGTGAGCGTCCCTCCGC; ACGGCCTTGTAGGAAGCATACGC)
Tmem30a (CATCGTCGCTACGTGAAATCTCGA; CGCCACATGGCGCAATTGGT)
Tmem30b (GTTCCGCTCCGACGGTCTGC; GATCGAGCGGCACCTCGACG)
Tmem30c (TTTGCAGGAAGTTCGAAGCCTCTGC; AAAGGCAGCCGTCCGCATCC)
Tmem205 (GAGCTGCCACGGTTCTTGGTCC; CCAGGCACCAGACAAGACCAGC)
Tmem16b (GACGCCCAGATGCTGTGAGGA; AGGCGGGGGATAAAGTCGGAGG)

### *In situ* hybridization

Digoxigenin-labeled antisense riboprobes, typically about 600–800 nucleotide length, were generated from cDNA of interest by *in vitro* transcription performed with the DIG RNA labeling mix (Roche, Palo Alto, CA) according to the manufacturer’s instructions. Coronal Cross sections of OE were obtained from wild type C57BL/6J mice of both sexes aged postnatal 21 days (P21). Mice were sacrificed using CO_2_ followed by decapitation. Subsequently the maxillary and anterior cranial region of the head was dissected free. The whole head was fixed in 4% paraformaldehyde in PBS at 4°C overnight, followed by a 24 hours decalcification step with 0.5 M ethylen-diamin-tetra-acetat (EDTA) (pH 8.0) and a final cryoprotecting incubation step with an increasing 10%–30% sucrose in PBS series (10% and 20% sucrose for 1 hour; 30% sucrose for at least 3 hours) at 4°C. Afterwards, coronal sections (12 µm) of quickly frozen heads embedded in tissue freezing medium OCT for supporting tissue during cryotomy (Leica Microsystems, Bensheim, Germany) were cut on a cryostat (Leica, CM 3050S) and mounted on Superfrost Plus Slides (Thermo Scientific, Menzel Gläser). After dehydration using an increasing ethanol series, slices were stored at -80°C until further use.

*In situ* hybridizations were performed as described with minor modifications [66]. Briefly, fixed cryosections were incubated in RIPA-buffer, followed by an acetylation step with acetic anhydride in TEA buffer. Next, a prehybridization step in 50% deionized formamide, 10% dextran sulfate, 5x Denhardt’s solution, 5x SSC, 10 mM DTT, 250 µg/ml yeast tRNA, 500 µg/ml sheared and denatured herring sperm, 50 µg/ml heparin, 2.5 mM EDTA, and 0.1% (v/v) Tween-20 was carried out for 1 h at 55°C to prevent unspecific binding of riboprobes. After each incubation step a wash step with SSC- or PBS^—^/T-buffers followed.

Finally, 50 ng antisense riboprobes were hybridized at 55–65°C on cyrosections mounted on slides overnight. The hybridized mRNA was visualized using an alkaline phosphatase-conjugated antibody to digoxigenin and hydrolysis of nitro-blue tetrazolium chloride/5-bromo-4-chloro-3-indolylphosphate p-toluidine. Antisense and a control sense probe were tested in parallel. The slides were covered with cover slips using polyvinyl-alcohol containing embedding medium (Mowiol, Immo-Mount). Digital images were obtained with Axiocam camera on an Axioscope2 microscope (Zeiss, Oberkochen, Germany). All images of sense and antisense samples were recorded under same conditions (brightness, contrast and light exposure time).

A signal was considered a positive when the antisense labeling was visually noticeably darker than the sense labeling.

### Immunohistochemistry

Immunostainings of the OE were performed on 12 µm coronal cryosections of paraformaldehyde-fixed tissue of OMP-GFP mice [50] on Superfrost slides (Thermo Scientific, Menzel Gläser). After blocking with 1% gelatin or 10% goat serum in phosphate-buffered saline / 0.1% Triton X-100, sections were incubated with primary antibody (TRPC1: 5 µg/ml and TRPM7: 2 µg/ml; kindly provided by D.E. Clapham) and fluorescence-labeled secondary antibody (dilutions 1:500 or 1:1000) in phosphate-buffered saline / 1% gelatin / 0.1% Triton X-100. Stained sections were mounted in ProLong Antifade Gold medium (Molecular Probes). All fluorescence images were collected on a confocal laser scanning microscope (LSM510 Meta, Zeiss, Oberkochen, Germany). ImageJ (NIH) was used for image processing.

### Literature search

In order to identify novel genes in terms of OE, an intense Pubmed (http://www.ncbi.nlm.nih.gov/pubmed) literature search was required. Thereby, the query contained the name of gene interested in combined with the terms “olfactory” or “olfaction”.

## Results

To assess gene expression, the Illumina RNA-Seq protocol was used to amplify and sequence up to 57 million fragments from OEs of male and female CD1 adult mice. Furthermore, 13 million reads were generated for FACS-sorted ORNs obtained from homozygous and 58 million reads from heterozygous transgenic OMP-GFP mice [50] with a C57BL/6 background (Table 1, S1 Table). These OMP-GFP mice are determined to be valid models for assessing ORN gene expression patterns in a study using microarrays [48, 49].

**Table 1:** Summary of RNA-Seq data.

**Tissue/Cells**	**Mouse**			**mRNA-Seq**	**Total number of**		
	**strain**	**genotype**	**Gender**		**Sequenced reads**	**Mapped reads**	**[%]**
Sorted ORNs	C57BL6 transgene OMP-GFP	homozygous	mixed	Illumina/Genome Analyser II, 72 bp reads, single-end	13,574,176	8,857,058	65.25
Sorted ORNs	C57BL6 transgene OMP-GFP	heterozygous	mixed	Illumina/HiSeq2000, 101 bp reads, paired end	58,234,129	34,519,988	59.28
OE1	CD1	WT	male	RNA-Seq (DNS-Normalization) Illumina/Genome Analyser II, 36 bp reads, single-end	37,284,951	28,881,184	77.46
OE2	CD1	WT	male	Illumina/Genome Analyser II, 101 bp reads, paired-end	40,053,764	28,363,505	70.81
OE3	CD1	WT	male	Illumina/Genome Analyser II, 101 bp reads, paired-end	36,141,244	26,397,003	73.04
OE4	CD1	WT	male	Illumina/Genome Analyser II, 101 bp reads, paired-end	11,394,752	8,384,689	73.58
OE1	CD1	WT	female	RNA-Seq (DNS-Normalization)Illumina/Genome Analyser II, 36 bp reads, single-end	52,716,231	38,700,603	73.41
OE2	CD1	WT	female	Illumina/HiSeq 2000, 101 bp reads, paired-end	54,589,506	36,103,429	66.14
OE3	CD1	WT	female	Illumina/HiSeq 2000, 101 bp reads, paired-end	57,808,346	39,268,107	67.93
OE4	CD1	WT	female	Illumina/HiSeq 2000, 101 bp reads, paired-end	33,354,414	24,884,683	74.61
OE5	CD1	WT	female	Illumina/HiSeq 2000, 101 bp reads, paired-end	19,807,752	14,513,434	73.27
OE1	C57BL6	WT	female	Illumina/HiSeq 2000, 101 bp reads, paired-end	86,404,176	63,862,406	73.91
OE2	C57BL6	WT	female	Illumina/HiSeq 2000, 101 bp reads, paired-end	30,763,538	22,694,013	73.77
OE3	C57BL6	WT	female	Illumina/HiSeq 2000, 101 bp reads, paired-end	25,520,854	17,789,702	69,7
OE4	C57BL6	WT	female	Illumina/HiSeq 2000, 101 bp reads, paired-end	25,520,328	18,831,564	73.79

Additionally, we sequenced approximately up to 86 million 101 nt length fragments from the OEs of female C57BL/6 mice (Table 1, S1 Table).

We analyzed our sequencing results using TopHat and Cufflinks software [57], and reads were mapped onto the mouse reference genome mm9. Expression values were calculated from the number of reads per kilobase per million reads in each sample. The quantitative measurement of gene expression employed in the RNA-Seq experiments was the FPKM (fragments per kilobase of exon per million fragments mapped) value.

Our analysis enabled us to detect the expression of 13,955 protein-coding genes in male and 13,280 in female OEs and up to 13,103 genes in FACS-sorted ORNs (FPKM > 1) of the 21,550 genes interrogated with a Refseq-based gene model. Including genes with an extremely low expression level (FPKM < 1), we counted a total of up to 16,786 genes (S1 Table).

To quantify olfactory gene expression relative to other tissues, we reanalyzed the raw sequencing data of previously published murine RNA-Seq NGS transcriptome studies that focused on the brain, muscle, liver and testes [63, 64] in the same manner with which we analyzed our own sequencing data (S3 Table, S4 Table). To ensure the comparability of datasets, we studied expression patterns of different housekeeping genes. A general, rough scale regards FPKM values ~1 to indicate weakly expressed genes, ~10 indicates medium expression, and ~100 indicates highly expressed genes based on comparisons to housekeeping genes (S1 Fig).

## Olfactory Receptor Genes

**Figure 1:**
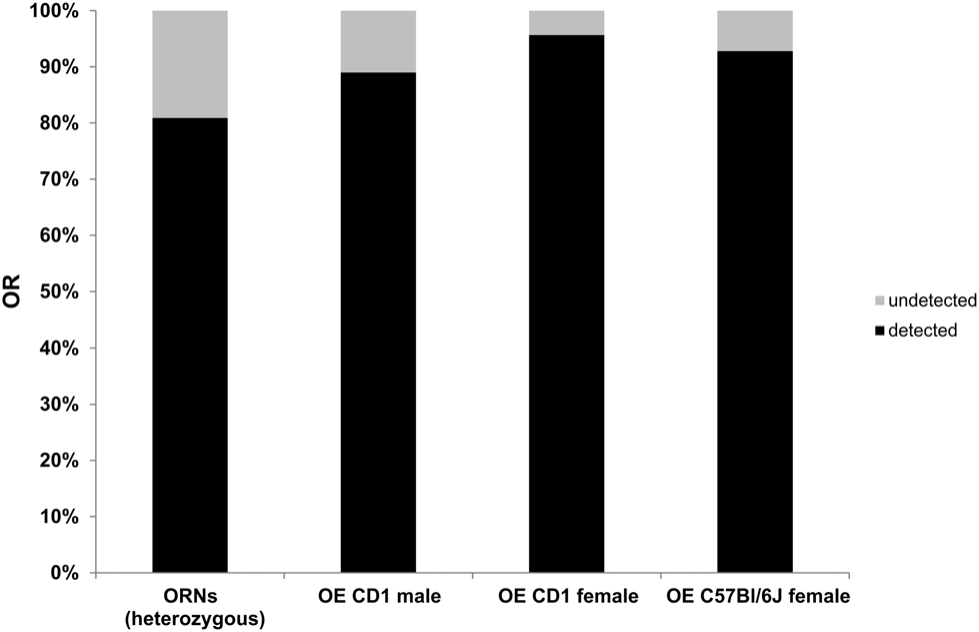
OR detection using RNA-Seq: Bar chart showing the percentage of detected OR genes in female and male OE tissue and FACS-sorted ORNs. Percentages were calculated based on the 1,125 OR genes annotated in the Refseq based gene model.

To analyze the transcriptome in more detail, we first focused on chemoreceptor gene families starting with OR genes. The high sensitivity of RNA-Seq was demonstrated by the detection of nearly all ORs. Of 1,125 annotated OR genes, we detected 1,075 in female and 1,001 in male CD1 mice and 1,044 in female C57BL/6J mice with FPKMs > 0.1 (Fig. 1, S5 Table, S2 Fig.).

Regarding FACS-sorted ORNs, we detected the expression of 582 (homozygous) and 905 (heterozygous) OR genes with FPKM > 0.5. For a typical 1 kb OR coding sequence at a FPKM of 0.1, the expression was confirmed by 3 reads in the OE, and due to the lower sequencing depths for FACS-sorted ORNs at a FPKM of 0.5 (S3 Fig.). As Shiao et al. (2012), we set a similar threshold for the detection of ORs [51].

If very weakly expressed OR genes, whose expression was only supported by 1–2 fragments, were included, expression of an additional ~2% of ORs was detected in both male and female OEs. A total of 98 and 18 ORs had no detectable expression in male and female OEs, respectively.

In comparison, RNA-Seq results from mouse brains, livers and muscles revealed greatly reduced numbers of detected OR genes (3—10 ORs). In contrast, pronounced expression of 155 ORs was detected in the testes (S4 Fig.).

The strength of OR gene expression is strongly dependent on the receptor gene and reached up to 97 FPKM for Olfr1507 in the ORNs. However, most OR genes (up to 73%) were expressed at low levels (FPKMs < 3) due to their mosaic-like expression patterns (S5 Fig.) in the OE. The median expression was 2 in the OE and 2.7 FPKM in the FACS-sorted ORNs.

**Figure 2:**
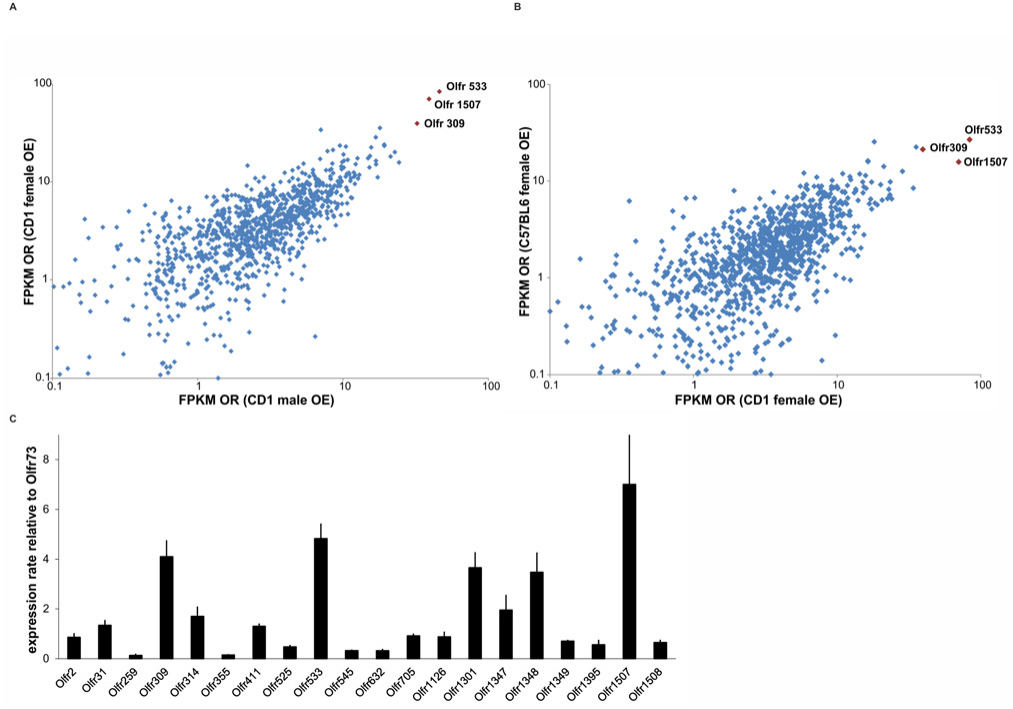
Correlations of expression levels plotted for each detected OR gene. **A.** Correlation of the OR gene expression patterns between male and female CD1 mice. Only OR genes with detectable expression levels (FPKM>0.1) are shown. The FPKM values are logarithmically presented. The Pearson correlation coefficient of r = 0.83 confirmed the strong correlation of OR gene expression patterns between female and male CD1 mice. The three ORs (Olfr533, Olfr1507 and Olfr309) with the highest expression levels were also the most highly expressed in the RNA-Seq data from both sexes. **B.** Correlation of OR gene expression patterns between females of strain CD1 and C57BL6. The Pearson correlation coefficient of r = 0.75 confirmed the strong correlation between the expression patterns of OR genes between the different strains; however, these patterns exhibited greater divergence between strains than between the sexes of the CD1 strain. The most highly expressed ORs, Olfr533 and Olfr309, had the same expression ranking, and Olfr1507 was among the ten most highly expressed OR genes in both strains. **C.** Verification of RNA-Seq results for ORs by real time RT-PCR. Expression levels are relative to mOREG (Olfr73). Error bars represent the SEM.

The expression levels of individual ORs was strongly conserved between the male and female OEs (Pearson correlation coefficient, r = 0.83), and the three most highly expressed receptors (Olfr533, Olfr1507 and Olfr309) were identical in the males and females. Furthermore, the three most highly expressed ORs in the sorted ORNs were found in the top ten most highly expressed genes in the male and female OEs (Fig. 2).

These finding suggest that the expression levels of specific receptor types are not only conserved between sexes but also between different mouse strains, as OEs from CD1 mice and sorted ORNs from transgenic OMP-GFP mice with a C57BL/6J background were examined.

Furthermore, we detected the expression of 21 out of 24 annotated OR pseudo-genes in the OE. Among these pseudo-genes, Olfr1372-ps exhibited the highest expression level (S6 Table). Because previous studies have established that certain OR pseudo-genes are functional [67], Olfr1372-ps is an interesting candidate for future studies.

We additionally verified the RNA-Seq results for several highly expressed OR genes with real time RT-PCR. The expression levels were comparable to that of mOR-EG (Olfr73), a well-characterized mouse OR [68].

We detected similar expression rankings for the 20 tested ORs compared to our RNA-Seq data and confirmed that Olfr1507, Olfr533 and Olfr309 belong to the highly expressed OR genes (Fig. 2).

## Correlation Analysis

### Whole data set

We generated for each condition (OE from CD1 male, CD1 female and C57BL16) up to five biological replicates (Table 1, S1 Table). Correlation measurements for protein-coding gene expression patterns between biological replicates showed that Pearson correlation coefficient values ranged for CD1 male from r = 0.43 to 0.96 (r_mean_ = 0.6), CD1 female from r = 0.35 to 0.91(r_mean_ = 0.67) and C57BL/6 female from r = 0.37 to 0.91 (r_mean_ = 0.61) (S6 Fig.).

Analyzing genes with the most diverging expression pattern between the biological replicates, interestingly, we found predominantly transcripts for odorant-binding proteins (OBPs) (Obp, Mup, Lcn and several other gene families) [51] (S1 Table, S7 Fig.). OBPs function as soluble transport proteins of the nasal mucus [69].

In addition, the expression pattern of several cytochrome P450 (Cyp) genes, which are known to be expressed in the olfactory mucosa [70], showed a high variability in expression level between the OE samples (S1 Table). The presence of these enzymes in the olfactory organ is probably necessary for the metabolic transformation of odorant molecules and they play a protective role by detoxifying inhaled chemicals [70]. It is therefore plausible that mice individually adapt and regulate their olfactory transport system to their appropriate surrounding olfactory environment.

In summary, the genes with the highest variability of expression between individual mice belong to proteins with a major olfactory function. After excluding these genes from the correlation analysis, the biological replicates strongly correlate up to r = 0.97. Even data between CD1 and C57BL6 strains show now a strong correlation (Pearson correlation coefficient values up to r = 0.95) (S6 Fig.). Therefore, these results bode for the reproducibility of our data and represent a good basis for comparative analysis.

### OR subgenome

To ensure reproducibility of the conserved pattern for OR gene expression, we analyzed the additionally generated replicates for each condition (OE of CD1 male, CD1 and C57BL6 female) (S8 Fig., S9 Fig.).

By comparing the highly expressed ORs in each replicate, our analysis confirms that the ORs Olfr533, Olfr1507 and Olfr309 indeed belong to the top expressed OR genes in all OE samples (S10 Fig.).

Further, the expression levels for individual ORs within the biological replicates were strongly conserved and reached Pearson correlation coefficient values up to r = 0.95) (S9 Fig.). OR expression levels were comparable between female CD1 and C57BL/6J mice; the Pearson correlation coefficient were r = 0.65 to 0.86 between replicates (S9 Fig.)

The variance of OR gene expression pattern between replicates within one strain was lower than between strains (S9 Fig.). This shows that the OR gene expression is widely conserved in the OE, whereby the expression pattern of a small set of OR genes is strain specific.

## Other Chemoreceptors

**Figure 3:**
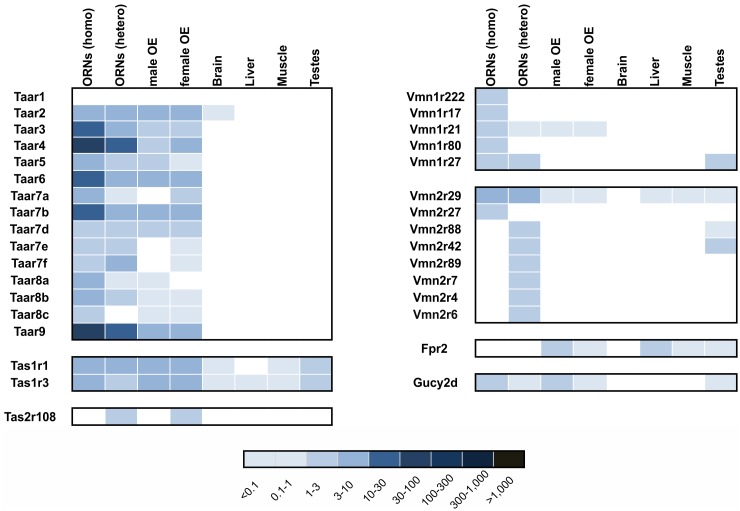
Expression pattern of chemoreceptor genes. Heatmap showing the expression levels of the following chemoreceptor classes: TAARs, VNO receptors, GC-D, taste receptors and FPR in olfactory (male and female OE, and FACS-sorted ORNs) and non-olfactory tissue (brain, muscle, liver and testes). Higher FPKM values are indicated by deeper colors. Only genes with a FPKMs >1 are represented in this chart.

Next, we analyzed other classes of chemoreceptor genes. Our analysis emphasized the specific expression of the “olfactory” trace amine-associated receptors (TAARs) in the OE [71]. All TAARs, with the exception of TAAR1, known to be specifically expressed in the brain, were exclusively detected in OE and FACS-sorted ORNs (Fig. 3). We also detected several vomeronasal organ (VNO) receptors (V1R and V2R) in the OE. Out of the 207 annotated V1R receptors, we observed weak expression of 15 genes with FPKMs of 0.1–0.5 in the OE, and 24 genes with FPKM values of up to 2 in sorted ORNs. Regarding V2R receptors, of the 127 annotated V2Rs we detected 5 genes with low expression levels (0.1–0.7 FPKM) in the OE and up to 21 genes with FPKM values from 0.5 to 8 in ORNs (Fig. 3). Analyses of these expression patterns in C57BL/6J mice yielded basically the same results.

In contrast, the family of formylpeptide receptors (FPR), which function as chemoreceptors in the VNO [72, 73], were largely undetected. Only weak expression of FPR1 and FPR2 genes was noticed (Fig. 3). Likewise, guanylyl cyclase GC-D receptor [74] expression was low (FPKM 0.8) (Fig. 3).

Interestingly, we also detected the expression of taste receptors in the OE. Our data from sorted ORNs revealed an enrichment of Tas1r1 and Tas1r3, (FPKM 6.7), these receptors typically form heterodimers and function as the umami receptor [75]. Type 2 bitter taste receptors (Tas2r) were weakly detected in OE at a FPKM of 0.7 for Tas2r108 (Fig. 3).

## Genes with Known OE-Specific Expression

Next, we analyzed whether our RNA-Seq results matched the reported expression patterns of molecules linked to olfactory signaling cascades. Due to the extreme mosaic-like pattern of OR gene expression, FPKM values for OR expression cannot be directly compared to the values for other genes. Therefore, we calculated the accumulated gene expression of all ORs and assumed that this represented the expression level of a single OR in a single ORN.

**Figure 4:**
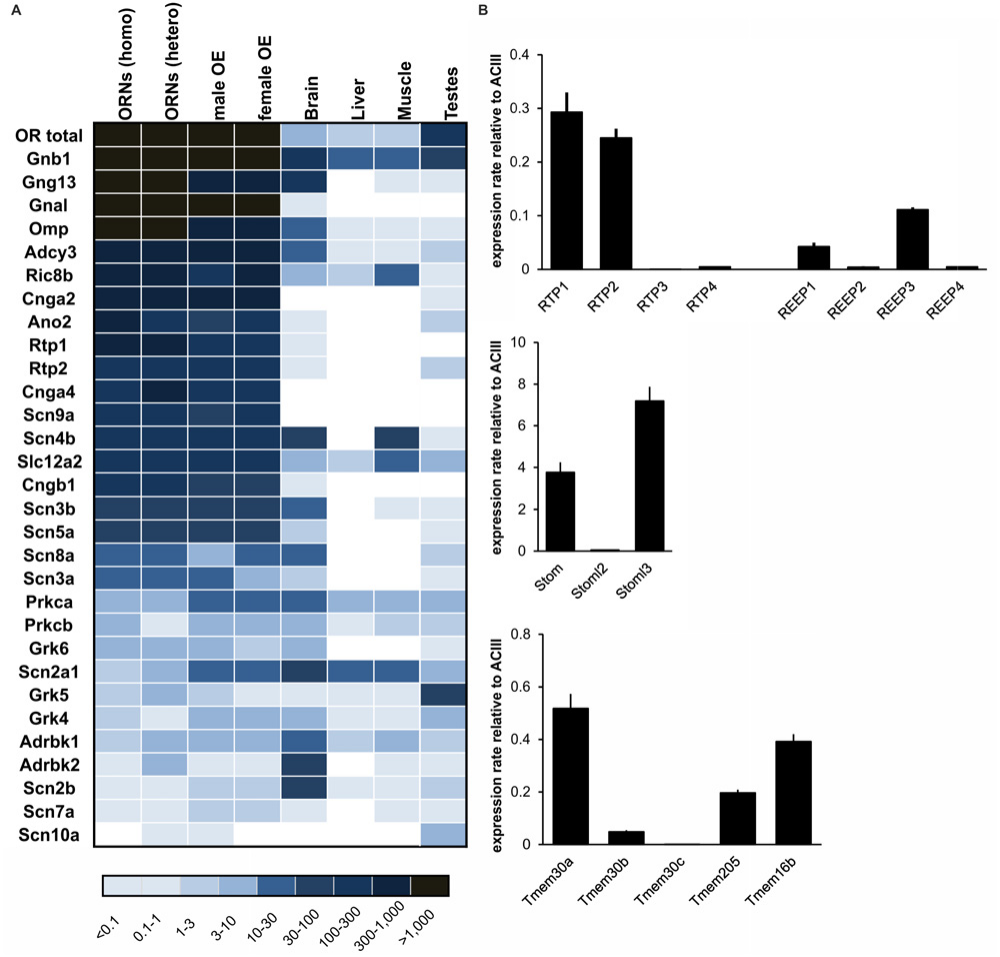
Expression pattern of known genes of the olfactory signal transduction. **A.** Heatmap showing the expression levels of genes known to be involved in olfactory signaling and other genes known to be highly expressed in ORNs as determined by NGS. The FPKM of OR total represents the accumulated gene expression of all ORs and shows that the OR is the most highly expressed gene in the OE. The main components of the signal transduction scheme were among the 200 most highly expressed genes. The FPKM values shown for OMP in sorted ORNs (homozygous) are rough estimations based on the calculation of reads located in the 3’-untranslated regions of OMP and are therefore only valid to a limited extent. Higher FPKM values are indicated by deeper colors. **B.** RT-PCR verification of the highly expressed genes in the OE. Gene expression was normalized to the level of adenylyl cyclase type III (ACIII) RNA. The Investigated genes were RTP1–4, REEP1–4, Stom, Stoml2–3 and transmembrane proteins, including ANO2 (Tmem16b). Error bars represent the SEM.

Under these assumptions, the FPKM value was approximately on average 4,000 (n = 13) which would make the ORs by far the most highly expressed protein coding genes. The other most highly expressed genes (Fig. 4) included OMP, which is one of the most abundant proteins in the OE, followed by the subunits of trimeric G-protein composed of Gα_olf_, Gβ_1_ and Gγ_13,_ and, with a somewhat lower expression rate, Go and Gß_2_. Moreover, the guanine nucleotide exchange factor Ric8b and the receptor interacting proteins RTP1, RTP2 and Reep1 were also among the most highly expressed proteins (Fig. 4). Other classical signaling molecules enriched in ORNs include the following: ACIII (which is the only adenylyl cyclase that we found to be highly enriched in the ORNs), the CNG channel (formed by the CNGA2, -A4 and -B1 subunits [76]), and the calcium-activated chloride channel Ano2 [16, 77]. We also detected ßArr2, which is responsible for OR internalization [78, 27], and a weak expression of βArr1. Among the 1,000 most highly expressed genes that have been implicated in olfactory signaling were Nkcc1 (also called Slc12a2) [14, 19], 6 PKC genes (α, β, δ, ε, η, and ζ), of which only α and β are known to be expressed in mouse ORNs, 5 GRKs (Grk2, Grk3, Grk4, Grk5, Grk6) [79–81, 22–25], and voltage-gated sodium channels (S1 Table) of which Scn9a was the primary and most highly enriched isoform in ORNs[82].

The expression patterns determined by NGS were in good agreement with previous reports about ORN gene expression and confirmed that the key proteins in olfactory signal transductions are strongly expressed in ORNs.

We also verified the RNA-Seq results for several highly expressed genes by real time RT-PCR using ACIII as a standard (Fig. 4). As expected, RTP genes showed the highest expression levels for RTP1 and RTP2. Stomatin-like protein 3 (Stoml3) is known to be expressed in ORNs [83] and showed the highest expression level of the stomatin family genes tested. The expression levels of the tested genes correlated with the FPKM values determined by RNA-Seq.

The top expressed genes in FACS-sorted ORNs from homo- and heterozygous OMP-GFP mice have similar expression levels. Therefore, we assume that a lack of OMP expression has no general influence on the expression of other genes. We calculate a Pearson coefficient of r = 0.9 for gene expression patterns between these two groups, which suggests a strong correlation of gene expression pattern. Additionally, using Cuffdiff analysis, we identified only few statistical significant changes in gene expression between both datasets. Our analysis revealed that only 13 genes were differentially expressed (S2 Table). Therefore, we assume that the data of FACS-sorted homozygous ORNs represent a nearly normal transcriptome of ORNs. These results are in accordance with the study of Sammeta et al. (2007)[48], in which no statistically significant differences in mRNA abundance between these two genotypes were detected by microarray technique. However, the limited sequencing depth of our homozygous ORNs (~13 million) complicates the detection especially of lower differentially expressed genes with a statistical significance, so that more regulated genes will be probably detected at a higher sequencing depth. The OMP expression level in FACS-sorted ORNs with a heterozygous genotype was comparable to the level in the OE datasets.

## Differences in Gene Expression Patterns between OE and ORNS

**Figure 5:**
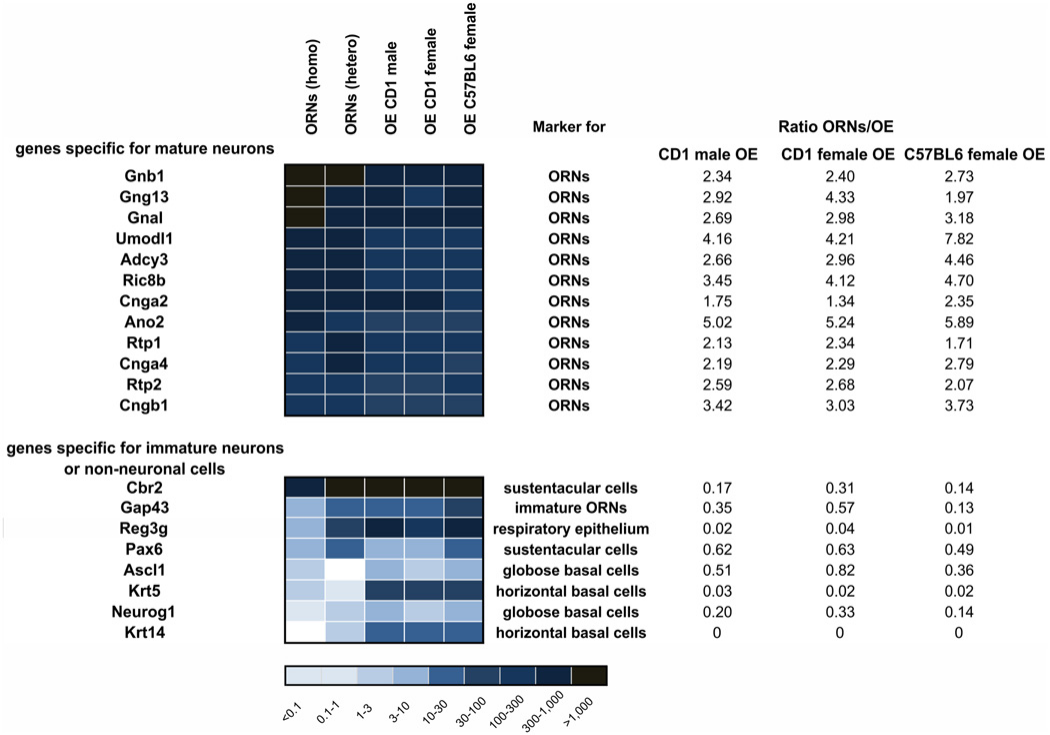
Differences in the gene expression patterns between ORNs and the OE. Comparison of FACS-sorted ORNs and the OE (CD1 OE both sexes; C57BL6 female OE) revealed that genes that are known to be expressed in mature ORNs were expressed in ORNs at levels that were about two to three-fold higher those of the OE. Genes specific for non-neuronal cell types were expressed at levels that were at least two to 119-fold greater in the OE.

The OE is composed of several different cell types. Next to the ORNs are sustentacular cells, basal cells, including globose and horizontal cells, microvillar cells, and cells lining the Bowman’s glands and duct are found in the OE [84, 85]. Accordingly, the differential expression pattern of the OE compared to FACS-sorted ORNs revealed a catalog of genes expressed in ORNs and/or other cell types of the OE. To avoid strain specific differences, we only compared the ORN data with C57BL/6J transcriptome data. We found that, in nearly all instances, genes known to be expressed in ORNs had 2–8 times higher FPKM values in sorted ORNs than in the OE, while genes known to be expressed in other cell types of the OE had 2–119-fold higher FPKMs in the OE compared to the sorted ORNs (Fig. 5).

To examine the expression patterns of non-neuronal cell types, we analyzed the expression of the marker genes reported by Sammeta et al. (2007) [48]. FPKMs of the sustentacular cell markers Cbr2 and Pax6 were higher in the OE but were also detected in ORNs [66], which suggests a small proportion of these cells were present in our FACS-sorted ORN probe. Expression of Krt14 and Krt5, markers of horizontal basal cells, was either weak or absent in the FACS-sorted ORN probe, and stronger expression of these markers was detected in the OE.

Reg3g, a marker of respiratory epithelium, was strongly expressed in the OE and expressed at lower levels in the ORNs. Moreover, Ascl1 and Neurog1, markers for transiently amplifying progenitor cells, were found in the OE and only weakly detected in the ORN probe set. Further, GAP43, a marker of immature neurons, was present in the OE and, to a lesser extent, in the ORN probe.

These expression pattern analyses confirm the purity of the FACS-sorted ORN sample and support the reliability of our NGS-analysis.

## Non-Olfactory GPCRS (nGPCRS)

Our analysis allowed us to detect the transcripts of known and previously undescribed membrane proteins in the OE. GPCRs form the largest family of transmembrane proteins and function in various signaling pathways. Out of 407 annotated non-olfactory GPCRs (nGPCRs) in our data set (S7 Table), we detected the expression of 114 (sorted ORNs) and 159 (male/female OE) nGPCRs, which displayed FPKM values >1 (Table 2).

**Table 2:** The total number of GPCRs detected in mouse OE (RefSeq gene model).

	**ORNs (heterozygous)**	**OE1 CD1 male**	**OE1 CD1 female**	**Number of genes annotated in RefSeq gene model**
Chemosensory GPCRs				
ORs	905	1,001	1,075	1,225
TAARs	13	11	13	15
Vomeronasal receptors 1	24	5	4	218
Vomeronasal receptors 2	20	4	5	130
Taste receptors	6	3	5	38
Non-chemosensory GPCRs	238	264	250	407
Total	1,206	1,288	1,352	2,033

**Figure 6:**
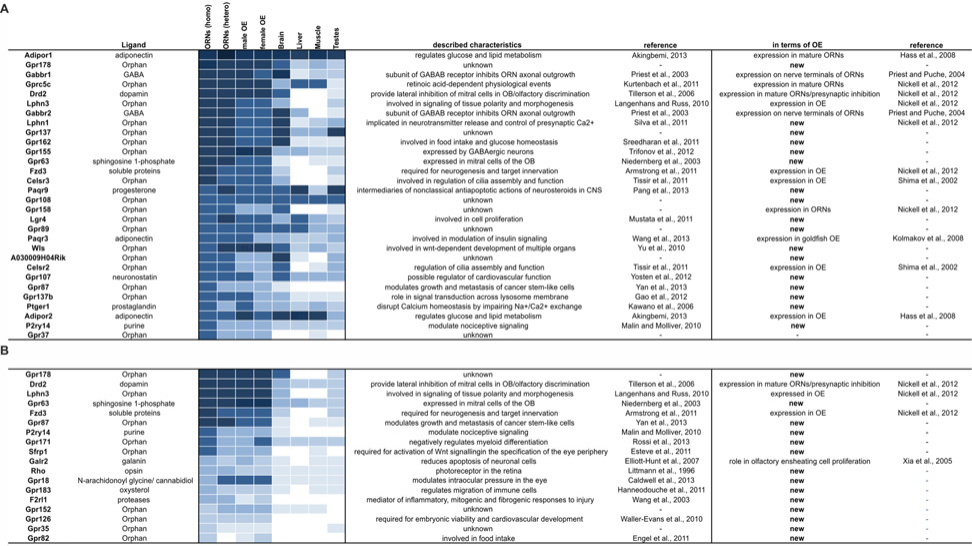
Expression ranking of non-olfactory GPCRs (nGPCR). **A.** Heatmap showing the ranking of the 30 most highly expressed nGPCRs in the FACS-sorted ORNs. Only six nGPCRs were among the 1,000 most highly expressed genes (Adipor1, Gpr178, Gabbr1, Gprc5c, Drd2, and Lphn3). Of the 30 most highly expressed genes, the expression of 17 in the OE was no previously known. **B.** Heat map showing the ranking of nGPCRs that are specifically enriched in ORNs according the criteria that the nGPCRs’ FPKMs were greater than 1 and 5 times greater in the ORNs than in non-olfactory tissue (brain, liver, muscle and testes). A total of 18 specifically enriched nGPCRs were found in ORNs. Excluding the specifically enriched candidates that were already presented in the list of the 30 most highly expressed genes, an additional 10 genes were found to be specifically enriched and are new in terms of olfaction. Regarding the genes that were among the 30 most highly expressed and were specifically enriched in ORNs, 60% had neither been shown to be expressed in the OE or been ascribed any function in the OE in any previous study with the exception of for several candidates in a tabular form in a recent transcriptome-wide study of the total OE [52].

We classified the nGPCRs into five main GPCR families: glutamate, rhodopsin, adhesion, frizzled and secretin. This classification reveals that the nGPCRs (including genes with FPKMs below 1) that were expressed in ORNs mainly belong to the rhodopsin family (64%). Ten percent belong to the adhesion family, approximately 4% to the frizzled family, 6% to the glutamate family, and 0.2% to the secretin family. An additional set of nGPCRs with unknown functions, no known ligands and atypical structure could not be classified (approximately 12%) nGPCRs that are highly expressed may hypothetically function as co-receptors in ORNs [86, 87]. Therefore, we created a ranking of the expression of all nGPCRs to identify the highly expressed candidates (Fig. 6). Interestingly, only six nGPCRs were ranked among the 1,000 most highly expressed genes in ORNs: Adipor1, Gpr178, Gabbr1, Gprc5c, Drd2, and Lphn3 (Fig. 6). An additional 30 nGPCRs exhibited medium expression profiles (FPKMs between 52–10), and 65 nGPCRs exhibited low expression profiles (FPKMs between 1–10) (S11 Fig.). Some of these GPCRs have previously been reported to be expressed in ORNs [48, 49]. Regarding the most highly expressed 30 nGPCRs, our study describes the expression of an additional 17 genes: Gpr178, Lphn1, Gpr137, Gpr162, Gpr155, Gpr63, Paqr9, Gpr108, Lgr4, Gpr89, Wls, A030009H04Rik, Gpr107, Gpr87, Gpr137b, Ptger1 and P2ry14 (Fig. 6). A few of these genes have also been tabularly presented in a recent transcriptome wide study of the total OE [52].

In a subsequent analysis, we classified the nGPCRs according to their expression levels and expression patterns. To identify nGPCRs that were specifically expressed in ORNs, we compared expression levels between ORNs and non-chemosensory tissue. We defined a gene as being enriched in ORNs, if the expression level of that gene was at least five times higher (as determined by RNA-Seq data) in the ORNs than in any of the control tissues (brain, liver, muscle and testes) (S8 Table). We found that 14 (FPKM > 1) nGPCRs were specifically enriched in ORNs, and 100 nGPCRs had broader tissue distributions (Fig. 6 and S7 Table).

Focusing on nGPCRs specifically enriched in ORNs and excluding genes already presented under the 30 top expressed candidates, we additionally detected the expression of 10 genes: Gpr171, Sfrp1, Rho, Gpr18, Gpr183, F2rl1, Gpr152, Gpr126, Gpr35 and Gpr82.

**Figure 7:**
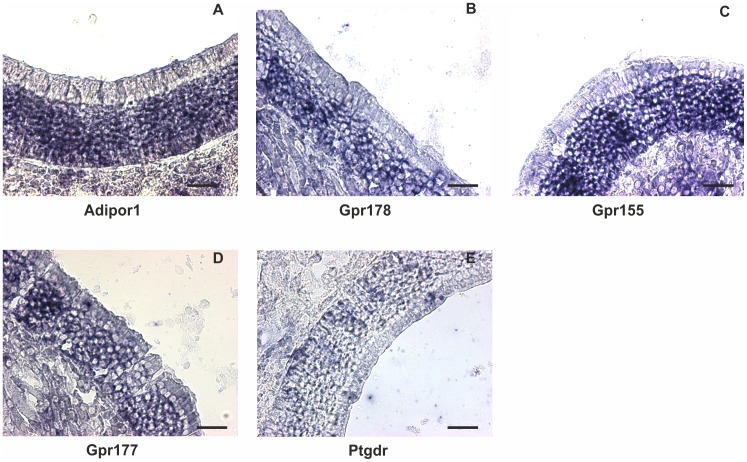
*In situ* hybridization for nGPCRs mRNA. A: Adipor1 (adiponectin receptor 1) B: Gpr178; C: Gpr155; D: Gpr177 (aka Wls); E: Ptgdr (prostaglandin D receptor). All transcripts were detected in the mature ORN cell layer as predicted by the expression levels observed in sorted ORNs. Scale bar = 30 µm.

**Figure 8:**
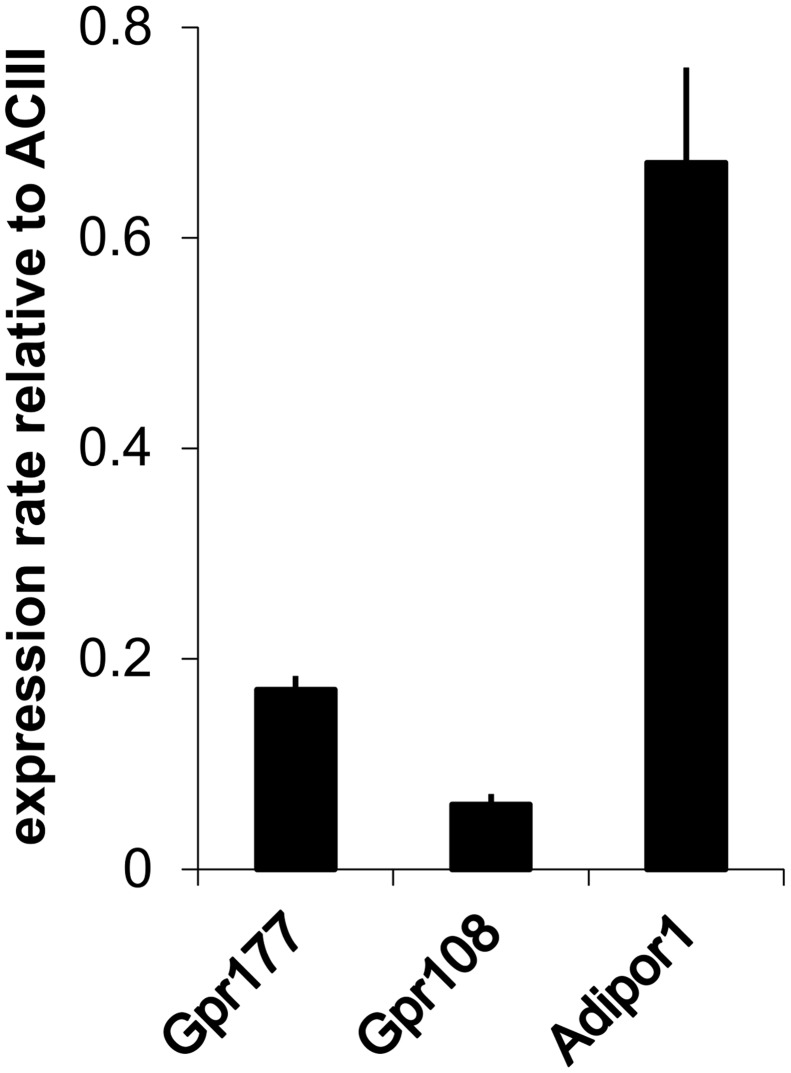
Expression levels of GPCRs by real time RT-PCR. Bar chart shows the relative expression levels of selected GPCRs relative to adenylyl cyclase type III (ACIII) as determined by RT-PCR. In accordance with our RNA-Seq data, the most highly expressed GPCR was Adipor1. Error bars represent the SEM.

Finally, we constructed a quantitative ranking of the expression of all GPCRs detected in the OE and ORNs (S7 Table). We selected some of these new nGPCRs and verified their expression in ORNs with *in situ* hybridization (Fig. 7). All selected probes produced signals in the mature ORN layer as predicted by the FPKM values from ORNs. Additionally, we tested the most highly expressed GPCR, Adipor1 and two other identified GPCRs with RT-PCR and determined their relative expression levels (Fig. 8). As expected, we found that Adipor1 was most highly expressed followed by a lesser extent for Gpr177. These results match our RNA-Seq and *in situ* hybridization results.

## Non-GPCR Membrane Proteins

The detection of unknown transmembrane proteins in several recent proteome studies has led to the discovery of proteins with essential functions in olfactory signaling processes. For example, Ano2 was identified as the olfactory CaCC [16, 15].

To identify other potential candidates, we searched for new non-GPCR membrane proteins that were highly and/or specifically expressed in ORNs. We assembled a catalogue of integral membrane proteins by manual inspection and GO terms (integral to membrane: GO: 0016021) using *Ontologizer* [88]. This process led to the detection of the expression (FPKMs > 1) of up to 2,339 genes in ORNs (S8 Table), 2,706 in male OE, and 2,575 in female OE. We next ranked these genes based on their expression levels and expression patterns.

**Figure 9:**
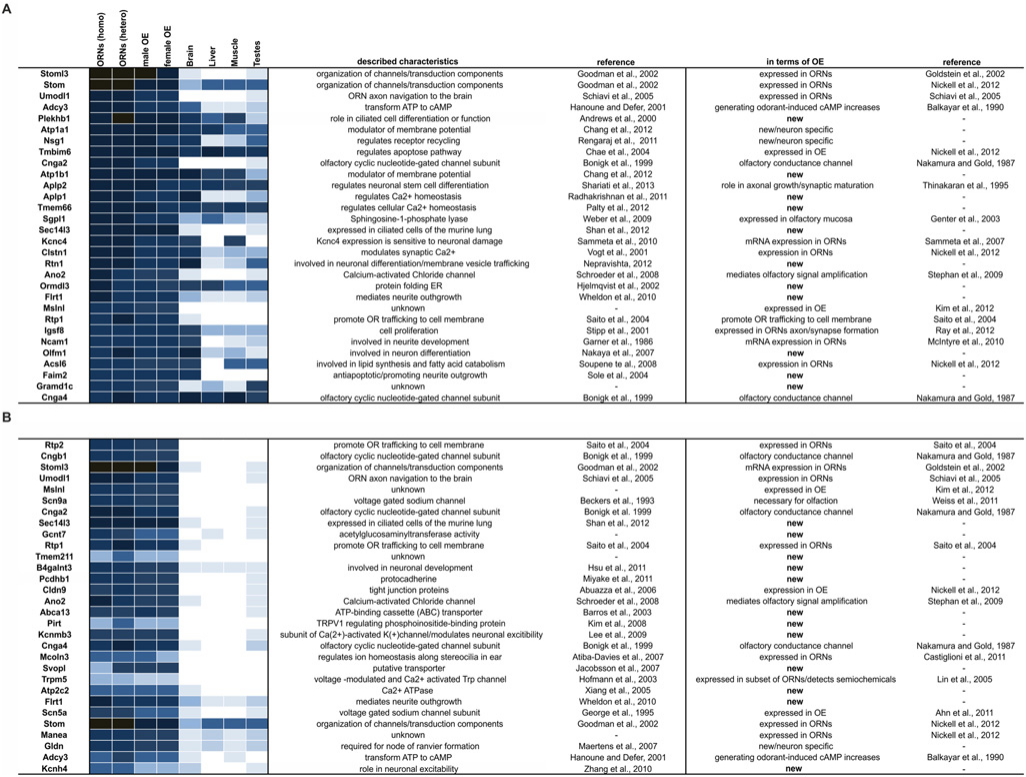
Expression patterns and ranking of genes coding for non-GPCR membrane proteins. **A.** Heatmap showing the ranking of the 30 most highly expressed genes in the FACS-sorted ORNs. **B.** Heatmap showing the ranking of the 30 most highly expressed genes that were specifically enriched in ORNs according to criteria that their FPKMs > 1 and their expression level in ORNs was at least 5x greater than that in non-olfactory tissue (brain, liver, muscle and testes).

This ranking showed that, of the 1,000 most highly expressed genes, 20% were genes that code for membrane proteins. We found the following known components of basic signaling among the 30 most highly expressed membrane protein genes: ACIII, Cnga2, Cnga4 Ano2, and Rtp1, which is involved in OR trafficking [21]. In addition to these genes that are known to have roles in basic signaling, we found Stoml3, Stom, Umodl1, Plekhb1, Atp1a1, Nsg1, Tmbim6, Atp1b1, Aplp2, Aplp1, Tmem66, Sgpl1, Sec14l3, Kcnc4, Clstn1, Rtn1, Ormdl3, Flrt1, Mslnl, Igsf8, Ncam1, Olfm1, Acsl6 and Faim2 (Fig. 9).

Out of these 30 most highly expressed genes in ORNs, we detected 11 genes (Fig. 9), whose expression was not reported in the OE. That these genes ranked among the 200 most highly expressed genes indicates their potential importance in olfaction.

Among the top 30 specifically enriched genes, we also found candidates that were among the 30 most highly expressed genes overall: Stom, Stoml3, Umodl1, Cnga2, Ano2, Rtp1, ACIII and Mslnl. Further known components of olfactory signal transduction that specifically were enriched in ORNs were the other subunits of the CNG channel (Cngb1, Cnga4), Scn9a, Scn5a and Trpm5, which is involved in the detection of semiochemicals [89]. Additionally, the following genes exhibited specifically enriched expression patterns: Sec14l3, Gcnt7, Tmem211, B4galnt3, Pcdhb1, Cldn9, Abca13, Pirt, Kcnmb3, Mcoln3, Svopl, Atp2c2, Flrt1, Manea, Gldn and Kcnh4.

We next focused on genes involved in the transport or flux of ions across the membrane.

We detected 132 ion channels, 199 members of the solute carrier (SLC) superfamily and 122 active transporters in ORNs with FPKMs >1 (S10 Table).

Our analysis revealed that the CNG channel subunits, Ano2, Scn9a and Scn4b were among the 10 most highly expressed channel genes in ORNs. Several potassium channel genes showed relatively high and specific expression patterns in ORNs. The most highly expressed was Kcnc4, the expression of which in ORNs has also been reported by Sammeta et al. (2007) [48].

In addition to Kcnc4, we detected two members of the voltage-gated KCNH channel family, Kcnh3 and Kcnh4 and several members of the KCNA family: Kcna1, Kcna2, Kcna5, Kcna6 and Kcna10. Interestingly, the expression of these genes in ORNs has not previously been reported (S10 Table).

As mentioned above, the expression levels of Ano2 were high in ORNs. We also detected other members of the anoctamin family, namely Ano1, 3, 6, 7, 8, 9 and 10 (S10 Table). A small subset of the OE RNA-Seq data for anoctamines 1–10 and other genes (Ttyh1–3, Trpa1, Trpm8 and Trpv1) were previously published by Schöbel and colleagues (2013) [90] in a Heat Map figure.

Members of the transmembrane channel-like protein (TMCs) family are evolutionarily related to the anoctamines [91]. We detected expression of Tmc4, 5, and 7 in ORNs (S1 Table). We further identified the expression of up to 7 aquaporin genes; Aqp3 was strongly expressed in ORNs (S10 Table). Hcn2, a channel protein that is abundantly expressed in the olfactory bulb [92], was also detected.

We next focused on SLC members that were specifically expressed in ORNs (FPKMs >1 and 5x greater expressing in ORNs than in non-olfactory tissue) and have reported function in ion homeostasis. We detected Nkcc1 (Slca12a2), which is important for Cl^-^ accumulation in ORNs [18, 19], Nckx4 (Slc24a4), a Na^+^/Ca^2+^-exchanger involved in rapid response termination and adaptation of the olfactory response [42], and Nckx2 (Slc24a2). Our data also revealed the expression of Slc8a1, a Na^+^/Ca^2+^ exchanger, in ORNs [93]; this exchanger may contribute to the regulation of Ca^2+^ flux in ORNs (S10 Table).

Interestingly, we, for the first time, report the detection of the specific expression of additional membrane proteins in ORNs/OE; these novel proteins ABC-transporters, ATPases, tetraspanines, TMEM-proteins, WD-repeat domain proteins, Gram domain-containing proteins and several other proteins with unknown functions (Table 3).

**Table 3:** Further genes for non-GPCR membrane proteins that were among the 1,000 most highly expressed genes.

ABC transporter	Abca13, Abca5, Abcc4, Abcc1, Abcg1, Abcc10, Abca7
further ATPases	Atp6v1b1, Atp2c2, Atp6v1c2, Atp10d, Atp6v0a4, Atp13a5, Atp11a, Atp2c1
other subunits of pumps or transporter	Slco15a2, Sec14l3, Slc44a2, Slc15a2, Slc27a2, Slc22a20, Slc6a6
tetraspanines	Tspan3, Tspan13, Tspan7
transmembrane proteins	Tmem66, Tmem64, Tmem59, Tmem30a, Tmem63b, Tmem151b, Tmem50a, Tmem205, Tmem213
WD-repeat domain proteins	Wdr17
GRAM domain containing proteins	Gramd1c
other proteins	Tusc5, Dnajb14, Efr3b, Gpm6a, Homer2, Tm9sf3 Ttc9, Nehrf1, Nherf2

**Figure 10:**
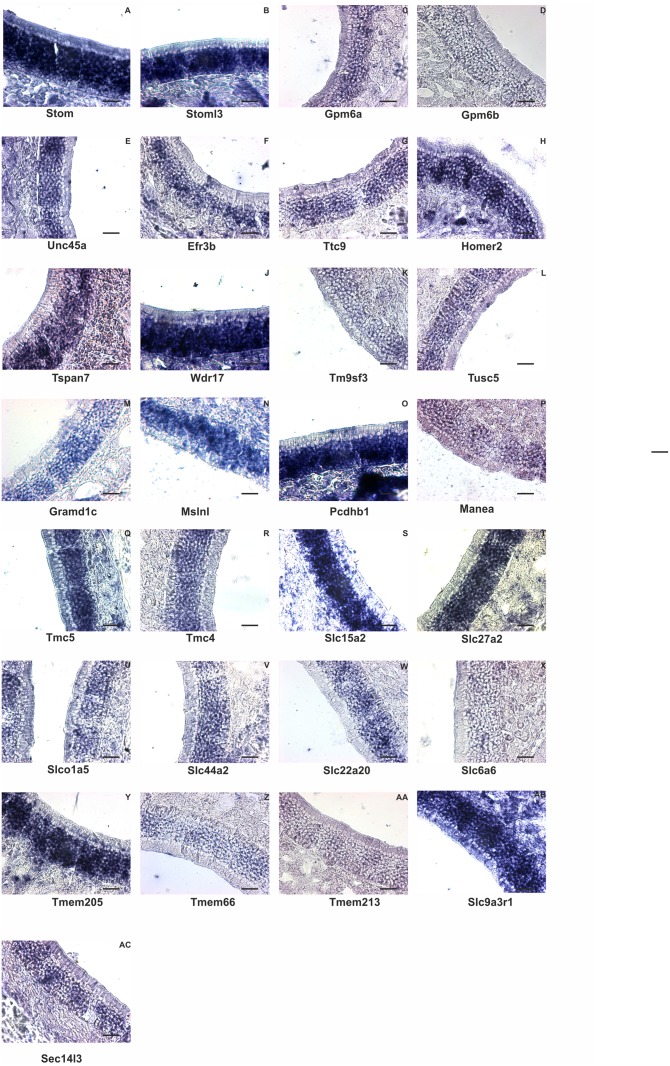
*In situ* hybridization for mRNA of non-GPCR membrane proteins. A: Stom (stomatin), B: Stoml3: Stomatin-like protein 3, C: Gpm6a (glycoprotein m6a), D: Gpm6b (glycoprotein m6b), E: Unc45a (protein unc-45 homolog A), F: Efr3b (EFR3 homolog B), G: Ttc9 (tetratricopeptide repeat domain 9), H: Homer2 (homer protein homolog 2), I: Tspan7 (tetraspanin 7), J: Wdr17 (WD repeat domain 17), K: Tm9sf3 (transmembrane 9 superfamily member 3), L: Tusc5 (tumor suppressor candidate 5), M: Gramd1c (GRAM domain-containing protein 1), N: Mslnl (mesothelin-like protein precursor), O: Pcdhb1 (protocadherin beta 1), P: Manea (glycoprotein endo-alpha-1,2-mannosidase), Q: Tmc5 (transmembrane channel-like gene family 5), R: Tmc4 (transmembrane channel-like gene family 4), S: Slc15a2 (solute carrier family 15 (H+/peptide transporter), member 2), T: Slc27a2 (solute carrier family 27 (fatty acid transporter), member 2), U: Slco1a5 (solute carrier organic anion transporter family, member 1a5), V: Slc44a2 (solute carrier family 44, member 2 / choline transporter-like protein 2), W: Slc22a20 (solute carrier family 22 member 20), X: Slc6a6 (sodium- and chloride-dependent taurine transporter), Y: Tmem205 (transmembrane protein 205), Z: Tmem66 (transmembrane protein 66), AA: Tmem213 (transmembrane protein 213), AB: Slc9r3a1 (solute carrier family 9 (sodium/hydrogen exchanger), member 3 regulator 1, AC: Sec14l3 (Sec14-like protein 3). All transcripts were detected in the mature ORN cell layer as predicted by the expression in sorted ORNs. Scale bar = 30 µm

We selected several genes and confirmed their expression patterns with ISH (Fig. 10). All probes produced positive signals in the mature ORN layer.

## Classes of Transcripts Enriched in ORNs

To obtain an overview of the transcript classes that were enriched in ORNs, we analyzed the gene expression patterns according to gene ontology (GO) categories using the Ontologizer software [88].

Previous microarray and proteome studies have described several transcript classes that are enriched in ORNs [48, 49, 54, 55, 53, 56, 16].

**Figure 11:**
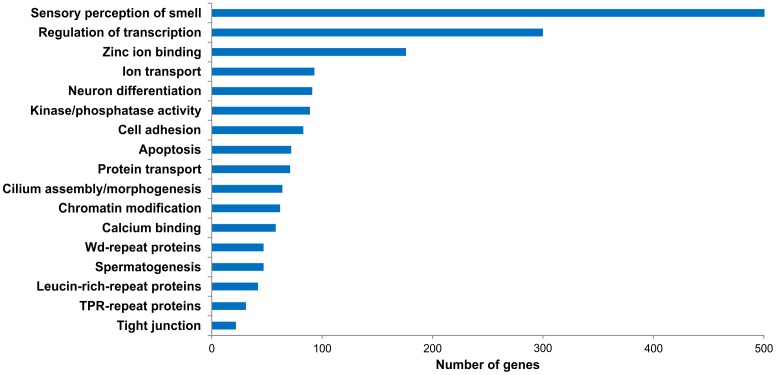
Classification of GO terms enriched in ORNs. Based on results from Ontologizer [88], specifically enriched GO terms are represented in this bar chart. For simplification, related classes were combined.

Genes associated with the sensory perception of smell (including ORs, OMP, Gα_olf,_ and ACIII) represented the predominant class/GO term (GO: 0007608) in our list (followed by the GO categories of ion transport (GO:0006811) and cilia morphogenesis (GO:0005929) (Fig. 11). Proliferating basal cells continuously replace dying or aging ORNs in the OE. Thus, it is not surprising to find that transcripts for neuronal differentiation (GO:0030182) are enriched in the OE [84]. The Ca^2+^-dependent odorant induced excitation and adaptation process [94] is the origin for the class/GO-term (GO:005509) that includes genes that encode proteins with calcium binding properties. Biological processes, such as protein transport (GO:0015031), RNA-processing (GO:0006396), and chromatin modification (GO:016568), are ubiquitous functions in cells. The corresponding categories contain the genes that support basic cellular mechanisms [95].

Genes classifications that include ubiquitously expressed genes but are also likely to include genes that are more restricted to the machinery of ORNs [48] include the following: regulation of gene expression (GO: 10468), zinc ion binding (GO:0008270), kinase (GO:0016301) or phosphatase activity (GO:0016791), cell adhesion (GO:0007155), calcium binding (GO:0005509), TPR (GO:0030911), and WD repeat (GO:0005515).

## Genes Related to cAMP-Signaling

**Figure 12:**
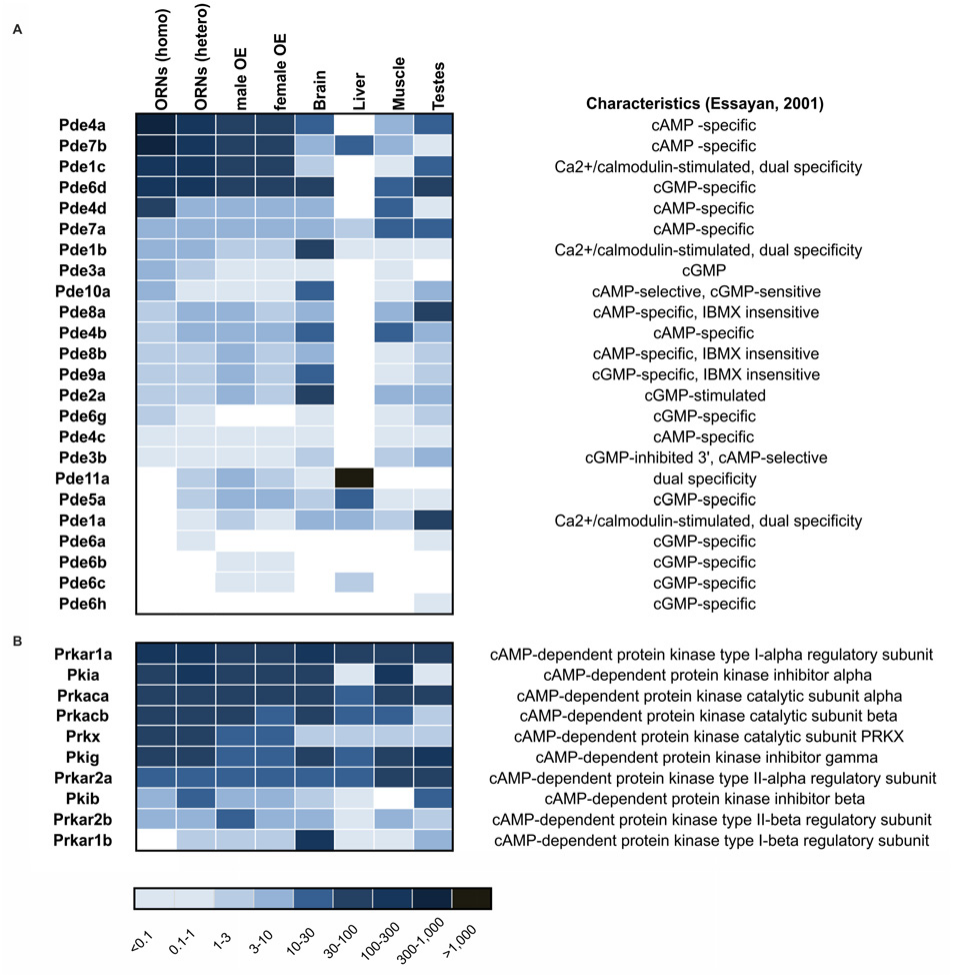
Expression patterns of genes involved in cAMP-dependent signaling. **A.** Expression patterns of cyclic nucleotide phosphodiesterase (PDE) genes. Heatmap showing the expression levels of PDEs in olfactory tissue and non-olfactory tissues (brain, liver, muscle and testes). **B**. Expression patterns of cAMP-dependent protein kinases and associated regulatory proteins in olfactory tissue and non-olfactory tissues (brain, liver, muscle and testes). Higher FPKM values are indicated by deeper colors.

In addition to the highly expressed G_αolf_, ACIII and CNG channel subunits, we analyzed the expression patterns of other proteins involved in cAMP signaling. We detected strong expression of phosphodiesterases (PDEs), which are possible candidates for rapid termination of olfactory signal transduction due to their action in the degradation of the second messenger cAMP [22, 96]. Previously, three PDEs, Pde1C [97, 34], Pde4A [35] and Pde2 [98] had been identified in mammalian ORNs. Our transcriptome data revealed the expression of new PDEs in ORNs and the OE (Fig. 12). Our dataset demonstrated that all 11 PDE gene families are represented by the expression of at least one gene in ORNs and/or the OE. We report for the first time that Pde7b is expressed in ORNs and that it is one of the three most highly expressed PDEs in ORNs. Pde7b is a cAMP-specific phosphodiesterase that may have a modulating effect in olfactory signal transduction via its ability to hydrolyze cAMP. Moreover, we discovered that the Pde6d subunit is also highly expressed. Pde6 is localized in rod and cone photoreceptors where it regulates cytoplasmic cGMP concentrations [99, 100]. The high level of expression of the cGMP-specific Pde6d raises the question of what impact cGMP has on olfactory signal transduction. The IBMX-insensitive PDEs Pde8 (Pde8a, Pde8b) and Pde9 (Pde9a) were detected at low expression levels in ORNs and the OE.

Additionally, we detected high levels of expression of cAMP-dependent protein kinases (Prk), which form tetramers that consist of 2 regulatory and 2 catalytic subunits [101]. Regarding the catalytic subunits, we detected the alpha, beta and Prkx subunits, and we detected 4 isoforms the regulatory subunit (alpha I/II and beta I/II). Moreover, we detected weak expression of 3 cAMP-dependent PKA inhibitor genes (Pkia, Pkib and Pkig) that regulate the action of PKAs (Fig. 12).

## Genes Related to IP3 / PI Signaling

The physiological function of phosphatidylinositol (PI) signaling mechanisms in ORNs have been discussed for several years [45]. Recent findings suggest that PI3K-dependent signaling mediates the inhibition of odorant responses in ORNs, which express GPCR-activated isoforms of PI3K and exhibit odorant-induced PI3K activity [43, 102, 103, 47]. Several PI-kinases (S11 Table) were found in ORNs. The expression of the phosphatidylinositol 3-kinase type catalytic subunits PI3K-β and γ were of special interest because these subunits can be activated by the β/γ subunits of G-proteins, and both have been detected in the OE [103]. Our sequencing data revealed that ORNs mainly express PI3kα and PI3kβ and, to a lesser extent, PIk3γ. Nearly all subtypes were expressed more highly in ORNs than in non-OE control tissues (S11 Table); however, the expression levels of these kinases were only moderate compared to the expression of elements of cAMP signaling. Nevertheless, all four types of PI3-kinases were detected in ORNs by *in situ* hybridization (S12 Fig.).

Phospholipase A2 (XIIA), phospholipase C, (b3, 4) phospholipase C-like 2 and phospholipase D3 are the most highly expressed phospholipases, but none of these are specific to ORNs (S11 Table). The same is true for the most highly expressed PKCs. The expression of IP3-receptors was previously shown by Restrepo et al. (1990) [104], Fadool and Ache (1992) [105], Kalinoski et al. (1992) [106], Restrepo et al. (1992) [107], Cunningham et al. (1993) [108] and Munger et al. (2000) [39]. Among the three subtypes, IPTr1 is most highly expressed, although its expression levels are about 100-fold lower level than those of the CNG channels. In contrast to genes involved in the cAMP-mediated signaling pathway, none of the genes involved in PI signaling were among the 500 most highly expressed genes.

## Expression Patterns of TRP Channels

**Figure 13:**
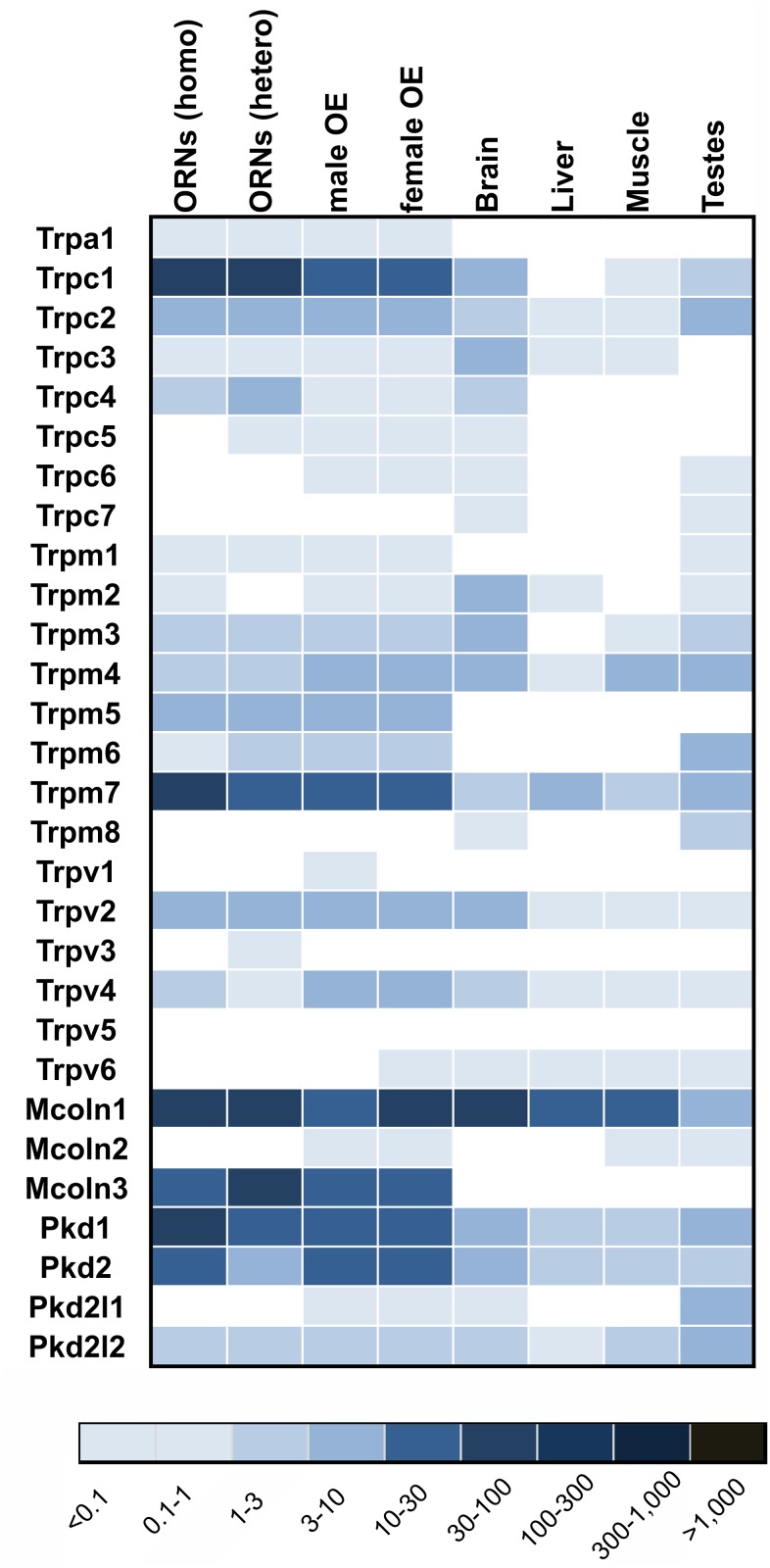
Expression pattern of TRP channel genes. Heatmap showing the expression levels of annotated TRP channels in olfactory (male and female OE, and FACS-sorted ORNs) and non-olfactory tissues (brain, muscle, liver and testes). Higher FPKM values are indicated by deeper colors.

TRP channels constitute a family of proteins that respond to a variety of stimuli [109]. The expression of several TRP channels has been reported in the OE [110, 89]. Our sequencing data provide a comprehensive overview of the expression levels of TRP channel transcripts in both the OE and FACS-sorted ORNs (Fig. 13). Within the TRPC subfamily, TRPC1, TRPC2 and TRPC4 were detected in ORNs, and TRPC1 transcripts were the most enriched. While the transcripts of seven TRPM subfamily members were amplified from pooled cDNAs of whole OE, only four members showed somewhat higher expression levels in ORNs (Fig. 13). Transcript levels of TRPM7, a channel fused to a protein kinase [111], were the highest. Among TRPV subfamily members, only TRPV2 transcripts were present in ORNs. We also detected transcripts for two of the intracellular mucolipin TRP proteins, TRPML1 (Mcoln1) and TRPML3 (Mcoln3). Finally, and consistent with previous studies [112], we found both PKD1 and PKD2 transcripts in ciliated ORNs.

**Figure 14:**
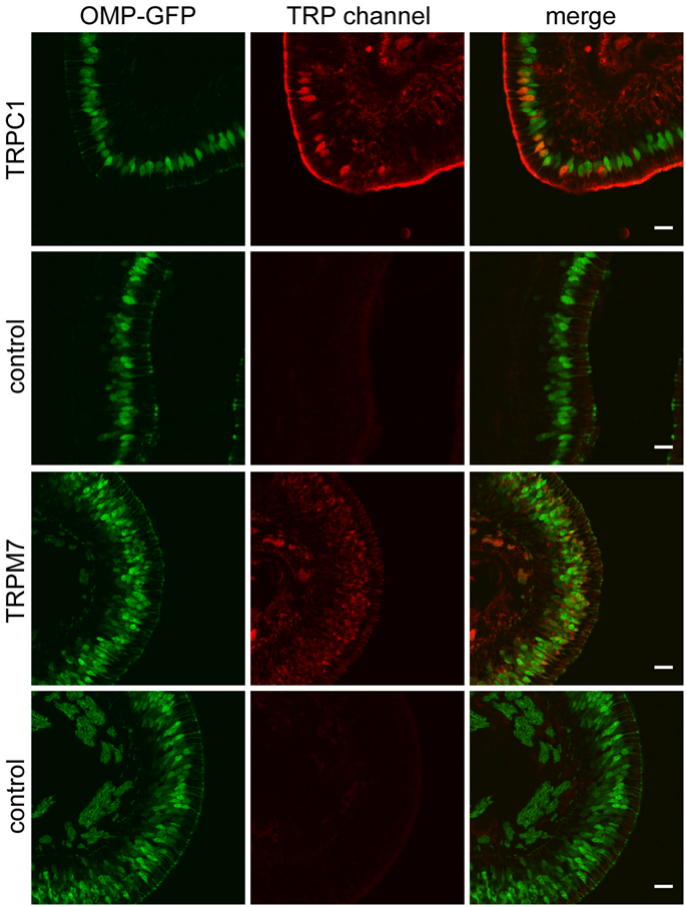
Expression of TRPC1 and TRPM7 in the OE. Protein expression was assessed staining coronal sections of OMP-GFP mice with anti-TRPC1 and TRPM7 antibodies. TRPC1 immunoreactvity was observed in a few ORNs in 1 of 3 stained sections, while TRPM7 immunoreactivity was detected in a larger number of ORNs (1 stained section). Omission of the primary antibody served as control. Scale bar, 20 μm.

TRPC1 and TRPM7 transcripts were among the most enriched TRP channel transcripts in ORNs, yet they constituted only ~ 7% of the total number of CNGA2 channel subunit transcripts. We examined the expression by immunohistochemistry and detected TRPC1 and TRPM7 immunoreactivity in ORNs (Fig. 14).

## Discussion

A comprehensive understanding of the transcriptome is fundamental for the study of the functionality of ORNs and the machinery of their crucial elements. Several previous studies, including microarray [66, 48, 113, 49] and proteomics studies [53, 55, 56, 16, 15, 54], have revealed the expression of a large number of known and novel genes in the OE. While proteomic studies have facilitated progress, for example, the identification of the Ano2 gene that encodes the olfactory CaCC, most of these studies lack proper quantification of genes and remain incomplete, especially regarding the detection of OR gene expression. Recent advances in next generation sequencing provided the means to obtain a “complete” list of protein-coding genes expressed in the OE in a high-throughput fashion.

Here, we present a comprehensive analysis of the murine olfactory transcriptome that was generated by the high-throughput NGS technique. For the first time, we analyzed FACS-sorted ORNs to gain knowledge about the differences in expression profiles between ORNs and other cell types in the OE with the NGS technique.

We compared our data to transcriptome data from non-olfactory tissues (brain, liver, muscle and testes) to identify ORN-specific genes. We further described the expression of known and novel membrane proteins and generated a list of all non-olfactory GPCRs that were expressed in the OE.

During the preparation of our manuscript, a murine olfactory transcriptome study was published that focused mainly on sex-specific differences [51] in the OE. Using NGS, these authors detected nearly all classified OR genes. A second study by Keydar et al. (2013) [52] focused on a more general catalogue of genes expressed in the OE, but did not provide any data on specificity of expressed genes for ORNs.

Our analysis, and particularly our comprehensive list of genes expressed in ORNs, complements previous studies and provides a basis for the discovery and study of novel genes expressed in the OE, especially ORNs.

## Chemoreceptors

In the mouse, the OR family comprises ~1209 OR genes, of which 913 have been identified as functional genes and 296 have been identified as OR-pseudogenes [1]. Using NGS, we successfully detected up to ~97% (FPKM > 0.1) of all OR genes based on the Refseq gene model in our probes. A previous study by Shiao et al. (2012) [51] detected 99% of all OR genes, and this result was possibly due to the even greater sequencing depth of that study. The fact that virtually all ORs are highly expressed in the OE and virtually none are expressed in other tissues (except for the testes) is astonishing for a gene class with ~1,200 members and suggests an extremely thorough regulation of gene expression.

Sequencing of the FACS-sorted ORNs detected the expression of fewer OR genes, and this was likely due to the lower number of sequences generated for homozygous ORNs (~13 million) and the limited number of sorted neurons. As we used OMP-GFP transgenic animals to obtain ORN samples for sequencing, we cannot exclude the possibility that some receptors were underrepresented. OMP is a marker of mature olfactory neurons [114]. The expression level of a particular OR gene is strongly dependent on the receptor type. In CD1 mice, the expression patterns of OR genes were strongly correlated between female and male OEs (Pearson coefficient, r = 0.83). The expression rankings for the OR genes Olfr533, Olfr1507 and Olfr309 were similar in male and female mice, and the three most highly expressed OR genes found in the sorted ORNs were among the ten most highly expressed OR genes in the OE. That similar percentages of OR genes were detected in our and previous studies suggests that the OR subgenome does not only strongly correlate between sexes, but also between mouse strains because OE samples were derived from female CD1 and C57BL/6J mice (S8 Fig., S9 Fig.). We calculate a Pearson coefficient of r = 0.75 for between these two groups, which suggest that only a small percentage of OR genes is strain-specific and differentially expressed. However, further studies are needed for detailed analyses of strain-specific differences in the OR subgenome.

One of the most highly expressed OR genes, Olfr1507, was also the most transcribed OR gene in the FACS-sorted ORNs sample in our study (FPKM ~ 97) [115]. The number of ORNs expressing an individual OR varies and is coupled to the strict regulation of gene choice [2]. Olfr1507 (also called MOR28) is located in a gene cluster consisting of seven ORs and an upstream H regulatory element on chromosome 14. The H element interacts with the most proximal OR gene (Olfr1507) and may facilitate its expression [116]. The expression levels of the remaining 6 genes in the cluster (Olfr1508, Olfr1509, Olfr1510, Olfr1511, Olfr1512) are considerably lower, suggesting that the most proximal position to the regulatory H element indeed defines the expression strength of these ORs in the OE.

## Other Chemoreceptors

In addition to ORs, we detected the expression of other types of chemoreceptors in the OE. Mice have 15 TAAR genes, 14 of which are expressed in the OE [71]. We detected all 14 TAAR genes in the OE, which underlines the quality of our data set. Interestingly, we also detected the expression of vomeronasal receptors in the OE. We identified the expression of 15 V1R and 5 V2R genes in the OE and 24 V1R and 21 V2R genes in FACS-sorted ORNs. The FPKM values were often small (< 1) and comparable to the minimally expressed ORs, suggesting a possible mosaic-like gene expression pattern for these chemoreceptors in only a few cells in the OE. The expression of the V1- and V2R genes in ORNs implies that the main OE contributes to pheromone detection. Interestingly, in previous studies, pheromone-induced behavior was not altered in mice after removal of the VNO [117], and electrophysiological data additionally support the detection of several pheromones in the OE [118]. Although we also detected the expression of TRPC2 and PLC (both of which are part of the transduction cascade in VNO neurons) [119–121] in our transcriptome analysis of the OE, the low number of detected vomeronasal receptors likely limits their contribution to OE-mediated pheromone sensing. Thus, it seems reasonable that ORs or other receptor classes are also involved in pheromone perception in the OE.

We also detected an enrichment of two taste receptors in ORNs, Tas1r1 and Tas1r3, which are known to heterodimerize and subsequently form the umami-receptor. The detection of taste receptors in the OE could likewise confirm the presence of solitary chemosensory neurons (SCN) in the OE, which are known to express TasRs [122]. Chemosensory information derived from taste and olfaction is used by organism to value the quality of food. The detection of taste receptors in the OE may underline the fact that the sense of olfaction and taste are linked modalities.

## Signal Transduction in ORNs

The commonly known components of the classical olfactory signal transduction pathway were among the 200 most highly expressed genes in our murine OE transcriptome analysis.

It is reasonable to assume that other global players in olfactory signaling should be as highly expressed. In this respect, we detected several GPCRs and other membrane proteins with high expression levels. Their relevance to olfactory signaling remains elusive. Future studies may uncover the function of the presented candidate genes in ORNs.

Among the 200 most highly expressed genes were the previously unrecognized phosphodiesterases Pde6d and Pde7b. Previous studies suggest that hydrolyzation of cAMP by phosphodiesterases is involved in the termination of the olfactory signal transduction. The Ca^2+^/calmodulin-stimulated Pde1c appears to be enriched in the olfactory cilia, whereas the cAMP-specific Pde4a occurs throughout the cell but not in the cilia [34, 98]. Simultaneous disruption of the Pde1c and Pde4a genes in mice leads to prolonged response termination in electro-olfactogram measurements [97]. The potential regulatory function of this novel expression of Pde7b and Pde6d in the OE in olfactory signaling will be the subject of future studies. While Pde6 is primarily known to be localized to photoreceptors, where it regulates the cytoplasmatic cGMP concentration [99], less is known about the subunit Pde6d. Previous studies have reported a regulatory function of Pde6d in the membrane association of Ras and Rap GTPases [123] and a potential contribution to cell proliferation [124].

We additionally focused on molecules supporting alternative signaling pathways in ORNs. The involvement of odorant-stimulated PI signaling in ORNs has previously been shown [45, 43, 103]. This pathway involves PLC- and PI3K-dependent signaling. Our data provide support for a potential PI-mediated signaling of odorants as we detected weak expression of PLC, PI3K and IP_3_ receptors.

Interestingly, our data revealed the expression of several TRP channel transcripts. TRP channels are known downstream targets of PI-mediated signaling in chemosensory cells [125, 126]. The detection of semiochemicals is thought to involve Ca^2+^-activated TRPM5 channels that are expressed in a subset of ORNs in which both CNGA2 and PLC are co-expressed [89]. TRP channels form non-selective cation channels that are permeable to calcium, which may suggest a contribution to processes in the OE such as transduction, transcriptional control or proliferation. Among the detected TRP channel transcripts, TRPC1 and TRPM7 were the most enriched. Immunohistochemistry indicated expression of these proteins in ORNs, but their function in ORNs is unknown. A recent study proposed a regulatory function of TRPC1 in the fine-tuning of neuronal migration [127]. Another study by Kerstein et al. (2013) [128] addressed a mechanosensitive role of TRPC1 in spinal neuron growth cones. Additionally, TRPC1 has been proposed to be involved in store-operated Ca^2+^ entry, which regulates cell proliferation [129]. TRPM7 is ubiquitously expressed and has been implicated in a variety of cellular functions including magnesium homeostasis, cell cycle progression and control of the production of ROS [130]. Knock-out studies revealed a crucial role of TRPM7 in embryonic development [131, 132]. Future studies are required to examine potential functions of TRPC1 and TRPM7 in the OE.

## Non-Olfactory GPCRS (nGPCRS)

In this study, we present a comprehensive expression pattern analysis of GPCRs in the murine OE. In FACS-sorted ORNs, we detected the expression of 114 nGPCRs (FPKM>1). The expression of most of these proteins had previously been documented in at least one study that employed proteome or transcriptome analytic approaches [48, 49, 53, 55, 56, 16, 15, 54].

Our analysis revealed the expression of several unrecognized GPCRs for which no expression or functions in ORNs have been described. In summary, we detected the expression of 27 GPCRs, whose expression in ORNs has not been reported before, and 14 of these exhibited specific enrichment (FPKM >1 and an expression level that was at least 5 times greater than that in control tissues) in ORNs: Gpr178, Lphn1, Gpr63, Gpr137, Gpr162, Gpr155, Gpr87, Paqr9, Gpr108, Lgr4, Gpr89, Wls, A030009H04Rik, Gpr171, Gpr107, Gpr137b, Ptger1, P2ry14, Sfrp1, Rho, Gpr18, Gpr183, F2rl1, Gpr152, Gpr126, Gpr35 and Gpr82.

Interestingly, among 1,000 most highly expressed genes in ORNs, only 6 non-olfactory GPCRs were found: Adipor1, Gpr 178, Gabbr1, Gprc5c, Drd2, and Lphn3.

### GPCRs involved in food intake

The most highly expressed GPCR is Adipor1, which was first detected by Hass et al. (2008) [133] in mature ORNs. Another highly expressed adiponectin receptor is Paqr3 [134]. It is possible that adiponectin regulates food intake by acting as an appetite stimulator and conveying a starvation signal to the brain via AdipoR1 and AdipoR2 [135]. In animals, the sense of smell plays a crucial role in finding food resources. Thus, an interaction of adiponectin and AdipoR1 could modulate the function of ORNs depending on the nutritional status of the body. In this context, it is interesting to note that the modulatory effects of two anorectic peptides, insulin and leptin, on ORNs have already been demonstrated [136]. However, receptors for insulin, leptin, ghrelin and other peptides that are involved in the regulation of hunger and satiety were only weakly expressed in the OE (S13 Fig.); these findings lead to the assumption that adiponectin is the most important regulatory hormone in the OE. Therefore, future studies should concentrate on investigating the functions of AdipoR1 and Paqr3 and the modulatory effect of adiponectin on olfaction.

Furthermore, two orphan GPCRs, Gpr162, and Gpr82, are both possibly involved in food intake and glucose homeostasis [137, 138].

### mGluR family

Gprc5c was discovered in the mouse OE through proteomics [16], is an orphan receptor of the mGluR family [139], and is also expressed in goldfish OE [140]. Like Gprc5c, Gpr158 belongs to the mGluR family and is most similar to the metabotropic GABA receptors [141, 142].

### Inhibitory effect on olfactory signaling

Gabbr1, and the less-strongly expressed subunit Gabbr2, form the heterodimeric GABA_B_ receptor [143], which has been shown to inhibit ORN axonal outgrowth [144] and is possibly located presynaptically. Activation of GABA_B_ receptors, which couple to Gαi/o, stimulates increases in cAMP through βγ-mediated activation of adenylyl cyclase 2 and simultaneously inhibits Gαs-mediated activation of other adenylyl cyclases in the rat olfactory bulb [140, 145]. Supporting this GABAergic effect, Drd2 dopamine receptors are inhibitory on the input from ORNs and provide lateral inhibition of mitral cells, which provides olfactory discrimination in rodents [146]. Moreover Gpr63, a sphingosine 1-phosphate receptor, is expressed in mitral cells of the OB, but its function in this region in unknown [147].

Furthermore, many neurons that are classified as GABAergic might express GPR155 [148]. These findings imply that Gpr155 has an important role in GABAergic neurotransmission. GABAergic input has an inhibitory effect on olfaction [144]; thus, Gpr155 could also have a supportive function for the GABAergic effect on ORNs.

### Cell Architecture / Cell development

Additionally, we detected a few GPCRs that have possible roles in anatomical structural development.

The protocadherin receptors Celsr2 and Celsr3 are key regulators of the correct positioning of cilia and, consequently, cilia function [149]. The latrophilin receptors (Lphn1, Lphn3) are involved in signaling of tissue polarity and morphogenesis [150].

Fzd3 is required for neurogenesis and target innervation during sympathetic nervous system development [151].

Lgr4 is part of the Wnt signaling pathway that is involved in the cell proliferation of the intestinal epithelium [152].

### Orphan GPCRs with unknown functions

Furthermore, we detected several GPCRs, the functions of which are presently unknown, that were specific to ORNs.

Gpr178 (also named *Tmem181a*) is an orphan GPCR with unknown function that was not only among the 1,000 most highly expressed genes but was also specifically enriched in ORNs. This gene shares homologies with the newly identified Gpr177 (aka Wls) gene, which has been reported to be involved in the Wnt signaling pathway [153].

No endogenous ligands have been identified for Gpr137, Gpr152, Gpr158, Gpr89, Gpr171, Gpr108 or A030009H04Rik, the exact functions of these receptors are unknown.

We selected a few candidates and confirmed their expression in ORNs with ISH experiments: these experiments revealed that the mRNA transcripts of these candidates were prominent in olfactory neurons. Because these candidates were specifically expressed in mature ORNs and because of the described functions of these genes in other tissues, we suggest that they might play important roles in ORNs.

Previous studies have reported that ORs can co-assemble with other GPCRs such as beta adrenergic and muscarinergic receptors [86, 87]. However, according to our analysis, both of these GPCRs are only weakly expressed in ORNs. The six GPCRs we found to be highly expressed could form such co-receptors in principle, but our expression data did not reveal any candidates that were expressed at levels comparable those of the ORs. Consequently, the expression pattern of GPCRs contradicts the hypothesis of a GPCR co-receptor for ORs. In contrast, our data confirm that RTP1 and RTP2 are highly expressed; in addition to their known chaperone function, RTP1 and RTP2 have been reported to be required as co-receptors for ORs [21]. Our data support the hypothesis that RTP1 or RTP2 co-assemble with ORs in a stoichiometric manner because the expression levels of RTP1 and RTP2 are comparable that of the ORs.

Altogether, in our analysis, we provided a detailed expression ranking of all GPCRs detected in the OE and noted GPCRs new in terms of olfaction. Hence, our data directly leads to new perspectives to focus on so far unknown GPCRs. Due to a strong ORNs specific expression pattern; these GPCRs have a supposable important role in ORNs.

## Non-GPCR Membrane Proteins

As membrane proteins form key nodes in the olfactory signaling process, we focused on finding further membrane proteins that were not previously known to be expressed in, or have a specific function in, the OE. We detected the expression of 2,339 (FPKM > 1) genes for non-GPCR membrane proteins in ORNs. We ranked the expression of these genes and specifically marked those that were enriched in the OE compared to non-olfactory tissues to highlight genes that possible have important roles in the function of the ORNs. The main known components of olfaction (ACIII, CNG channels, Ano2, Rtp1) were found among the 30 most highly expressed genes.

Additionally, we identified 22 novel genes (11 that were specifically enriched in ORNs) that because of their high expression levels and their described functions in other tissues, are promising and important candidates for future research in olfaction: Abca13, Aplp1, Atp1b1, Atp2c2, B4galnt3, Faim2, Flrt1, Gcnt7, Gramd1c, Kcnh4, Kcnmb3, Olfm1, Ormdl3, Pcdhb1, Pirt, Plekhb1, Rtn1, Sec14l3, Svopl, Tmem211 and Tmem66.

### Transport

We newly detected the expression of a Na^+^/K^+^-transporting ATPase (Atp1a1, Atp1b1) in the OE; this ATPase modulates membrane potential [154]. We also detected Nsg1 in the OE; this molecule is involved in regulating receptor recycling [155]. The expression of both genes is important for olfactory signaling, as the adjustment of ion homeostasis is the basis for the depolarization of neurons and the recycling of receptors.

Further, the following transporter proteins were present: Abca13, an ABC transporter [156], Svopl, a putative transporter [157] and Atp2c2, a Ca^2+^ ATPase [158].

Aplp1 and Tmem66 are proteins that regulate the Ca^2+^ homeostasis [159, 160]. Rtn1 and Ormdl3 are involved in membrane vesicle trafficking and protein folding [161, 162], and these two processes are necessary for proper membrane expression of GPCRs and other membrane proteins.

Pirt, a phosphoinositide-binding protein, has been reported to function as a regulatory subunit of TRPV1 [163].

### Ion channels

It has been reported that 80–90% of the receptor currents that result from odorant exposure and OR interaction are mainly generated by Cl- ions, which pass through the CaCC Ano2. Billig et al. (2011) showed that EOG recordings from Ano2^-/-^ deficient mice were only reduced by up to ~40% [164]; nevertheless, these mice were able to smell. Therefore, it is highly probable that other CaCC channels are expressed in ORNs and that these channels, together with Ano2, mediate the major part of the receptor current. Although Pifferi et al. (2006) showed that the CaCC bestrophin-2 (Best2) is expressed in ORNs, wild type, and mice lacking Best2, exhibited no significant differences in olfactory ability [165]. Thus, Best2 is not a CaCC that makes a primary contribution to the odorant-induced chloride current. Therefore, we searched for new CaCC candidates that are important and account for olfactory transduction. In our analysis, we detected ORN-specific expression of genes coding for members of the TMC (transmembrane channel-like) protein family, which has an evolutionary relationship with anoctamines [91]. Based on expression patterns and the homology to anoctamines, we suggest that Tmc5, together with Tmc4 and Tmc7, are suitable candidates for the other CaCCs and could be important in olfaction. Future studies should examine the involvement of these proteins in olfaction.

Regarding voltage-gated sodium channels, we detected high levels of expression of Scn9a. These expression levels confirm the vital role of this channel in olfaction, as it has been shown that the loss of this protein leads to anosmia [82].

Among the variety of other K^+^ channels (S10 Table), the Kcnc4 was the most highly expressed voltage-dependent potassium channel subunit in ORNs. In the OE, the expression of Kcnc4 was already shown by Sammeta et al. (2010) [95], where they postulate a Kcnc4 expression sensitive to neuronal damage. Additionally, for the first time, we detected high and specific levels of expression of members of the voltage-gated Kcnh channel family, which is involved in the regulation of neuronal excitability [166]. Kcnh3 and Kcnh4, which are potentially members of the EAG-like (ELK) K^+^ subfamily, exhibited strong expression patterns in ORNs, indicating that these channels could be primarily responsible for determining and raising action potential thresholds by acting at voltages around the firing threshold to suppress excitability [166, 167]. Hagendorf et al. (2009) have previously demonstrated the expression of Kcnh channels in the sensory neurons of the VNO and their key roles in determining neuronal excitability [168]. Thus, it is conceivable that these candidate genes in ORNs have similar roles. As it has been shown that members of the ELK subfamily are able to co-assemble with each other [169], we further suggest that Kcnh3 and Kcnh4 could form functional heteromultimers and contribute to the hyperpolarizing effect in ORNs.

Similarly, strong and specific patterns of expression were observed for Kcna5, Kcna2, Kcna6, and a weaker pattern of expression was observed for Kcna1; these genes all code for members of the shaker-related voltage-gated K^+^-channel family. Interestingly, Eldstrom et al. (2002) reported that PDZ domains are able to bind to Kcna5 and other members of this family and affect potassium currents by regulating the assembly of the subunits [170]. Thus, it is of great interest to determine what impact this channel region has and if it interacts with Mupp1, which was recently identified as a PDZ-protein that is expressed in ORNs [28].

Moreover, we, for the first time, detected a medium-level expression of Kcnk12 and low-level expressions of Kcnk4, Kcnk5 and Kcnk1 in ORNs. In neurons, members of the two-pore domain potassium channel family Kcnk have been reported to determine membrane potentials and membrane input resistances, which influence the magnitudes and kinetics of responses to synaptic inputs [171]. Further focus should also be placed on Kcnk10, which was highly expressed, and Kcnk2, although this protein was expressed at lower levels. The described mechano- and thermosensitivity of these proteins [172, 173], is remarkable; these proteins are novel targets for the study of the physical impact of mechano- and thermosensation during the detection of odorants.

### Others

Of the remaining membrane proteins, we discovered that Mslnl is highly and specifically expressed in ORNs. No studies of the function of Mslnl in ORNs or any other tissue exist; Mslnl is a mesothelin-like protein. The related mesothelin protein is known to have cell adhesive properties [174]. Additionally, our data revealed that, in addition to Gpm6b, Gpm6a was highly expressed; Gpm6a is a four-transmembrane protein that is abundantly expressed in the nervous system [175]. The expression and function of Gpm6a in the OE have not been described previously. In the murine retina, Gpm6a is known to regulate retinal development by mediating cell-cell interactions that are involved in axon fasciculation [176]. As the visual and olfactory signal transduction systems are nearly identical on the molecular level [177], we suggest that Gpm6a and Gpm6b have similar roles during the development of ORN neurites in the OE. Of the many chaperones that are necessary for proteostasis, we detected a specific enrichment of Dnajb14 (Hsp40) in ORNs. Dnajb14 acts as a co-chaperone for the Hsp70 protein folding machinery through its role in determining substrate specificity [178]. It has previously been reported that another member of the *Hsp40* family is able to bind to, and assist in the folding of, a GPCR (progesterone receptor) [179]. Neuhaus et al. (2006) showed that the specific enrichment of Hsc70t, a variant of the Hsp70 family of heat shock proteins, in the OE, assists in the folding and trafficking of particular ORs to the plasma membrane [180]. Thus, we propose that this co-chaperone may contribute to the proteostasis of ORs or other types of GPCRs. Wdr17 is a retina-specific transcript that is thought to be involved in signaling events within and between cells [181]. Homer2, whose function in the OE has not previously been described, is highly expressed in ORNs. Homer proteins are known to promote the targeting and expression processes of GPCRs by interacting with them [182, 183]. Furthermore, it has been shown that Homer proteins are expressed in the VNO and form complexes with TRPC2 channels and IP_3_-receptors [184]. Due to the strong expression level of Homer2, we suggest that this protein is strongly involved in the chaperoning and guiding of olfactory and non-olfactory GPCRs to different membrane sites in the ORNs. Additionally, Homer2 may also be capable of interacting with and modulating, among others channels, TRP channels and thus influence the signal cascades of ORNs. In addition to Homer2, we, newly detected the expression of Slc9a3r1 and Slc9a3r2, which code for the PDZ scaffolding proteins Nherf1 and Nherf2 (Na^+^/H^+^ exchanger regulatory factor), respectively. Both candidates have been reported to interact via their PDZ domains with several GPCRs [185]. Nisar et al. (2012) demonstrated an important role for Nherf1 in potentiating GPCR internalization [186]. Here, after receptor stimulation, Nherf1 interacts with GPCRs via the scaffolding protein arrestin. Additionally, Nherf1, which is known to interact with β_2_-AR [187], regulates receptor-mediated Na^+^/H^+^-exchange and down-regulates the receptor by increasing the recycling of the receptor [188, 189]. Nherf2 is known to specifically couple LPA_2_-receptors and PLCβ3 and to regulate activity by this process [190]. Additionally, both proteins demonstrably increase the Gα_q_-mediated signaling [191, 192]. It remains to be examined whether Nherf1 and Nherf2 are also capable of regulating GPCR localization and diversifying the signal cascade of ORs in ORNs. Of the several tetraspanins that are expressed in ORNs, we found high levels of expression of Tspan7. Tetraspanins are known to regulate the signaling, trafficking and biosynthetic processing of associated proteins. Specifically, Tspan7 is involved in synaptic maturation and function and promotes the formation of filopodia and dendritic spines [193]. We assume that, due to the high levels of expression and reported functions of Tspan7, this protein is highly involved in the formation of the neurites of ORNs. Furthermore, we detected an enrichment of Ttc9 in ORNs. Ttc9 belongs to a family of tetratricopeptide repeat (TPR)-containing proteins [194] that are involved with, among other things, protein transport and folding [195, 196] and cell cycle control [197] and transcription and splicing events [198]. However, the exact function of Ttc9 in ORNs has yet to be elucidated. Next, we detected high levels of expression of two members of the Tm9sf family. Tm9sf2 and Tm9sf3 are expressed throughout the epithelium. A member of the Tm9sf family has been shown to have functional ligand properties and has consequently been suggested to function as channels or small molecule transporter or receptors [199]. Unc45a is highly and specifically expressed in ORNs, and the functions of this protein have not been well described in olfaction. It has been reported that Unc45a is involved in cytokinesis and motility via chaperoning myosin and further cooperates with Hsp90 to chaperon progesterone receptors [200, 201]. Additionally, Tusc5, an adipocyte-specific transcript, was abundant in ORNs. The reported co-expression of Tusc5 in adipocytes and peripheral somatosensory neurons [202] indicates a possible connection between the energy status of the body and distinct sensory systems. Accordingly, we suppose that Tusc5 also has a regulatory function in olfactory perception that is similar to that of Adipor1 or Paqr3 and depends on nutritional status.

In this study, we have presented several membrane proteins that have not previously been identified in OE. As the expression levels of these proteins were similar to, or even exceeded, those of the known major players in olfactory signal transduction, these proteins potentially have important roles in olfactory processes. ISH experiments confirmed the pronounced expressions of these selected genes in ORNs. Therefore, we suggest that the proteins encoded by these genes are indeed involved in the function of olfactory neurons. Future studies should concentrate on uncovering the role of these proteins in the machinery of ORNs.

Our data provide a nearly complete catalogue of the genes expressed in, and involved in, the function and maintenance of the OE, especially ORNs. The molecular portrait of the OE revealed by this quantitative and comprehensive analysis of the murine transcriptome has uncovered new and valuable approaches that will be beneficial for the advancement of knowledge regarding the molecular mechanisms underlying olfaction and the functionality of ORNs.

## Conclusion

The unmatched power of RNA-Seq in terms of quantitative and differential transcriptome analysis and the simplicity of the practical usage of this technique clearly prove that is this technique is a useful and important tool for OE transcriptomics. In this study, we were able to identify new potential players in olfaction. Furthermore, we demonstrated that these data provide a valuable framework for the interpretation and understanding of the function of ORNs. Finally, this technique currently enables the most comprehensive analyses and the easiest integration of the vast knowledge gained by previous studies.

## Supporting Information

S1 Fig Distribution of different housekeeping genes in murine tissues. Heatmap showing the expression levels of different housekeeping genes in olfactory and non-olfactory tissue. Higher FPKM values are indicated by deeper colors. Gapdh: glyceraldehyde-3-phosphate dehydrogenase, Actb: actin, cytoplasmic 1, Ldha: L-lactate dehydrogenase A chain isoform 2, Ubc: polyubiquitin-C, Tubb3: tubulin beta-3 chain, Hprt: hypoxanthine-guanine phosphoribosyltransferase.
(TIF)

S2 Fig OR detection using RNA-Seq in all replicates of the OE: Bar chart showing the percentage of detected OR genes in all replicates of OE tissue (n = 13) and FACS-sorted ORNs. Percentages were calculated based on the 1,125 OR genes annotated in the Refseq based gene model. Bars in light blue: FACS-sorted ORNs (homo- and heterozygous); blue: OE replicates of CD1 male mice (n = 4), dark blue: OE replicates of CD1 female mice (n = 5), black: OE replicates of C57BL6 female mice (n = 4).
(TIF)

S3 Fig Detection of weakly and highly expressed ORs with RNA-Seq. Sample representation of read coverage of ORs with different expression strength can be visualized by the Integrative Genomics Viewer. Shown are exemplary cufflinks data for OE of CD1 female mice; **A.** Olfr1507, FPKM = 70; **B.** Olfr730, FPKM = 5.4; **C.** Olfr1265, FPKM = 0.1. The exons are indicated by blue bars and introns by thin lines. The grey segments indicate reads that were mapped onto reference genome and red bridges exon spanning reads.. Above, the read coverage is shown (detected and mapped counts/bases at each respective position). In highly expressed ORs, 5’ UTRs can be identified by exon-spanning reads. For medium or low expressed ORs, this is not possible due to the lower number of mapped reads.
(TIF)

S4 Fig Ectopic expression of ORs in testes, brain, liver and muscle. Heatmap showing the expression of OR genes expressed in non-olfactory tissues (testes, brain, liver, muscle). Higher FPKM values are indicated by deeper colors.
(TIF)

S5 Fig Classification of FPKM values. **A: Classification of FPKM values of OR genes.** Graph showing the distribution of OR genes according to FPKM value classes in olfactory tissues (FACS sorted ORNs, OE CD1 male and female, OE C57BL/6J female. **B: Classification of FPKM values of genes.** Graph showing the distribution of genes according to FPKM value classes in olfactory tissues. Distribution confirms that the RNA-Seq data comprise the similar number of genes classified into the same range of FPKM values.
(TIF)

S6 Fig Correlation matrix of the whole data set. Chart showing the Pearson correlation coefficient values for protein-coding gene expression pattern between all replicates of the OE (upper matrix). In the lower matrix, OBP and Cpy genes were excluded from the analysis. Higher correlation between replicates is indicated by a color scale from blue to orange.
(TIF)

S7 Fig Correlations of expression levels plotted for each detected gene. Shown is the correlation of the protein-coding gene expression pattern between OE of two (exemplary chosen) individual mice for each condition (CD1 male, CD1 female and C57BL6 female). Only genes with detectable expression levels (FPKM>0.1) are shown. The FPKM values are logarithmically presented. Genes with the most diverging expression pattern belong to OBPs genes; which are marked in red.
(TIF)

S8 Fig Correlations of expression levels plotted for each detected OR gene in exemplary two biological replicates of OE from female CD1 mice. For investigation of the reproducibility of the expression pattern, two biological replicates of the transcriptome of the female OE of CD1 mice were prepared. These new datasets were based on RNA of 8 pooled OE analyzed by mRNA Illumina sequencing on a HiSeq 2000 platform which generated 54–57 million reads (101 bp, paired end). Correlation of the OR gene expression between two biological replicates of female CD1 mice is shown. A detailed analysis of these data will be given elsewhere. Only OR genes with detectable expression levels (FPKM>0.1) are shown. The FPKM values are logarithmically presented. The Pearson correlation coefficient of r = 0.9 confirmed the strong correlation of OR gene expression patterns between biological replicates.
(TIF)

S9 Fig Correlation matrix of the OR subgenome. Chart showing the Pearson correlation coefficient values for OR gene expression between all replicates of the OE (n = 13). Higher correlation between replicates is indicated by a color scale from blue to orange.
(TIF)

S10 Fig Top expressed OR genes. Chart showing the expression ranking of the top 20 OR genes in OE replicates (CD1 male, CD1 female and C57BL6 mice) and ORNs. The OR genes Olfr1507, Olfr533 and Olfr309 are highly expressed. These receptors can be detected among the 20 most highly expressed OR genes in each replicate of the OE and ORNs.
(TIF)

S11 Fig Distribution of FPKM values of detected nGPCRs. Bar chart showing the distribution of FPKM classes in ORNs, OE of CD1 mice (both sexes) and OE of female C57BL/6J.
(TIF)

S12 Fig *In situ* hybridization for mRNA of catalytic PI3K subunits. Expression of transcripts for p110α (1), p110β (2), p110γ (3) and p110δ (4) were detected in the mature ORN cell layer as predicted by the expression in sorted ORNs RNA-Seq data. Sense (a) and antisense (b) RNA probes were tested in parallel and show the antisense specific staining
(TIF)

S13 Fig Expression level of receptor genes regulating food intake. Bar chart showing the expression level of receptor genes regulating food intake in the OE. Adipor1 is by far the most highly expressed gene. Receptors for insulin, leptin or ghrelin, are weakly expressed. FPKM values are presented exemplary from CD1 male OE. Adipor1: adiponectin receptor1, Insr: insulin receptor, Paqr3: progestin and adipoQ receptor family member III, Ghrl: ghrelin receptor, Lepr: leptin receptor, Npy1r: neuropeptide Y receptor, Glp1r/Glp2r: Glucagon-like peptide receptor, Hctr1/Hctr2: orexin receptors, Mc3r/Mc4r: melanocortin receptors.
(TIF)

S1 Table RNASeq data OE (CD1 male, CD1 and C57BL/6J female) and FACS sorted ORNs. (XLSX)

S2 Table Cuffdiff analysis for differential gene expression between homozygous and heterozygous ORNs datasets. (XLSX)

S3 Table RNASeq data reference tissue: brain, liver, muscle and testis. (XLSX)

S4 Table Raw data sets of RNASeq used in this study. (DOCX)

S5 Table OR genes. (XLSX)

S6 Table OR pseudo-genes. (XLSX)

S7 Table Non-olfactory GPCRs. (XLSX)

S8 Table Non-GPCR membrane proteins. (XLSX)

S9 Table Transporter: channels, SLCs and active transporters. (XLSX)

S10 Table PI3 kinases. (XLSX)

S11 Table Enriched genes in ORNs. (XLSX)

## References

[1] Godfrey PA, Malnic B, Buck LB (2004) The mouse olfactory receptor gene family. Proc. Natl. Acad. Sci. U.S.A. 101 (7): 2156–2161. 10.1073/pnas.0308051100 14769939 PMC357068

[2] Mombaerts P (2004) Odorant receptor gene choice in olfactory sensory neurons: the one receptor-one neuron hypothesis revisited. Curr. Opin. Neurobiol. 14 (1): 31–36. 10.1016/j.conb.2004.01.014 15018935

[3] Buck L, Axel R (1991) A novel multigene family may encode odorant receptors: a molecular basis for odor recognition. Cell 65 (1): 175–187. 10.1016/0092-8674(91)90418-X 1840504

[4] Glusman G, Yanai I, Rubin I, Lancet D (2001) The complete human olfactory subgenome. Genome Res. 11 (5): 685–702. 10.1101/gr.171001 11337468

[5] Niimura Y, Nei M (2007) Extensive gains and losses of olfactory receptor genes in mammalian evolution. PLoS ONE 2 (8): e708 10.1371/journal.pone.0000708 17684554 PMC1933591

[6] DeMaria S, Ngai J (2010) The cell biology of smell. J. Cell Biol. 191 (3): 443–452. 10.1083/jcb.201008163 21041441 PMC3003330

[7] Malnic B, Hirono J, Sato T, Buck LB (1999) Combinatorial receptor codes for odors. Cell 96 (5): 713–723. 10.1016/S0092-8674(00)80581-4 10089886

[8] Jones DT, Reed R (1989) Golf: an olfactory neuron specific-G protein involved in odorant signal transduction. Science 244 (4906): 790–795. 10.1126/science.2499043 2499043

[9] Bakalyar HA, Reed R (1990) Identification of a specialized adenylyl cyclase that may mediate odorant detection. Science 250 (4986): 1403–1406. 10.1126/science.2255909 2255909

[10] Nakamura T, Gold GH (1987) A cyclic nucleotide-gated conductance in olfactory receptor cilia. Nature 325 (6103): 442–444. 10.1038/325442a0 3027574

[11] Michalakis S, Reisert J, Geiger H, Wetzel C, Zong X, et al (2006) Loss of CNGB1 protein leads to olfactory dysfunction and subciliary cyclic nucleotide-gated channel trapping. J. Biol. Chem. 281 (46): 35156–35166. 10.1074/jbc.M606409200 16980309 PMC2885922

[12] Pifferi S, Boccaccio A, Menini A (2006) Cyclic nucleotide-gated ion channels in sensory transduction. FEBS Lett. 580 (12): 2853–2859. 10.1016/j.febslet.2006.03.086 16631748

[13] Kleene SJ, Gesteland R (1991) Calcium-activated chloride conductance in frog olfactory cilia. J. Neurosci. 11 (11): 3624–3629. 1941099 10.1523/JNEUROSCI.11-11-03624.1991 PMC6575529

[14] Reisert J, Bauer PJ, Yau K, Frings S (2003) The Ca-activated Cl channel and its control in rat olfactory receptor neurons. J. Gen. Physiol. 122 (3): 349–363. 10.1085/jgp.200308888 12939394 PMC2234486

[15] Stephan AB, Shum EY, Hirsh S, Cygnar KD, Reisert J, et al (2009) ANO2 is the cilial calcium-activated chloride channel that may mediate olfactory amplification. Proc. Natl. Acad. Sci. U.S.A. 106 (28): 11776–11781. 10.1073/pnas.0903304106 19561302 PMC2702256

[16] Rasche S, Toetter B, Adler J, Tschapek A, Doerner JF, et al (2010) Tmem16b is specifically expressed in the cilia of olfactory sensory neurons. Chem. Senses 35 (3): 239–245. 10.1093/chemse/bjq007 20100788

[17] Hengl T, Kaneko H, Dauner K, Vocke K, Frings S, et al (2010) Molecular components of signal amplification in olfactory sensory cilia. Proc. Natl. Acad. Sci. U.S.A. 107 (13): 6052–6057. 10.1073/pnas.0909032107 20231443 PMC2851919

[18] Kaneko H, Putzier I, Frings S, Kaupp UB, Gensch T (2004) Chloride accumulation in mammalian olfactory sensory neurons. J. Neurosci. 24 (36): 7931–7938. 10.1523/JNEUROSCI.2115-04.2004 15356206 PMC6729923

[19] Reisert J, Lai J, Yau K, Bradley J (2005) Mechanism of the excitatory Cl- response in mouse olfactory receptor neurons. Neuron 45 (4): 553–561. 10.1016/j.neuron.2005.01.012 15721241 PMC2877386

[20] Nickell WT, Kleene NK, Kleene SJ (2007) Mechanisms of neuronal chloride accumulation in intact mouse olfactory epithelium. J. Physiol. (Lond.) 583 (Pt 3): 1005–1020. 10.1113/jphysiol.2007.129601 17656441 PMC2277205

[21] Saito H, Kubota M, Roberts RW, Chi Q, Matsunami H (2004) RTP family members induce functional expression of mammalian odorant receptors. Cell 119 (5): 679–691. 15550249 10.1016/j.cell.2004.11.021

[22] Boekhoff I, Breer (1992) Termination of second messenger signaling in olfaction. Proc. Natl. Acad. Sci. U.S.A. 89 (2): 471–474. 10.1073/pnas.89.2.471 1370581 PMC48260

[23] Boekhoff I, Inglese J, Schleicher S, Koch WJ, Lefkowitz RJ, et al (1994) Olfactory desensitization requires membrane targeting of receptor kinase mediated by beta gamma-subunits of heterotrimeric G proteins. J. Biol. Chem. 269 (1): 37–40. 8276821

[24] Schleicher S, Boekhoff I, Arriza J, Lefkowitz RJ, Breer H (1993) A beta-adrenergic receptor kinase-like enzyme is involved in olfactory signal termination. Proc. Natl. Acad. Sci. U.S.A. 90 (4): 1420–1424. 10.1073/pnas.90.4.1420 8381966 PMC45885

[25] Dawson TM, Arriza JL, Jaworsky DE, Borisy FF, Attramadal H, et al (1993) Beta-adrenergic receptor kinase-2 and beta-arrestin-2 as mediators of odorant-induced desensitization. Science 259 (5096): 825–829. 10.1126/science.8381559 8381559

[26] Peppel K, Boekhoff I, McDonald P, Breer H, Caron MG, et al (1997) G protein-coupled receptor kinase 3 (GRK3) gene disruption leads to loss of odorant receptor desensitization. J. Biol. Chem. 272 (41): 25425–25428. 10.1074/jbc.272.41.25425 9325250

[27] Mashukova A, Spehr M, Hatt H, Neuhaus EM (2006) Beta-arrestin2-mediated internalization of mammalian odorant receptors. J. Neurosci. 26 (39): 9902–9912. 10.1523/JNEUROSCI.2897-06.2006 17005854 PMC6674477

[28] Dooley R, Baumgart S, Rasche S, Hatt H, Neuhaus EM (2009) Olfactory receptor signaling is regulated by the post-synaptic density 95, Drosophila discs large, zona-occludens 1 (PDZ) scaffold multi-PDZ domain protein 1. FEBS J. 276 (24): 7279–7290. 10.1111/j.1742-4658.2009.07435.x 19909339

[29] Dannecker LEC von, Mercadante AF, Malnic B (2005) Ric-8B, an olfactory putative GTP exchange factor, amplifies signal transduction through the olfactory-specific G-protein Galphaolf. J. Neurosci. 25 (15): 3793–3800. 10.1523/JNEUROSCI.4595-04.2005 15829631 PMC6724935

[30] Dannecker LEC von, Mercadante AF, Malnic B (2006) Ric-8B promotes functional expression of odorant receptors. Proc. Natl. Acad. Sci. U.S.A. 103 (24): 9310–9314. 10.1073/pnas.0600697103 16754875 PMC1482606

[31] Kerr DS, Dannecker LEC von, Davalos M, Michaloski JS, Malnic B (2008) Ric-8B interacts with G alpha olf and G gamma 13 and co-localizes with G alpha olf, G beta 1 and G gamma 13 in the cilia of olfactory sensory neurons. Mol. Cell. Neurosci. 38 (3): 341–348. 10.1016/j.mcn.2008.03.006 18462949

[32] Wei J, Wayman G, Storm D (1996) Phosphorylation and inhibition of type III adenylyl cyclase by calmodulin-dependent protein kinase II in vivo. J. Biol. Chem. 271 (39): 24231–24235. 10.1074/jbc.271.39.24231 8798667

[33] Yan C, Zhao AZ, Bentley JK, Loughney K, Ferguson K, et al (1995) Molecular cloning and characterization of a calmodulin-dependent phosphodiesterase enriched in olfactory sensory neurons. Proc. Natl. Acad. Sci. U.S.A. 92 (21): 9677–9681. 10.1073/pnas.92.21.9677 7568196 PMC40865

[34] Yan C, Zhao AZ, Bentley JK, Beavo J (1996) The calmodulin-dependent phosphodiesterase gene PDE1C encodes several functionally different splice variants in a tissue-specific manner. J. Biol. Chem. 271 (41): 25699–25706. 10.1074/jbc.271.41.25699 8810348

[35] Cherry JA, Davis RL (1995) A mouse homolog of dunce, a gene important for learning and memory in Drosophila, is preferentially expressed in olfactory receptor neurons. J. Neurobiol. 28 (1): 102–113. 10.1002/neu.480280109 8586960

[36] Davis RL, Cherry J, Dauwalder B, Han PL, Skoulakis E (1995) The cyclic AMP system and Drosophila learning. Mol. Cell. Biochem. 149–150: 271–278. 10.1007/BF01076588 8569740

[37] Pho V, Butman ML, Cherry JA (2005) Type 4 phosphodiesterase inhibition impairs detection of low odor concentrations in mice. Behav. Brain Res. 161 (2): 245–253. 10.1016/j.bbr.2005.02.011 15922051

[38] Zufall F, Leinders-Zufall T (2000) The cellular and molecular basis of odor adaptation. Chem. Senses 25 (4): 473–481. 10.1093/chemse/25.4.473 10944513

[39] Munger SD, Gleeson RA, Aldrich HC, Rust NC, Ache BW, et al (2000) Characterization of a phosphoinositide-mediated odor transduction pathway reveals plasma membrane localization of an inositol 1,4, 5-trisphosphate receptor in lobster olfactory receptor neurons. J. Biol. Chem. 275 (27): 20450–20457. 10.1074/jbc.M001989200 10781594

[40] Munger SD, Lane AP, Zhong H, Leinders-Zufall T, Yau KW, et al (2001) Central role of the CNGA4 channel subunit in Ca2+-calmodulin-dependent odor adaptation. Science 294 (5549): 2172–2175. 10.1126/science.1063224 11739959 PMC2885906

[41] Kelliher KR, Ziesmann J, Munger SD, Reed RR, Zufall F (2003) Importance of the CNGA4 channel gene for odor discrimination and adaptation in behaving mice. Proc. Natl. Acad. Sci. U.S.A. 100 (7): 4299–4304. 10.1073/pnas.0736071100 12649326 PMC153087

[42] Stephan AB, Tobochnik S, Dibattista M, Wall CM, Reisert J, et al (2012) The Na(+)/Ca(2+) exchanger NCKX4 governs termination and adaptation of the mammalian olfactory response. Nat. Neurosci. 15 (1): 131–137. 10.1038/nn.2943 PMC3245797 22057188

[43] Klasen K, Corey EA, Kuck F, Wetzel CH, Hatt H, et al (2010) Odorant-stimulated phosphoinositide signaling in mammalian olfactory receptor neurons. Cell. Signal. 22 (1): 150–157. 10.1016/j.cellsig.2009.09.026 19781634 PMC3581345

[44] Zhainazarov AB, Doolin R, Herlihy JD, Ache (2001) Odor-stimulated phosphatidylinositol 3-kinase in lobster olfactory receptor cells. J. Neurophysiol. 85 (6): 2537–2544. 11387399 10.1152/jn.2001.85.6.2537

[45] Spehr M, Wetzel CH, Hatt H, Ache B (2002) 3-phosphoinositides modulate cyclic nucleotide signaling in olfactory receptor neurons. Neuron 33 (5): 731–739. 10.1016/S0896-6273(02)00610-4 11879650

[46] Ukhanov K, Corey EA, Brunert D, Klasen K, Ache BW (2010) Inhibitory odorant signaling in Mammalian olfactory receptor neurons. J. Neurophysiol. 103 (2): 1114–1122. 10.1152/jn.00980.2009 20032232 PMC2822701

[47] Ukhanov K, Brunert D, Corey EA, Ache BW (2011) Phosphoinositide 3-kinase-dependent antagonism in mammalian olfactory receptor neurons. J. Neurosci. 31 (1): 273–280. 10.1523/JNEUROSCI.3698-10.2011 21209212 PMC3079265

[48] Sammeta N, Yu T, Bose SC, McClintock TS (2007) Mouse olfactory sensory neurons express 10,000 genes. J. Comp. Neurol. 502 (6): 1138–1156. 10.1002/cne.21365 17444493

[49] Nickell MD, Breheny P, Stromberg AJ, McClintock TS (2012) Genomics of mature and immature olfactory sensory neurons. J. Comp. Neurol. 520 (12): 2608–2629. 10.1002/cne.23052 22252456 PMC4023872

[50] Potter SM, Zheng C, Koos DS, Feinstein P, Fraser SE et al (2001) Structure and emergence of specific olfactory glomeruli in the mouse. J. Neurosci. 21 (24): 9713–9723. 11739580 10.1523/JNEUROSCI.21-24-09713.2001 PMC2570017

[51] Shiao M, Chang AY, Liao B, Ching Y, Lu MJ, et al (2012) Transcriptomes of mouse olfactory epithelium reveal sexual differences in odorant detection. Genome Biol Evol 4 (5): 703–712. 10.1093/gbe/evs039 22511034 PMC3381674

[52] Keydar I, Ben-Asher E, Feldmesser E, Nativ N, Oshimoto A, et al (2013) General olfactory sensitivity database (GOSdb): candidate genes and their genomic variations. Hum. Mutat. 34 (1): 32–41. 10.1002/humu.22212 22936402 PMC3627721

[53] Mayer U, Ungerer N, Klimmeck D, Warnken U, Schnölzer M, et al (2008) Proteomic analysis of a membrane preparation from rat olfactory sensory cilia. Chem. Senses 33 (2): 145–162. 10.1093/chemse/bjm073 18032372

[54] Barbour J, Neuhaus EM, Piechura H, Stoepel N, Mashukova A, et al (2008) New insight into stimulus-induced plasticity of the olfactory epithelium in Mus musculus by quantitative proteomics. J. Proteome Res. 7 (4): 1594–1605. 10.1021/pr7005796 18336002

[55] Mayer U, Küller A, Daiber PC, Neudorf I, Warnken U, et al (2009) The proteome of rat olfactory sensory cilia. Proteomics 9 (2): 322–334. 10.1002/pmic.200800149 19086097

[56] Klimmeck D, Mayer U, Ungerer N, Warnken U, Schnölzer M, et al (2008) Calcium-signaling networks in olfactory receptor neurons. Neuroscience 151 (3): 901–912. 10.1016/j.neuroscience.2007.11.023 18155848

[57] Trapnell C, Roberts A, Goff L, Pertea G, Kim D, et al (2012) Differential gene and transcript expression analysis of RNA-seq experiments with TopHat and Cufflinks. Nat Protoc 7 (3): 562–578. 10.1038/nprot.2012.016 22383036 PMC3334321

[58] Trapnell C, Pachter L, Salzberg SL (2009) TopHat: discovering splice junctions with RNA-Seq. Bioinformatics 25 (9): 1105–1111. 10.1093/bioinformatics/btp120 19289445 PMC2672628

[59] Langmead B, Trapnell C, Pop M, Salzberg SL (2009) Ultrafast and memory-efficient alignment of short DNA sequences to the human genome. Genome Biol. 10 (3): R25 10.1186/gb-2009-10-3-r25 19261174 PMC2690996

[60] Li H, Handsaker B, Wysoker A, Fennell T, Ruan J, et al (2009) The Sequence Alignment/Map format and SAMtools. Bioinformatics 25 (16): 2078–2079. 10.1093/bioinformatics/btp352 19505943 PMC2723002

[61] Trapnell C, Williams BA, Pertea G, Mortazavi A, Kwan G, et al (2010) Transcript assembly and quantification by RNA-Seq reveals unannotated transcripts and isoform switching during cell differentiation. Nat. Biotechnol. 28 (5): 511–515. 10.1038/nbt.1621 20436464 PMC3146043

[62] Roberts A, Trapnell C, Donaghey J, Rinn JL, Pachter L (2011) Improving RNA-Seq expression estimates by correcting for fragment bias. Genome Biol. 12 (3): R22 10.1186/gb-2011-12-3-r22 21410973 PMC3129672

[63] Mortazavi A, Williams BA, McCue K, Schaeffer L, Wold B (2008) Mapping and quantifying mammalian transcriptomes by RNA-Seq. Nat. Methods 5 (7): 621–628. 10.1038/nmeth.1226 18516045 PMC13303166

[64] Harr B, Turner LM (2010) Genome-wide analysis of alternative splicing evolution among Mus subspecies. Mol. Ecol. 19 Suppl 1: 228–239. 10.1111/j.1365-294X.2009.04490.x 20331782

[65] Dubacq C, Jamet S, Trembleau A (2009) Evidence for developmentally regulated local translation of odorant receptor mRNAs in the axons of olfactory sensory neurons. J. Neurosci. 29 (33): 10184–10190. 10.1523/JNEUROSCI.2443-09.2009 19692593 PMC6665787

[66] Yu T, McIntyre JC, Bose SC, Hardin D, Owen MC, et al (2005) Differentially expressed transcripts from phenotypically identified olfactory sensory neurons. J. Comp. Neurol. 483 (3): 251–262. 10.1002/cne.20429 15682396 PMC2967457

[67] Lai PC, Bahl G, Gremigni M, Matarazzo V, Clot-Faybesse O, et al (2008) An olfactory receptor pseudogene whose function emerged in humans: a case study in the evolution of structure-function in GPCRs. J. Struct. Funct. Genomics 9 (1–4): 29–40. 10.1007/s10969-008-9043-x 18802787 PMC3197733

[68] Kajiya K, Inaki K, Tanaka M, Haga T, Kataoka H, et al (2001) Molecular bases of odor discrimination: Reconstitution of olfactory receptors that recognize overlapping sets of odorants. J. Neurosci. 21 (16): 6018–6025. 11487625 10.1523/JNEUROSCI.21-16-06018.2001 PMC6763140

[69] Pes D, Pelosi P (1995) Odorant-binding proteins of the mouse. Comp Biochem Physiol B Biochem Mol Biol 112 (3): 471–479. 10.1016/0305-0491(95)00063-1 8529023

[70] Ling G, Gu J, Genter MB, Zhuo X, Ding X (2004) Regulation of cytochrome P450 gene expression in the olfactory mucosa. Chem Biol Interact 147 (3): 247–258. 10.1016/j.cbi.2004.02.003 15135081

[71] Liberles SD, Buck LB (2006) A second class of chemosensory receptors in the olfactory epithelium. Nature 442 (7103): 645–650. 10.1038/nature05066 16878137

[72] Liberles SD, Horowitz LF, Kuang D, Contos JJ, Wilson KL, et al (2009) Formyl peptide receptors are candidate chemosensory receptors in the vomeronasal organ. Proc. Natl. Acad. Sci. U.S.A. 106 (24): 9842–9847. 10.1073/pnas.0904464106 19497865 PMC2690606

[73] Rivière S, Challet L, Fluegge D, Spehr M, Rodriguez I (2009) Formyl peptide receptor-like proteins are a novel family of vomeronasal chemosensors. Nature 459 (7246): 574–577. 10.1038/nature08029 19387439

[74] Fülle HJ, Vassar R, Foster DC, Yang RB, Axel R, et al (1995) A receptor guanylyl cyclase expressed specifically in olfactory sensory neurons. Proc. Natl. Acad. Sci. U.S.A. 92 (8): 3571–3575. 10.1073/pnas.92.8.3571 7724600 PMC42209

[75] Chaudhari N, Pereira E, Roper SD (2009) Taste receptors for umami: the case for multiple receptors. Am. J. Clin. Nutr. 90 (3): 738S–742S. 10.3945/ajcn.2009.27462H 19571230 PMC3136002

[76] Zheng J, Zagotta WN (2004) Stoichiometry and assembly of olfactory cyclic nucleotide-gated channels. Neuron 42 (3): 411–421. 10.1016/S0896-6273(04)00253-3 15134638

[77] Stephan AB, Shum EY, Hirsh S, Cygnar KD, Reisert J, et al (2009) ANO2 is the cilial calcium-activated chloride channel that may mediate olfactory amplification. Proc. Natl. Acad. Sci. U.S.A. 106 (28): 11776–11781. 10.1073/pnas.0903304106 19561302 PMC2702256

[78] Neuhaus EM, Mashukova A, Barbour J, Wolters D, Hatt H (2006) Novel function of beta-arrestin2 in the nucleus of mature spermatozoa. J. Cell. Sci. 119 (Pt 15): 3047–3056. 10.1242/jcs.03046 16820410

[79] Anholt RR, Mumby SM, Stoffers DA, Girard PR, Kuo JF, et al (1987) Transduction proteins of olfactory receptor cells: identification of guanine nucleotide binding proteins and protein kinase C. Biochemistry 26 (3): 788–795. 10.1021/bi00377a020 3105575

[80] Margolis FL, Verhaagen J, Biffo S, Huang FL, Grillo M (1991) Regulation of gene expression in the olfactory neuroepithelium: a neurogenetic matrix. Prog. Brain Res. 89: 97–122. 10.1016/S0079-6123(08)61718-5 1839074

[81] Bruch R (1996) Phosphoinositide second messengers in olfaction. Comp. Biochem. Physiol. B, Biochem. Mol. Biol. 113 (3): 451–459. 10.1016/0305-0491(95)02040-3 8829799

[82] Weiss J, Pyrski M, Jacobi E, Bufe B, Willnecker V, et al (2011) Loss-of-function mutations in sodium channel Nav1.7 cause anosmia. Nature 472 (7342): 186–190. 10.1038/nature09975 21441906 PMC3674497

[83] Goldstein BJ, Kulaga HM, Reed RR (2003) Cloning and characterization of SLP3: a novel member of the stomatin family expressed by olfactory receptor neurons. J. Assoc. Res. Otolaryngol. 4 (1): 74–82. 10.1007/s10162-002-2039-5 12239636 PMC3202447

[84] Graziadei GA, Graziadei P (1979) Neurogenesis and neuron regeneration in the olfactory system of mammals. II. Degeneration and reconstitution of the olfactory sensory neurons after axotomy. J. Neurocytol. 8 (2): 197–213. 10.1007/BF01175561 469573

[85] Huard JM, Youngentob SL, Goldstein BJ, Luskin MB, Schwob JE (1998) Adult olfactory epithelium contains multipotent progenitors that give rise to neurons and non-neural cells. J. Comp. Neurol. 400 (4): 469–486. 10.1002/(SICI)1096-9861(19981102)400:4<469::AID-CNE3>3.3.CO;2-L 9786409

[86] Li YR, Matsunami H (2011) Activation state of the M3 muscarinic acetylcholine receptor modulates mammalian odorant receptor signaling. Sci Signal 4 (155): ra1 10.1126/scisignal.2001230 21224444 PMC3034603

[87] Hague C, Hall RA, Minneman KP (2004) Olfactory receptor localization and function: an emerging role for GPCR heterodimerization. Mol. Interv. 4 (6): 321–322. 10.1124/mi.4.6.4 15616160

[88] Bauer S, Grossmann S, Vingron M, Robinson PN (2008) Ontologizer 2.0—a multifunctional tool for GO term enrichment analysis and data exploration. Bioinformatics 24 (14): 1650–1651. 10.1093/bioinformatics/btn250 18511468

[89] Lin W, Margolskee R, Donnert G, Hell SW, Restrepo D (2007) Olfactory neurons expressing transient receptor potential channel M5 (TRPM5) are involved in sensing semiochemicals. Proc. Natl. Acad. Sci. U.S.A. 104 (7): 2471–2476. 10.1073/pnas.0610201104 17267604 PMC1892929

[90] Schöbel N, Radtke D, Lübbert M, Gisselmann G, Lehmann R, et al (2012) Trigeminal ganglion neurons of mice show intracellular chloride accumulation and chloride-dependent amplification of capsaicin-induced responses. PLoS ONE 7 (11): e48005 10.1371/journal.pone.0048005 23144843 PMC3493563

[91] Hahn Y, Kim DS, Pastan IH, Lee B (2009) Anoctamin and transmembrane channel-like proteins are evolutionarily related. Int. J. Mol. Med. 24 (1): 51–55. 10.3892/ijmm_00000205 19513534 PMC2695565

[92] Fried H, Kaupp UB, Müller F (2010) Hyperpolarization-activated and cyclic nucleotide-gated channels are differentially expressed in juxtaglomerular cells in the olfactory bulb of mice. Cell Tissue Res. 339 (3): 463–479. 10.1007/s00441-009-0904-9 20140458 PMC2838509

[93] Khananshvili D (2013) The SLC8 gene family of sodium-calcium exchangers (NCX)—structure, function, and regulation in health and disease. Mol. Aspects Med. 34 (2–3): 220–235. 10.1016/j.mam.2012.07.003 23506867

[94] Menini A (1999) Calcium signalling and regulation in olfactory neurons. Curr. Opin. Neurobiol. 9 (4): 419–426. 10.1016/S0959-4388(99)80063-4 10448159

[95] Sammeta N, Hardin DL, McClintock TS (2010) Uncx regulates proliferation of neural progenitor cells and neuronal survival in the olfactory epithelium. Mol. Cell. Neurosci. 45 (4): 398–407. 10.1016/j.mcn.2010.07.013 20692344 PMC2962766

[96] Firestein S, Shepherd GM, Werblin FS (1990) Time course of the membrane current underlying sensory transduction in salamander olfactory receptor neurones. J. Physiol. (Lond.) 430: 135–158. 10.1113/jphysiol.1990.sp018286 2086763 PMC1181732

[97] Cygnar KD, Zhao H (2009) Phosphodiesterase 1C is dispensable for rapid response termination of olfactory sensory neurons. Nat. Neurosci. 12 (4): 454–462. 10.1038/nn.2289 19305400 PMC2712288

[98] Juilfs DM, Fülle HJ, Zhao AZ, Houslay MD, Garbers DL, et al (1997) A subset of olfactory neurons that selectively express cGMP-stimulated phosphodiesterase (PDE2) and guanylyl cyclase-D define a unique olfactory signal transduction pathway. Proc. Natl. Acad. Sci. U.S.A. 94 (7): 3388–3395. 10.1073/pnas.94.7.3388 9096404 PMC20380

[99] Cote RH (2004) Characteristics of photoreceptor PDE (PDE6): similarities and differences to PDE5. Int. J. Impot. Res. 16 Suppl 1: S28–33. 10.1038/sj.ijir.3901212 15224133

[100] Cote RH (2005) Cyclic guanosine 5’-monophosphate binding to regulatory GAF domains of photoreceptor phosphodiesterase. Methods Mol. Biol. 307: 141–154. 15988061 10.1385/1-59259-839-0:141

[101] Taylor SS, Buechler JA, Yonemoto W (1990) cAMP-dependent protein kinase: framework for a diverse family of regulatory enzymes. Annu. Rev. Biochem. 59: 971–1005. 10.1146/annurev.bi.59.070190.004543 2165385

[102] Ache BW (2010) Odorant-specific modes of signaling in mammalian olfaction. Chem. Senses 35 (7): 533–539. 10.1093/chemse/bjq045 20519266 PMC2924424

[103] Brunert D, Klasen K, Corey EA, Ache BW (2010) PI3Kgamma-dependent signaling in mouse olfactory receptor neurons. Chem. Senses 35 (4): 301–308. 10.1093/chemse/bjq020 20190008 PMC2854420

[104] Restrepo D, Miyamoto T, Bryant BP, Teeter JH (1990) Odor stimuli trigger influx of calcium into olfactory neurons of the channel catfish. Science 249 (4973): 1166–1168. 10.1126/science.2168580 2168580

[105] Fadool DA, Ache BW (1992) Plasma membrane inositol 1,4,5-trisphosphate-activated channels mediate signal transduction in lobster olfactory receptor neurons. Neuron 9 (5): 907–918. 10.1016/0896-6273(92)90243-7 1384577 PMC2843424

[106] Kalinoski DL, Aldinger SB, Boyle AG, Huque T, Marecek JF, et al (1992) Characterization of a novel inositol 1,4,5-trisphosphate receptor in isolated olfactory cilia. Biochem. J. 281 (Pt 2): 449–456. 1310597 10.1042/bj2810449 PMC1130706

[107] Restrepo D, Teeter JH, Honda E, Boyle AG, Marecek JF, et al (1992) Evidence for an InsP3-gated channel protein in isolated rat olfactory cilia. Am. J. Physiol. 263 (3 Pt 1): C667–73 1384346 10.1152/ajpcell.1992.263.3.C667

[108] Cunningham AM, Ryugo DK, Sharp AH, Reed RR, Snyder SH et al (1993) Neuronal inositol 1,4,5-trisphosphate receptor localized to the plasma membrane of olfactory cilia. Neuroscience 57 (2): 339–352. 10.1016/0306-4522(93)90067-P 8115043

[109] Clapham DE (2003) TRP channels as cellular sensors. Nature 426 (6966): 517–524. 10.1038/nature02196 14654832

[110] Elsaesser R, Montani G, Tirindelli R, Paysan J (2005) Phosphatidyl-inositide signalling proteins in a novel class of sensory cells in the mammalian olfactory epithelium. Eur. J. Neurosci. 21 (10): 2692–2700. 10.1111/j.1460-9568.2005.04108.x 15926917

[111] Runnels LW, Yue L, Clapham DE (2002) The TRPM7 channel is inactivated by PIP(2) hydrolysis. Nat. Cell Biol. 4 (5): 329–336. 11941371 10.1038/ncb781

[112] Pluznick JL, Rodriguez-Gil DJ, Hull M, Mistry K, Gattone V, et al (2011) Renal cystic disease proteins play critical roles in the organization of the olfactory epithelium. PLoS ONE 6 (5): e19694 10.1371/journal.pone.0019694 21614130 PMC3094399

[113] McClintock TS, Glasser CE, Bose SC, Bergman DA (2008) Tissue expression patterns identify mouse cilia genes. Physiol. Genomics 32 (2): 198–206. 10.1152/physiolgenomics.00128.2007 17971504

[114] Kream RM, Margolis FL (1984) Olfactory marker protein: turnover and transport in normal and regenerating neurons. J Neurosci 4 (3): 868–879. 6707736 10.1523/JNEUROSCI.04-03-00868.1984 PMC6564834

[115] Khan M, Vaes E, Mombaerts P (2011) Regulation of the probability of mouse odorant receptor gene choice. Cell 147 (4): 907–921. 10.1016/j.cell.2011.09.049 22078886

[116] Serizawa S, Miyamichi K, Nakatani H, Suzuki M, Saito M, et al (2003) Negative feedback regulation ensures the one receptor-one olfactory neuron rule in mouse. Science 302 (5653): 2088–2094. 10.1126/science.1089122 14593185

[117] Wakabayashi Y, Mori Y, Ichikawa M, Yazaki K, Hagino-Yamagishi K (2002) A putative pheromone receptor gene is expressed in two distinct olfactory organs in goats. Chem. Senses 27 (3): 207–213. 10.1093/chemse/27.3.207 11923183

[118] Xu F, Schaefer M, Kida I, Schafer J, Liu N, et al (2005) Simultaneous activation of mouse main and accessory olfactory bulbs by odors or pheromones. J. Comp. Neurol. 489 (4): 491–500. 10.1002/cne.20652 16025460

[119] Zufall F (2005) The TRPC2 ion channel and pheromone sensing in the accessory olfactory system. Naunyn Schmiedebergs Arch. Pharmacol. 371 (4): 245–250. 10.1007/s00210-005-1028-8 15871013

[120] Zufall F, Kelliher KR, Leinders-Zufall T (2002) Pheromone detection by mammalian vomeronasal neurons. Microsc. Res. Tech. 58 (3): 251–260. 10.1002/jemt.10152 12203702

[121] Takami S, Yukimatsu M, Matsumura G, Nishiyama F (2001) Vomeronasal epithelial cells of human fetuses contain immunoreactivity for G proteins, Go(alpha) and Gi(alpha 2). Chem. Senses 26 (5): 517–522. 10.1093/chemse/26.5.517 11418497

[122] Ohmoto M, Matsumoto I, Yasuoka A, Yoshihara Y, Abe K (2008) Genetic tracing of the gustatory and trigeminal neural pathways originating from T1R3-expressing taste receptor cells and solitary chemoreceptor cells. Mol. Cell. Neurosci. 38 (4): 505–517. 10.1016/j.mcn.2008.04.011 18539481

[123] Nancy V, Callebaut I, El Marjou A, Gunzburg J de (2002) The delta subunit of retinal rod cGMP phosphodiesterase regulates the membrane association of Ras and Rap GTPases. J. Biol. Chem. 277 (17): 15076–15084. 11786539 10.1074/jbc.M109983200

[124] Nikolova S, Guenther A, Savai R, Weissmann N, Ghofrani HA, et al (2010) Phosphodiesterase 6 subunits are expressed and altered in idiopathic pulmonary fibrosis. Respir. Res. 11: 146 10.1186/1465-9921-11-146 20979602 PMC2988012

[125] Nilius B, Owsianik G, Voets T (2008) Transient receptor potential channels meet phosphoinositides. EMBO J. 27 (21): 2809–2816. 10.1038/emboj.2008.217 18923420 PMC2570475

[126] Liu D, Liman ER (2003) Intracellular Ca2+ and the phospholipid PIP2 regulate the taste transduction ion channel TRPM5. Proc. Natl. Acad. Sci. U.S.A. 100 (25): 15160–15165. 10.1073/pnas.2334159100 14657398 PMC299934

[127] Storch U, Forst A, Philipp M, Gudermann T, Mederos y Schnitzler M (2012) Transient receptor potential channel 1 (TRPC1) reduces calcium permeability in heteromeric channel complexes. J. Biol. Chem. 287 (5): 3530–3540. 10.1074/jbc.M111.283218 22157757 PMC3271006

[128] Kerstein PC, Jacques-Fricke BT, Rengifo J, Mogen BJ, Williams JC, et al (2013) Mechanosensitive TRPC1 channels promote calpain proteolysis of talin to regulate spinal axon outgrowth. J. Neurosci. 33 (1): 273–285. 10.1523/JNEUROSCI.2142-12.2013 23283340 PMC3539200

[129] Madsen CP, Klausen TK, Fabian A, Hansen BJ, Pedersen SF, et al (2012) On the role of TRPC1 in control of Ca2+ influx, cell volume, and cell cycle. Am. J. Physiol., Cell Physiol. 303 (6): C625–34. 10.1152/ajpcell.00287.2011 22744003

[130] Bates-Withers C, Sah R, Clapham DE (2011) TRPM7, the Mg(2+) inhibited channel and kinase. Adv. Exp. Med. Biol. 704: 173–183. 10.1007/978-94-007-0265-3_9 21290295

[131] Jin J, Desai BN, Navarro B, Donovan A, Andrews NC et al (2008) Deletion of Trpm7 disrupts embryonic development and thymopoiesis without altering Mg2+ homeostasis. Science 322 (5902): 756–760. 10.1126/science.1163493 18974357 PMC2605283

[132] Jin J, Wu L, Jun J, Cheng X, Xu H, et al (2012) The channel kinase, TRPM7, is required for early embryonic development. Proc. Natl. Acad. Sci. U.S.A. 109 (5): E225–33. 10.1073/pnas.1120033109 22203997 PMC3277139

[133] Hass N, Haub H, Stevens R, Breer H, Schwarzenbacher K (2008) Expression of adiponectin receptor 1 in olfactory mucosa of mice. Cell Tissue Res. 334 (2): 187–197. 10.1007/s00441-008-0677-6 18791742

[134] Garitaonandia I, Smith JL, Kupchak BR, Lyons TJ (2009) Adiponectin identified as an agonist for PAQR3/RKTG using a yeast-based assay system. J. Recept. Signal Transduct. Res. 29 (1): 67–73. 10.1080/10799890902729456 19519172 PMC2792888

[135] Kadowaki T, Yamauchi T (2005) Adiponectin and adiponectin receptors. Endocr. Rev. 26 (3): 439–451. 10.1210/er.2005-0005 15897298

[136] Savigner A, Duchamp-Viret P, Grosmaitre X, Chaput M, Garcia S, et al (2009) Modulation of spontaneous and odorant-evoked activity of rat olfactory sensory neurons by two anorectic peptides, insulin and leptin. J. Neurophysiol. 101 (6): 2898–2906. 10.1152/jn.91169.2008 19297511 PMC2694118

[137] Sreedharan S, Almén MS, Carlini VP, Haitina T, Stephansson O, et al (2011) The G protein coupled receptor Gpr153 shares common evolutionary origin with Gpr162 and is highly expressed in central regions including the thalamus, cerebellum and the arcuate nucleus. FEBS J. 278 (24): 4881–4894. 10.1111/j.1742-4658.2011.08388.x 21981325

[138] Engel, Kathrin M Y, Schröck K, Teupser D, Holdt LM, Tönjes A, et al (2011) Reduced food intake and body weight in mice deficient for the G protein-coupled receptor GPR82. PLoS ONE 6 (12): e29400 10.1371/journal.pone.0029400 22216272 PMC3247265

[139] Kurtenbach S, Mayer C, Pelz T, Hatt H, Leese F, et al (2011) Molecular evolution of a chordate specific family of G protein-coupled receptors. BMC Evol. Biol. 11: 234 10.1186/1471-2148-11-234 21827690 PMC3238225

[140] Kolmakov NN, Kube M, Reinhardt R, Canario AVM (2008) Analysis of the goldfish Carassius auratus olfactory epithelium transcriptome reveals the presence of numerous non-olfactory GPCR and putative receptors for progestin pheromones. BMC Genomics 9: 429 10.1186/1471-2164-9-429 18803863 PMC2556351

[141] Gloriam DE, Fredriksson R, Schiöth HB (2007) The G protein-coupled receptor subset of the rat genome. BMC Genomics 8: 338 10.1186/1471-2164-8-338 17892602 PMC2117022

[142] Bjarnadóttir TK, Gloriam DE, Hellstrand SH, Kristiansson H, Fredriksson R, et al (2006) Comprehensive repertoire and phylogenetic analysis of the G protein-coupled receptors in human and mouse. Genomics 88 (3): 263–273. 10.1016/j.ygeno.2006.04.001 16753280

[143] Jones KA, Borowsky B, Tamm JA, Craig DA, Durkin MM, et al (1998) GABA(B) receptors function as a heteromeric assembly of the subunits GABA(B)R1 and GABA(B)R2. Nature 396 (6712): 674–679. 10.1038/25255 9872315

[144] Priest CA, Puche AC (2004) GABAB receptor expression and function in olfactory receptor neuron axon growth. J. Neurobiol. 60 (2): 154–165. 10.1002/neu.20011 15266647

[145] Olianas MC, Onali P (1999) Mediation by G protein betagamma subunits of the opioid stimulation of adenylyl cyclase activity in rat olfactory bulb. Biochem. Pharmacol. 57 (6): 649–652. 10.1016/S0006-2952(98)00326-8 10037449

[146] Tillerson JL, Caudle WM, Parent JM, Gong C, Schallert T, et al (2006) Olfactory discrimination deficits in mice lacking the dopamine transporter or the D2 dopamine receptor. Behav. Brain Res. 172 (1): 97–105. 10.1016/j.bbr.2006.04.025 16765459

[147] Niedernberg A, Tunaru S, Blaukat A, Ardati A, Kostenis E (2003) Sphingosine 1-phosphate and dioleoylphosphatidic acid are low affinity agonists for the orphan receptor GPR63. Cell. Signal. 15 (4): 435–446. 10.1016/S0898-6568(02)00119-5 12618218

[148] Trifonov S, Houtani T, Kase M, Toida K, Maruyama M, et al (2012) Lateral regions of the rodent striatum reveal elevated glutamate decarboxylase 1 mRNA expression in medium-sized projection neurons. Eur. J. Neurosci. 35 (5): 711–722. 10.1111/j.1460-9568.2012.08001.x 22332935

[149] Tissir F, Qu Y, Montcouquiol M, Zhou L, Komatsu K, et al (2010) Lack of cadherins Celsr2 and Celsr3 impairs ependymal ciliogenesis, leading to fatal hydrocephalus. Nat. Neurosci. 13 (6): 700–707. 10.1038/nn.2555 20473291

[150] Langenhan T, Russ AP (2010) Latrophilin signalling in tissue polarity and morphogenesis. Adv. Exp. Med. Biol. 706: 37–48. 10.1007/978-1-4419-7913-1_3 21618824

[151] Armstrong A, Ryu YK, Chieco D, Kuruvilla R (2011) Frizzled3 is required for neurogenesis and target innervation during sympathetic nervous system development. J. Neurosci. 31 (7): 2371–2381. 10.1523/JNEUROSCI.4243-10.2011 21325504 PMC3046637

[152] Mustata RC, van Loy T, Lefort A, Libert F, Strollo S, et al (2011) Lgr4 is required for Paneth cell differentiation and maintenance of intestinal stem cells ex vivo. EMBO Rep. 12 (6): 558–564. 10.1038/embor.2011.52 21508962 PMC3128273

[153] Yu HI, Jin Y, Fu J, Hsu W (2010) Expression of Gpr177, a Wnt trafficking regulator, in mouse embryogenesis. Dev. Dyn. 239 (7): 2102–2109. 10.1002/dvdy.22336 20549736 PMC2894299

[154] Chang JT, Lowery LA, Sive H (2012) Multiple roles for the Na, K-ATPase subunits, Atp1a1 and Fxyd1, during brain ventricle development. Dev. Biol. 368 (2): 312–322. 10.1016/j.ydbio.2012.05.034 22683378 PMC3402628

[155] Rengaraj D, Lee BR, Park KJ, Lee SI, Kang KS, et al (2011) The distribution of neuron-specific gene family member 1 in brain and germ cells: Implications for the regulation of germ-line development by brain. Dev. Dyn. 240 (4): 850–861. 10.1002/dvdy.22575 21404368

[156] Barros SA, Tennant RW, Cannon RE (2003) Molecular structure and characterization of a novel murine ABC transporter, Abca13. Gene 307: 191–200. 10.1016/S0378-1119(03)00465-7 12706902

[157] Jacobsson JA, Haitina T, Lindblom J, Fredriksson R (2007) Identification of six putative human transporters with structural similarity to the drug transporter SLC22 family. Genomics 90 (5): 595–609. 10.1016/j.ygeno.2007.03.017 17714910

[158] Xiang M, Mohamalawari D, Rao R (2005) A novel isoform of the secretory pathway Ca2+, Mn(2+)-ATPase, hSPCA2, has unusual properties and is expressed in the brain. J. Biol. Chem. 280 (12): 11608–11614. 10.1074/jbc.M413116200 15677451

[159] Radhakrishnan K, Krieger A, Dibué M, Hescheler J, Schneider T (2011) APLP1 and Rab5A interact with the II-III loop of the voltage-gated Ca-channel Ca(v)2.3 and modulate its internalization differently. Cell. Physiol. Biochem. 28 (4): 603–612. 10.1159/000335756 22178872

[160] Palty R, Raveh A, Kaminsky I, Meller R, Reuveny E (2012) SARAF inactivates the store operated calcium entry machinery to prevent excess calcium refilling. Cell 149 (2): 425–438. 10.1016/j.cell.2012.01.055 22464749

[161] Nepravishta R, Polizio F, Paci M, Melino S (2012) A metal-binding site in the RTN1-C protein: new perspectives on the physiological role of a neuronal protein. Metallomics 4 (5): 480–487. 10.1039/c2mt20035j 22522967

[162] Hjelmqvist L, Tuson M, Marfany G, Herrero E, Balcells S, et al (2002) ORMDL proteins are a conserved new family of endoplasmic reticulum membrane proteins. Genome Biol. 3 (6): RESEARCH0027 10.1186/gb-2002-3-6-research0027 12093374 PMC116724

[163] Kim AY, Tang Z, Liu Q, Patel KN, Maag D, et al (2008) Pirt, a phosphoinositide-binding protein, functions as a regulatory subunit of TRPV1. Cell 133 (3): 475–485. 10.1016/j.cell.2008.02.053 18455988 PMC2605970

[164] Billig GM, Pál B, Fidzinski P, Jentsch TJ (2011) Ca2+-activated Cl− currents are dispensable for olfaction. Nat. Neurosci. 14 (6): 763–769. 10.1038/nn.2821 21516098

[165] Pifferi S, Dibattista M, Sagheddu C, Boccaccio A, Al Qteishat A et al (2009) Calcium-activated chloride currents in olfactory sensory neurons from mice lacking bestrophin-2. J. Physiol. (Lond.) 587 (Pt 17): 4265–4279. 10.1113/jphysiol.2009.176131 19622610 PMC2754364

[166] Zhang X, Bertaso F, Yoo JW, Baumgärtel K, Clancy SM, et al (2010) Deletion of the potassium channel Kv12.2 causes hippocampal hyperexcitability and epilepsy. Nat. Neurosci. 13 (9): 1056–1058. 10.1038/nn.2610 20676103 PMC2928878

[167] Hardman RM, Forsythe ID (2009) Ether-à-go-go-related gene K+ channels contribute to threshold excitability of mouse auditory brainstem neurons. J. Physiol. (Lond.) 587 (Pt 11): 2487–2497. 10.1113/jphysiol.2009.170548 19359372 PMC2714015

[168] Hagendorf S, Fluegge D, Engelhardt C, Spehr M (2009) Homeostatic control of sensory output in basal vomeronasal neurons: activity-dependent expression of ether-à-go-go-related gene potassium channels. J. Neurosci. 29 (1): 206–221. 10.1523/JNEUROSCI.3656-08.2009 19129398 PMC6664915

[169] Zou A, Lin Z, Humble M, Creech CD, Wagoner PK, et al (2003) Distribution and functional properties of human KCNH8 (Elk1) potassium channels. Am. J. Physiol., Cell Physiol. 285 (6): C1356–66. 10.1152/ajpcell.00179.2003 12890647

[170] Eldstrom J, Doerksen KW, Steele DF, Fedida D (2002) N-terminal PDZ-binding domain in Kv1 potassium channels. FEBS Lett. 531 (3): 529–537. 10.1016/S0014-5793(02)03572-X 12435606

[171] Talley EM, Solorzano G, Lei Q, Kim D, Bayliss DA (2001) Cns distribution of members of the two-pore-domain (KCNK) potassium channel family. J. Neurosci. 21 (19): 7491–7505. 11567039 10.1523/JNEUROSCI.21-19-07491.2001 PMC6762917

[172] Lesage F, Terrenoire C, Romey G, Lazdunski M (2000) Human TREK2, a 2P domain mechano-sensitive K+ channel with multiple regulations by polyunsaturated fatty acids, lysophospholipids, and Gs, Gi, and Gq protein-coupled receptors. J. Biol. Chem. 275 (37): 28398–28405. 10.1074/jbc.M002822200 10880510

[173] Noël J, Zimmermann K, Busserolles J, Deval E, Alloui A, et al (2009) The mechano-activated K+ channels TRAAK and TREK-1 control both warm and cold perception. EMBO J. 28 (9): 1308–1318. 10.1038/emboj.2009.57 19279663 PMC2683043

[174] Chang K, Pastan I (1996) Molecular cloning of mesothelin, a differentiation antigen present on mesothelium, mesotheliomas, and ovarian cancers. Proc. Natl. Acad. Sci. U.S.A. 93 (1): 136–140. 10.1073/pnas.93.1.136 8552591 PMC40193

[175] Michibata H, Okuno T, Konishi N, Kyono K, Wakimoto K, et al (2009) Human GPM6A is associated with differentiation and neuronal migration of neurons derived from human embryonic stem cells. Stem Cells Dev. 18 (4): 629–639. 10.1089/scd.2008.0215 19298174

[176] Zhao J, Iida A, Ouchi Y, Satoh S, Watanabe S (2008) M6a is expressed in the murine neural retina and regulates neurite extension. Mol. Vis. 14: 1623–1630. 18776950 PMC2529470

[177] Menini A (1995) Cyclic nucleotide-gated channels in visual and olfactory transduction. Biophys. Chem. 55 (3): 185–196. 10.1016/0301-4622(94)00153-B 7542935

[178] Heldens L, Dirks RP, Hensen, Sanne M M, Onnekink C, van Genesen, Siebe T, et al (2010) Co-chaperones are limiting in a depleted chaperone network. Cell. Mol. Life Sci. 67 (23): 4035–4048. 10.1007/s00018-010-0430-7 20556630 PMC2981734

[179] Cintron NS, Toft D (2006) Defining the requirements for Hsp40 and Hsp70 in the Hsp90 chaperone pathway. J. Biol. Chem. 281 (36): 26235–26244. 10.1074/jbc.M605417200 16854979

[180] Neuhaus EM, Mashukova A, Zhang W, Barbour J, Hatt H (2006) A specific heat shock protein enhances the expression of mammalian olfactory receptor proteins. Chem. Senses 31 (5): 445–452. 10.1093/chemse/bjj049 16565291

[181] Geisert EE, Lu L, Freeman-Anderson NE, Templeton JP, Nassr M, et al (2009) Gene expression in the mouse eye: an online resource for genetics using 103 strains of mice. Mol. Vis. 15: 1730–1763. 19727342 PMC2736153

[182] Ango F, Pin JP, Tu JC, Xiao B, Worley PF, et al (2000) Dendritic and axonal targeting of type 5 metabotropic glutamate receptor is regulated by homer1 proteins and neuronal excitation. J. Neurosci. 20 (23): 8710–8716. 11102477 10.1523/JNEUROSCI.20-23-08710.2000 PMC6773061

[183] Ango F, Prézeau L, Muller T, Tu JC, Xiao B, et al (2001) Agonist-independent activation of metabotropic glutamate receptors by the intracellular protein Homer. Nature 411 (6840): 962–965. 10.1038/35082096 11418862

[184] Mast TG, Brann JH, Fadool DA (2010) The TRPC2 channel forms protein-protein interactions with Homer and RTP in the rat vomeronasal organ. BMC Neurosci 11: 61 10.1186/1471-2202-11-61 20492691 PMC2881103

[185] Magalhaes AC, Dunn H, Ferguson, Stephen S G (2012) Regulation of GPCR activity, trafficking and localization by GPCR-interacting proteins. Br. J. Pharmacol. 165 (6): 1717–1736. 10.1111/j.1476-5381.2011.01552.x 21699508 PMC3372825

[186] Nisar SP, Cunningham M, Saxena K, Pope RJ, Kelly E, et al (2012) Arrestin scaffolds NHERF1 to the P2Y12 receptor to regulate receptor internalization. J. Biol. Chem. 287 (29): 24505–24515. 10.1074/jbc.M112.347104 22610101 PMC3397875

[187] Hall RA, Premont RT, Chow CW, Blitzer JT, Pitcher JA, et al (1998) The beta2-adrenergic receptor interacts with the Na+/H+-exchanger regulatory factor to control Na+/H+ exchange. Nature 392 (6676): 626–630. 10.1038/33458 9560162

[188] Cao TT, Deacon HW, Reczek D, Bretscher A, Zastrow M von (1999) A kinase-regulated PDZ-domain interaction controls endocytic sorting of the beta2-adrenergic receptor. Nature 401 (6750): 286–290. 10.1038/45816 10499588

[189] Li J, Chen C, Liu-Chen L (2002) Ezrin-radixin-moesin-binding phosphoprotein-50/Na+/H+ exchanger regulatory factor (EBP50/NHERF) blocks U50,488H-induced down-regulation of the human kappa opioid receptor by enhancing its recycling rate. J. Biol. Chem. 277 (30): 27545–27552. 10.1074/jbc.M200058200 12004055

[190] Hwang JI, Heo K, Shin KJ, Kim E, Yun C, et al (2000) Regulation of phospholipase C-beta 3 activity by Na+/H+ exchanger regulatory factor 2. J. Biol. Chem. 275 (22): 16632–16637. 10.1074/jbc.M001410200 10748023

[191] Wheeler D, Garrido JL, Bisello A, Kim YK, Friedman PA, et al (2008) Regulation of parathyroid hormone type 1 receptor dynamics, traffic, and signaling by the Na+/H+ exchanger regulatory factor-1 in rat osteosarcoma ROS 17/2.8 cells. Mol. Endocrinol. 22 (5): 1163–1170. 10.1210/me.2007-0461 18202147 PMC2366176

[192] Mahon MJ, Donowitz M, Yun CC, Segre GV (2002) Na(+)/H(+) exchanger regulatory factor 2 directs parathyroid hormone 1 receptor signalling. Nature 417 (6891): 858–861. 10.1038/nature00816 12075354

[193] Bassani S, Cingolani LA, Valnegri P, Folci A, Zapata J, et al (2012) The X-linked intellectual disability protein TSPAN7 regulates excitatory synapse development and AMPAR trafficking. Neuron 73 (6): 1143–1158. 10.1016/j.neuron.2012.01.021 22445342 PMC3314997

[194] Cao S, Iyer JK, Lin V (2006) Identification of tetratricopeptide repeat domain 9, a hormonally regulated protein. Biochem. Biophys. Res. Commun. 345 (1): 310–317. 10.1016/j.bbrc.2006.04.091 16678794

[195] Van der Leij I, Franse MM, Elgersma Y, Distel B, Tabak HF (1993) PAS10 is a tetratricopeptide-repeat protein that is essential for the import of most matrix proteins into peroxisomes of Saccharomyces cerevisiae. Proc. Natl. Acad. Sci. U.S.A. 90 (24): 11782–11786. 10.1073/pnas.90.24.11782 8265627 PMC48068

[196] Irmer H, Höhfeld J (1997) Characterization of functional domains of the eukaryotic co-chaperone Hip. J. Biol. Chem. 272 (4): 2230–2235. 10.1074/jbc.272.4.2230 8999928

[197] Sikorski RS, Michaud WA, Wootton JC, Boguski MS, Connelly C, et al (1991) TPR proteins as essential components of the yeast cell cycle. Cold Spring Harb. Symp. Quant. Biol. 56: 663–673. 10.1101/SQB.1991.056.01.075 1819514

[198] Schultz J, Marshall-Carlson L, Carlson M (1990) The N-terminal TPR region is the functional domain of SSN6, a nuclear phosphoprotein of Saccharomyces cerevisiae. Mol. Cell. Biol. 10 (9): 4744–4756. 2201901 10.1128/mcb.10.9.4744-4756.1990 PMC361075

[199] Sugasawa T, Lenzen G, Simon S, Hidaka J, Cahen A, et al (2001) The iodocyanopindolol and SM-11044 binding protein belongs to the TM9SF multispanning membrane protein superfamily. Gene 273 (2): 227–237. 10.1016/S0378-1119(01)00587-X 11595169

[200] Liu L, Srikakulam R, Winkelmann DA (2008) Unc45 activates Hsp90-dependent folding of the myosin motor domain. J. Biol. Chem. 283 (19): 13185–13193. 10.1074/jbc.M800757200 18326487 PMC2442312

[201] Chadli A, Graham JD, Abel MG, Jackson TA, Gordon DF, et al (2006) GCUNC-45 is a novel regulator for the progesterone receptor/hsp90 chaperoning pathway. Mol. Cell. Biol. 26 (5): 1722–1730. 10.1128/MCB.26.5.1722-1730.2006 16478993 PMC1430258

[202] Oort PJ, Warden CH, Baumann TK, Knotts TA, Adams SH (2007) Characterization of Tusc5, an adipocyte gene co-expressed in peripheral neurons. Mol. Cell. Endocrinol. 276 (1–2): 24–35. 10.1016/j.mce.2007.06.005 17689857

