# Supplementary figures and images for: Deep Sequencing of the Murine Olfactory Receptor Neuron Transcriptome
Source: PLoS One. 2015 Jan 15;10(1):e0113170. doi: 10.1371/journal.pone.0113170 (PMC4295871; doi:10.1371/journal.pone.0113170)

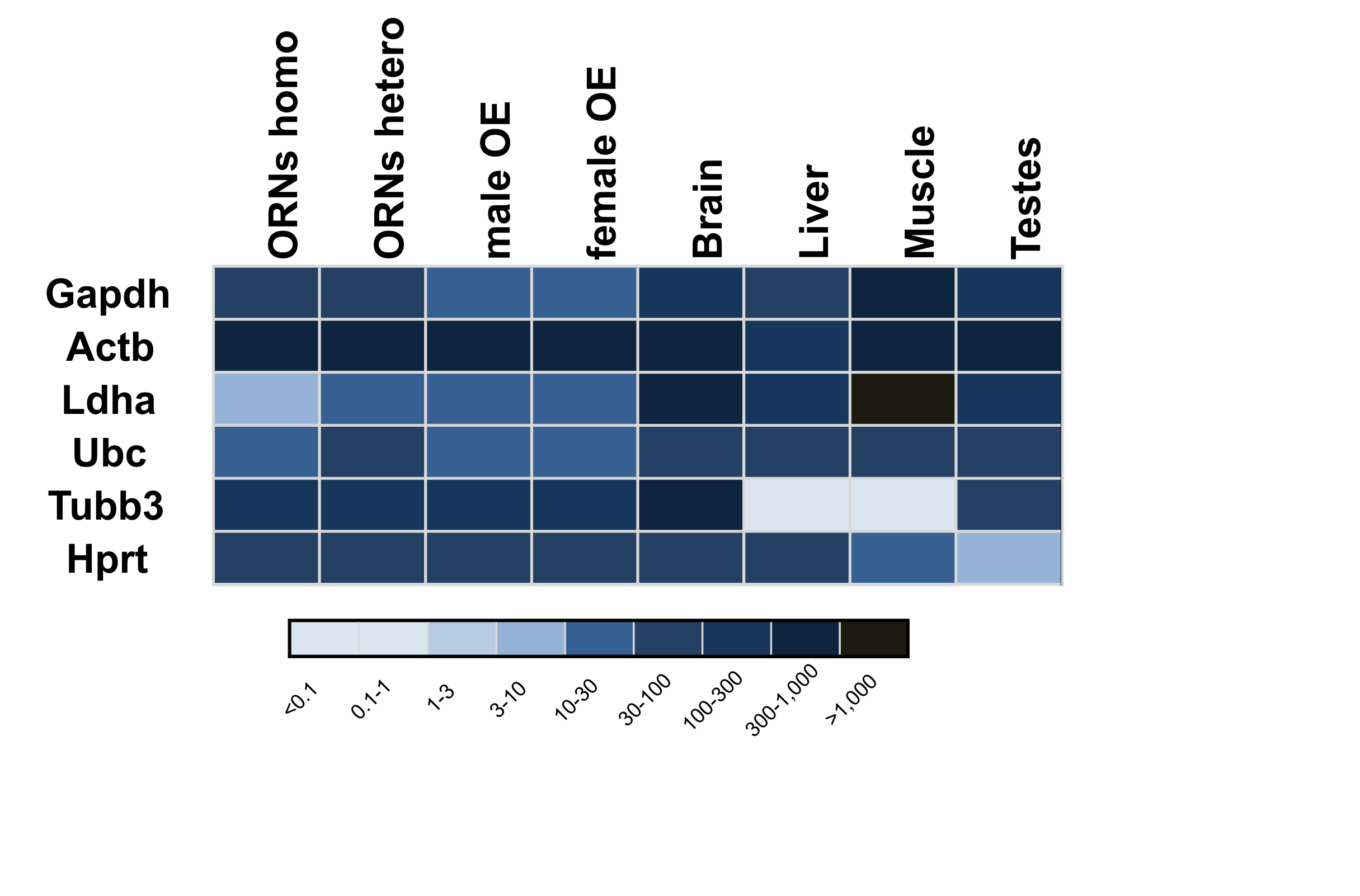

Supplement: S1 Fig — Heatmap showing the expression levels of different housekeeping genes in olfactory and non-olfactory tissue. Higher FPKM values are indicated by deeper colors. Gapdh: glyceraldehyde-3-phosphate dehydrogenase, Actb: actin, cytoplasmic 1, Ldha: L-lactate dehydrogenase A chain isoform 2, Ubc: polyubiquitin-C, Tubb3: tubulin beta-3 chain, Hprt: hypoxanthine-guanine phosphoribosyltransferase. (TIF) [file pone.0113170.s001.tif]

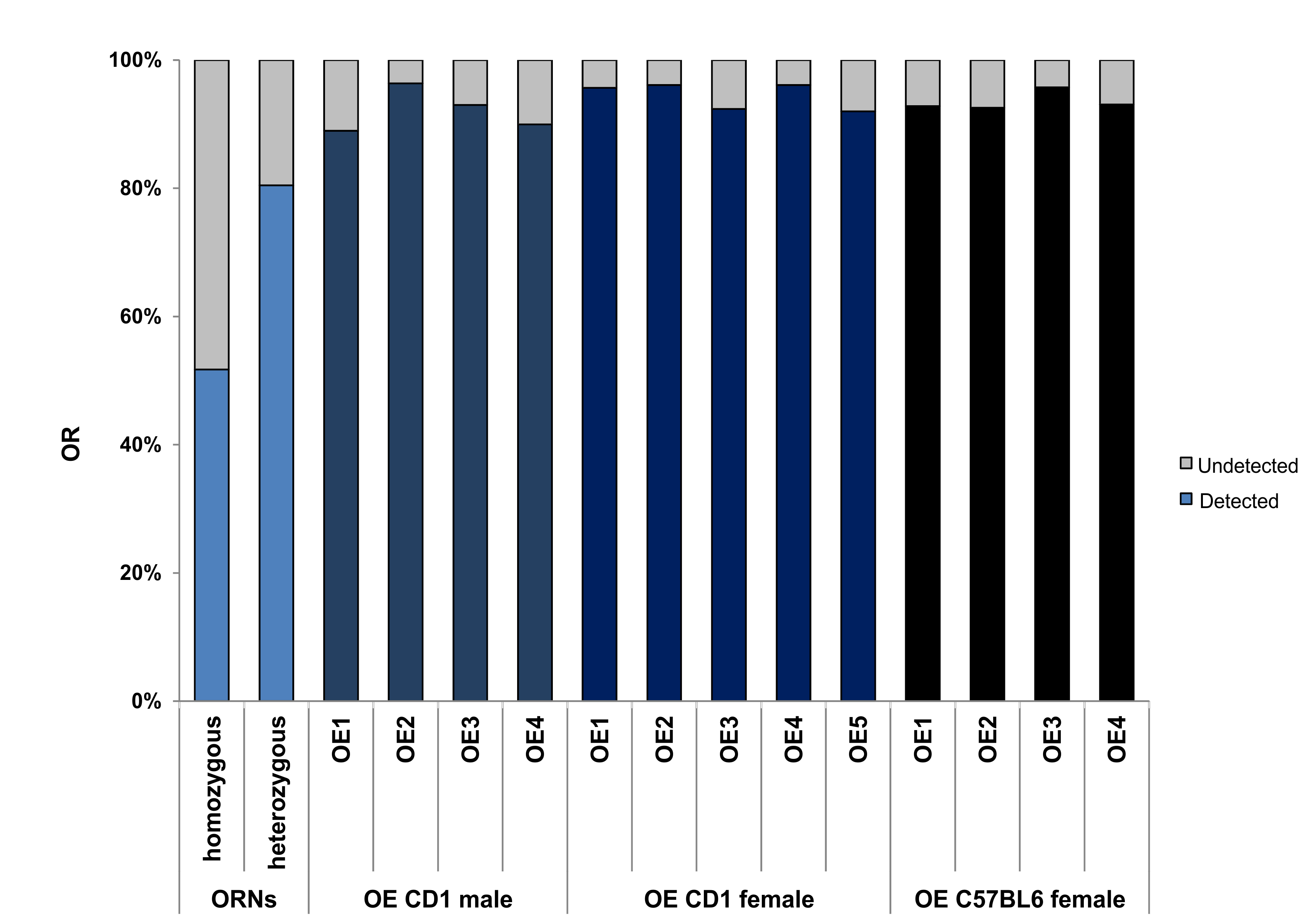

Supplement: S2 Fig — Percentages were calculated based on the 1,125 OR genes annotated in the Refseq based gene model. Bars in light blue: FACS-sorted ORNs (homo- and heterozygous); blue: OE replicates of CD1 male mice (n = 4), dark blue: OE replicates of CD1 female mice (n = 5), black: OE replicates of C57BL6 female mice (n = 4). (TIF) [file pone.0113170.s002.tif]

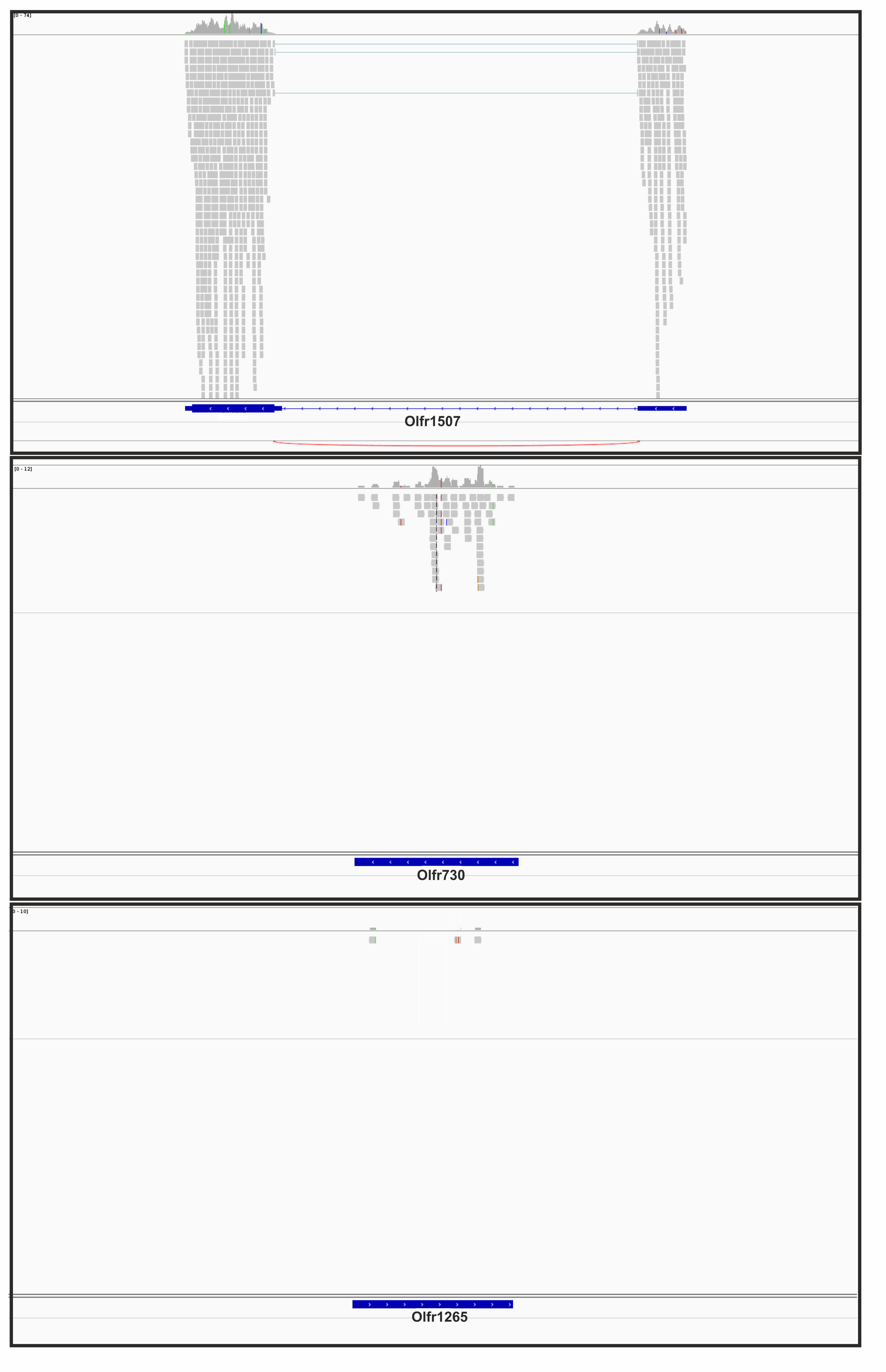

Supplement: S3 Fig — Sample representation of read coverage of ORs with different expression strength can be visualized by the Integrative Genomics Viewer. Shown are exemplary cufflinks data for OE of CD1 female mice; A. Olfr1507, FPKM = 70; B. Olfr730, FPKM = 5.4; C. Olfr1265, FPKM = 0.1. The exons are indicated by blue bars and introns by thin lines. The grey segments indicate reads that were mapped onto reference genome and red bridges exon spanning reads.. Above, the read coverage is shown (detected and mapped counts/bases at each respective position). In highly expressed ORs, 5’ UTRs can be identified by exon-spanning reads. For medium or low expressed ORs, this is not possible due to the lower number of mapped reads. (TIF) [file pone.0113170.s003.tif]

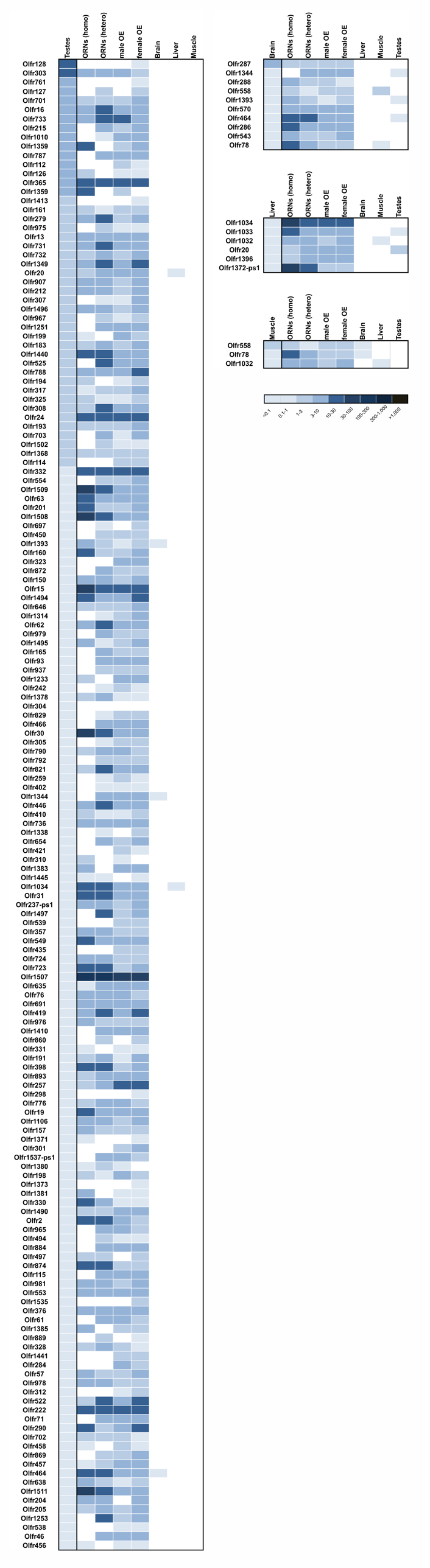

Supplement: S4 Fig — Heatmap showing the expression of OR genes expressed in non-olfactory tissues (testes, brain, liver, muscle). Higher FPKM values are indicated by deeper colors. (TIF) [file pone.0113170.s004.tif]

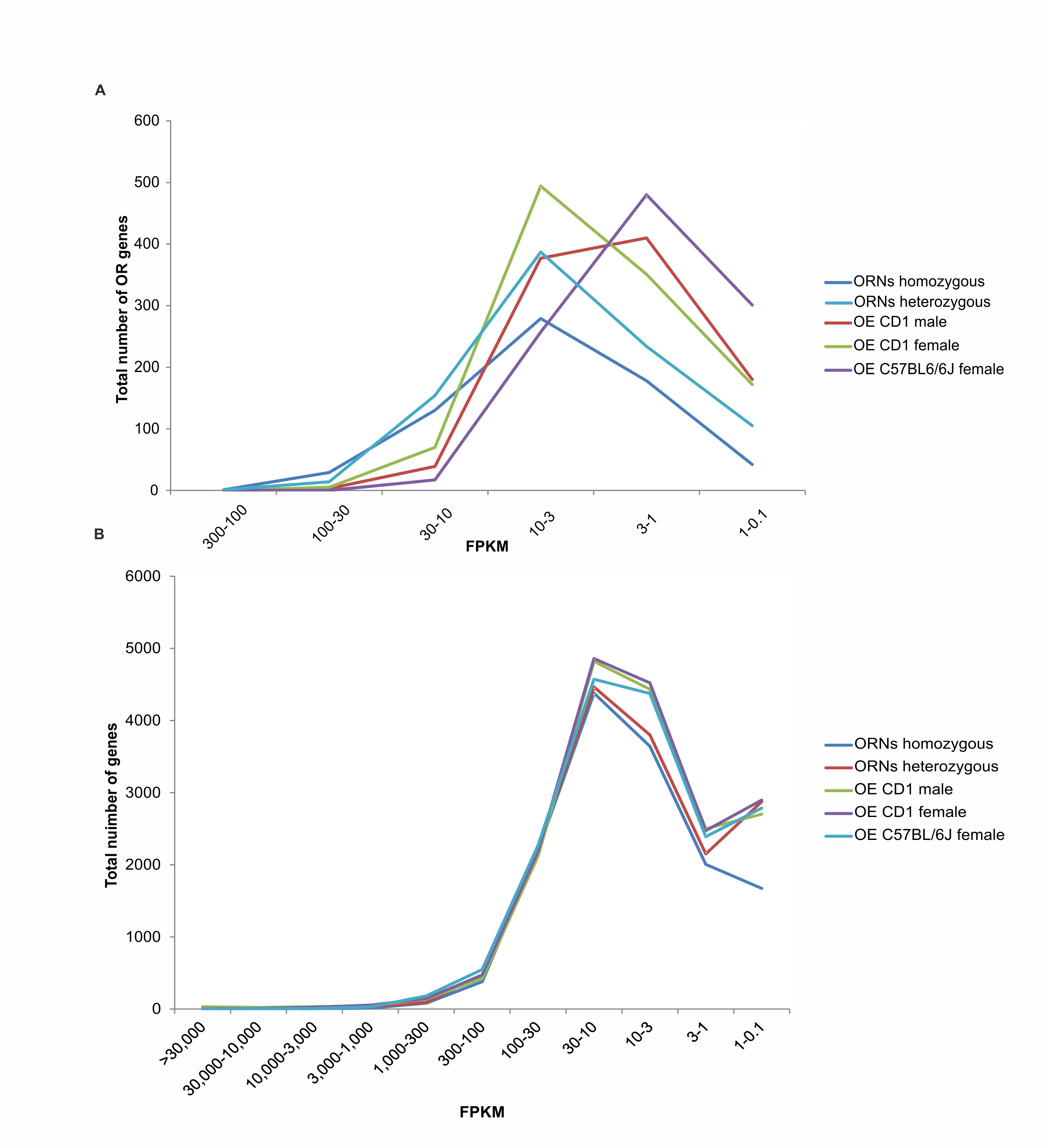

Supplement: S5 Fig — A: Classification of FPKM values of OR genes. Graph showing the distribution of OR genes according to FPKM value classes in olfactory tissues (FACS sorted ORNs, OE CD1 male and female, OE C57BL/6J female. B: Classification of FPKM values of genes. Graph showing the distribution of genes according to FPKM value classes in olfactory tissues. Distribution confirms that the RNA-Seq data comprise the similar number of genes classified into the same range of FPKM values. (TIF) [file pone.0113170.s005.tif]

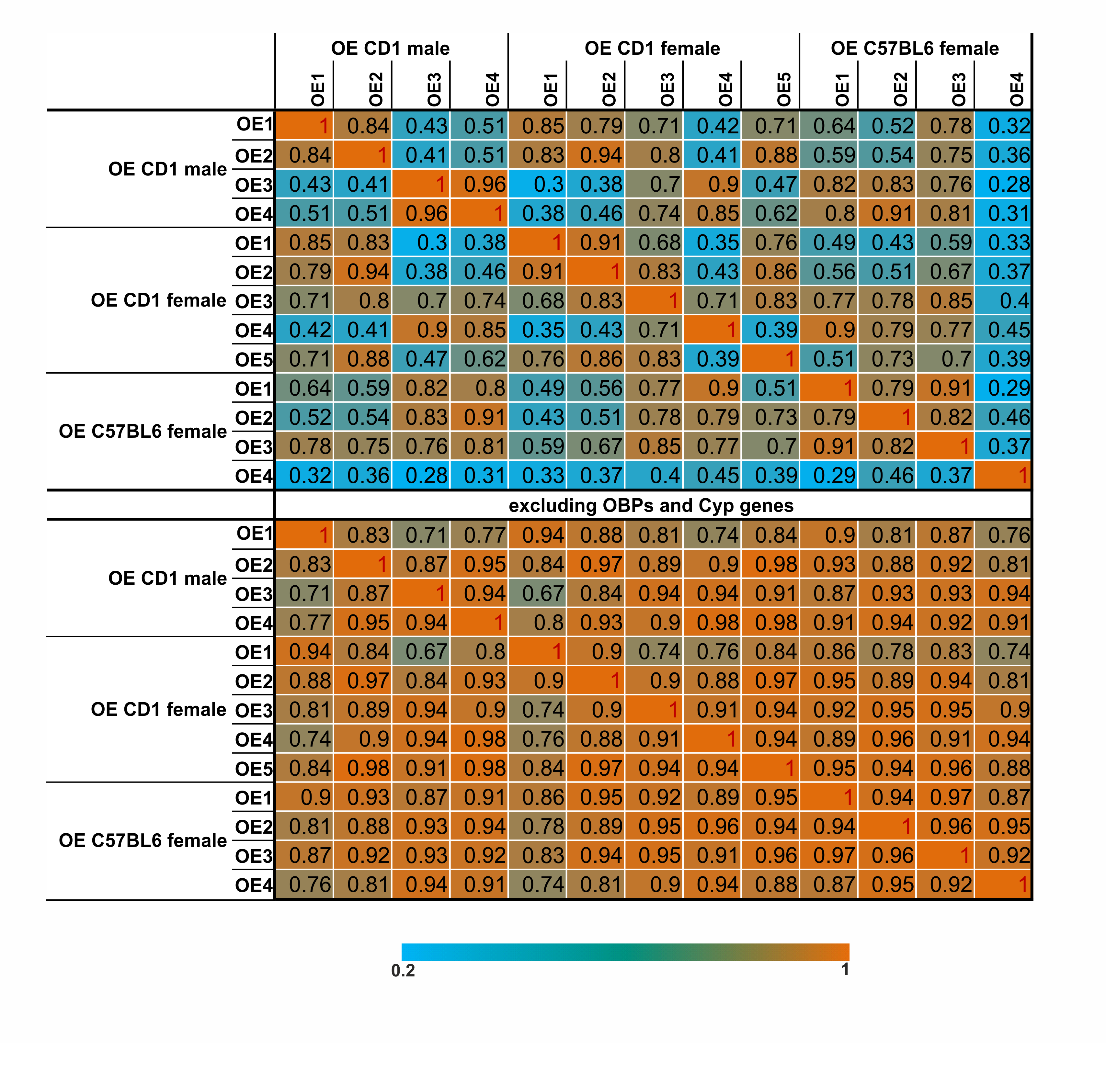

Supplement: S6 Fig — Chart showing the Pearson correlation coefficient values for protein-coding gene expression pattern between all replicates of the OE (upper matrix). In the lower matrix, OBP and Cpy genes were excluded from the analysis. Higher correlation between replicates is indicated by a color scale from blue to orange. (TIF) [file pone.0113170.s006.tif]

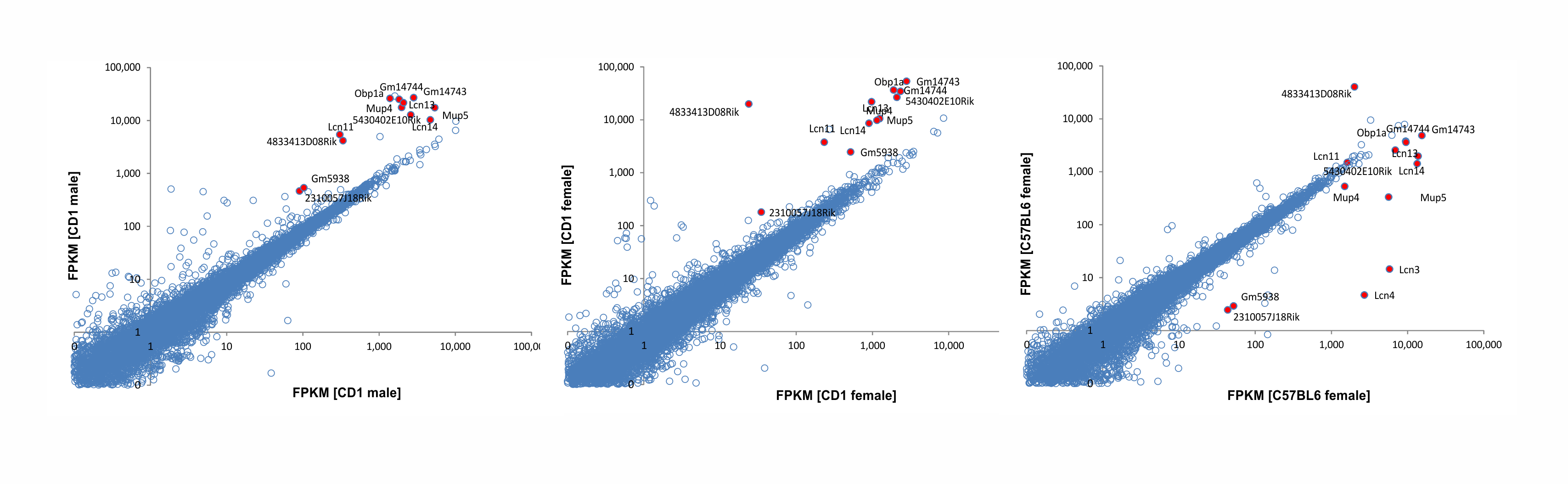

Supplement: S7 Fig — Shown is the correlation of the protein-coding gene expression pattern between OE of two (exemplary chosen) individual mice for each condition (CD1 male, CD1 female and C57BL6 female). Only genes with detectable expression levels (FPKM>0.1) are shown. The FPKM values are logarithmically presented. Genes with the most diverging expression pattern belong to OBPs genes; which are marked in red. (TIF) [file pone.0113170.s007.tif]

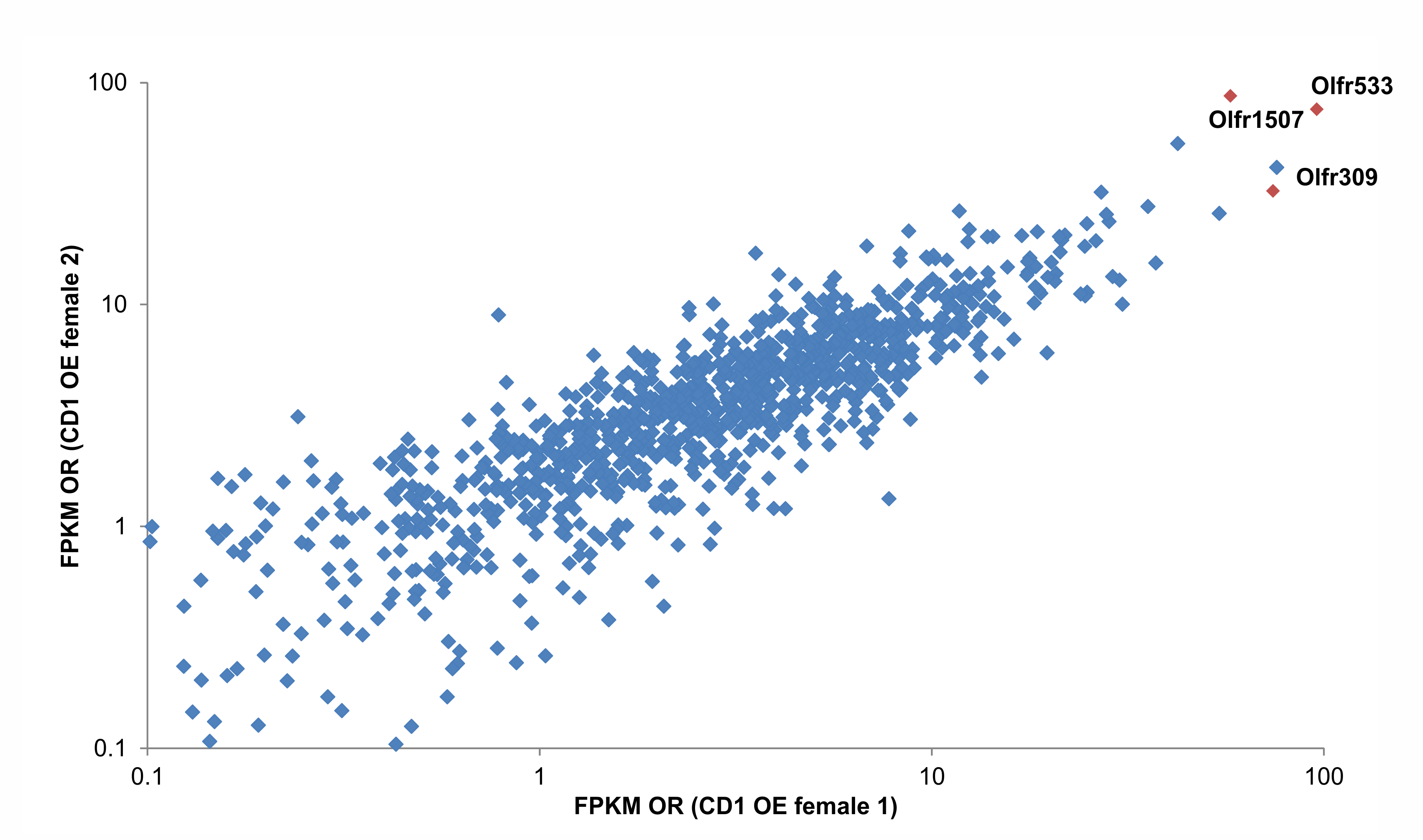

Supplement: S8 Fig — For investigation of the reproducibility of the expression pattern, two biological replicates of the transcriptome of the female OE of CD1 mice were prepared. These new datasets were based on RNA of 8 pooled OE analyzed by mRNA Illumina sequencing on a HiSeq 2000 platform which generated 54–57 million reads (101 bp, paired end). Correlation of the OR gene expression between two biological replicates of female CD1 mice is shown. A detailed analysis of these data will be given elsewhere. Only OR genes with detectable expression levels (FPKM>0.1) are shown. The FPKM values are logarithmically presented. The Pearson correlation coefficient of r = 0.9 confirmed the strong correlation of OR gene expression patterns between biological replicates. (TIF) [file pone.0113170.s008.tif]

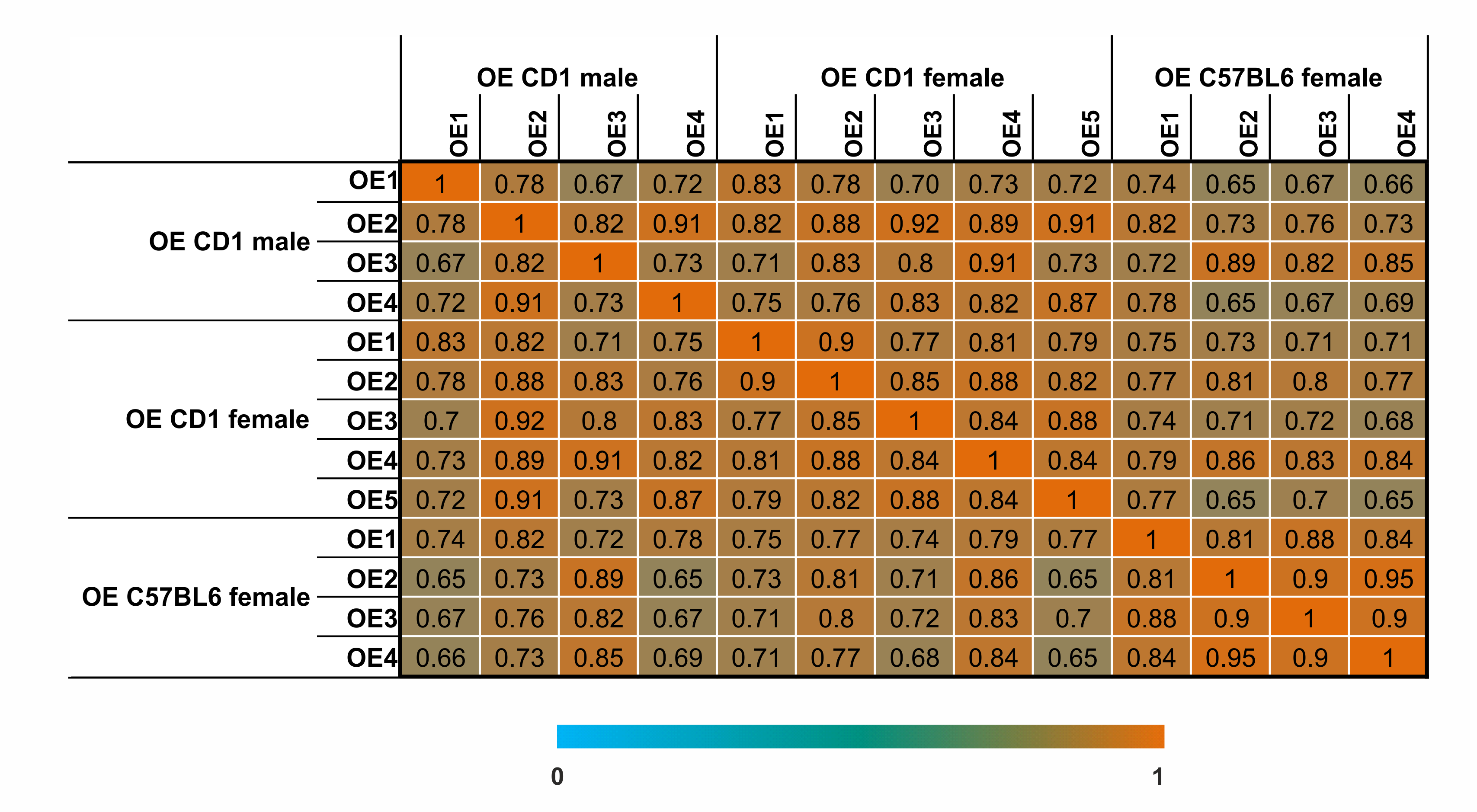

Supplement: S9 Fig — Chart showing the Pearson correlation coefficient values for OR gene expression between all replicates of the OE (n = 13). Higher correlation between replicates is indicated by a color scale from blue to orange. (TIF) [file pone.0113170.s009.tif]

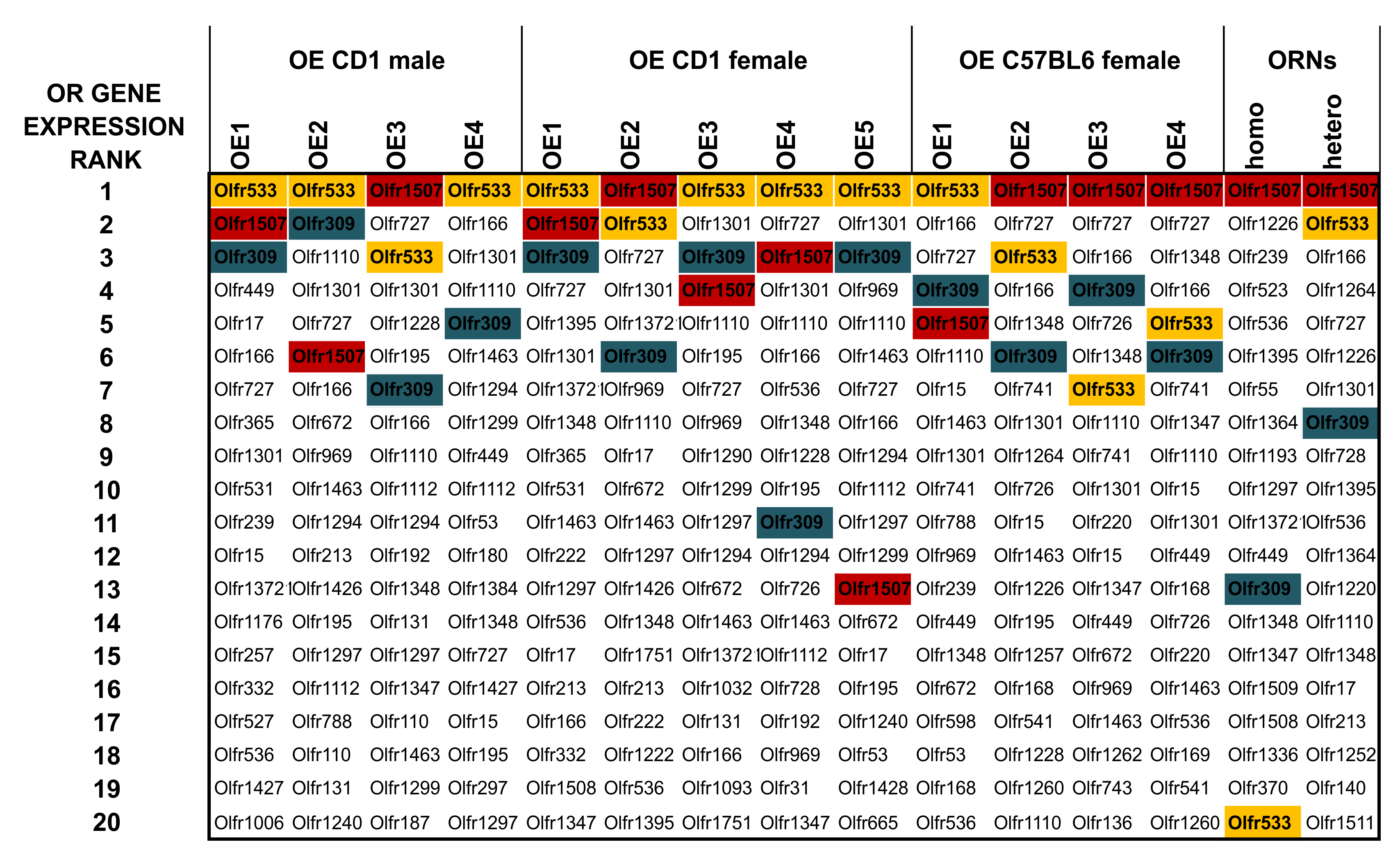

Supplement: S10 Fig — Chart showing the expression ranking of the top 20 OR genes in OE replicates (CD1 male, CD1 female and C57BL6 mice) and ORNs. The OR genes Olfr1507, Olfr533 and Olfr309 are highly expressed. These receptors can be detected among the 20 most highly expressed OR genes in each replicate of the OE and ORNs. (TIF) [file pone.0113170.s010.tif]

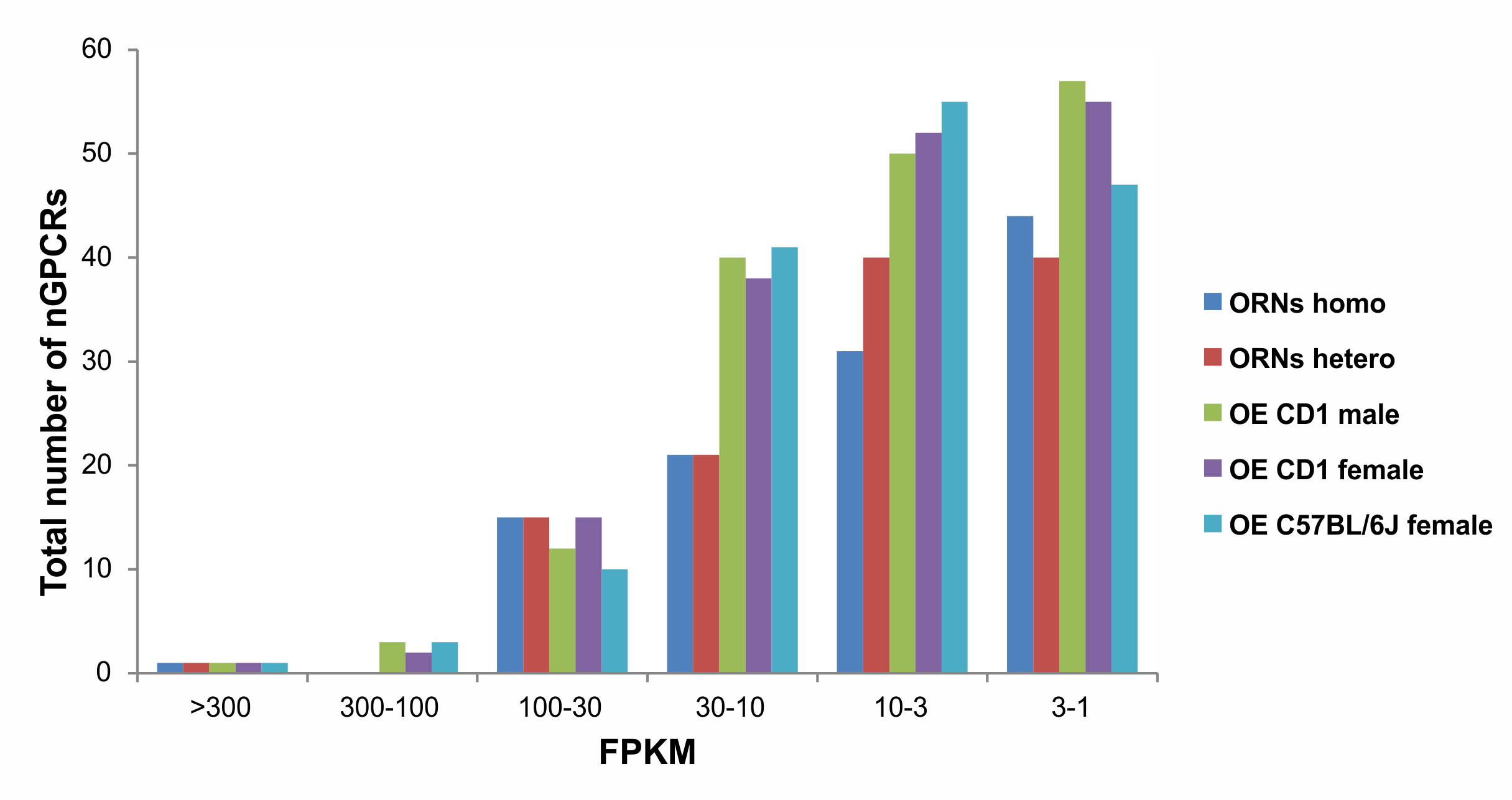

Supplement: S11 Fig — Bar chart showing the distribution of FPKM classes in ORNs, OE of CD1 mice (both sexes) and OE of female C57BL/6J. (TIF) [file pone.0113170.s011.tif]

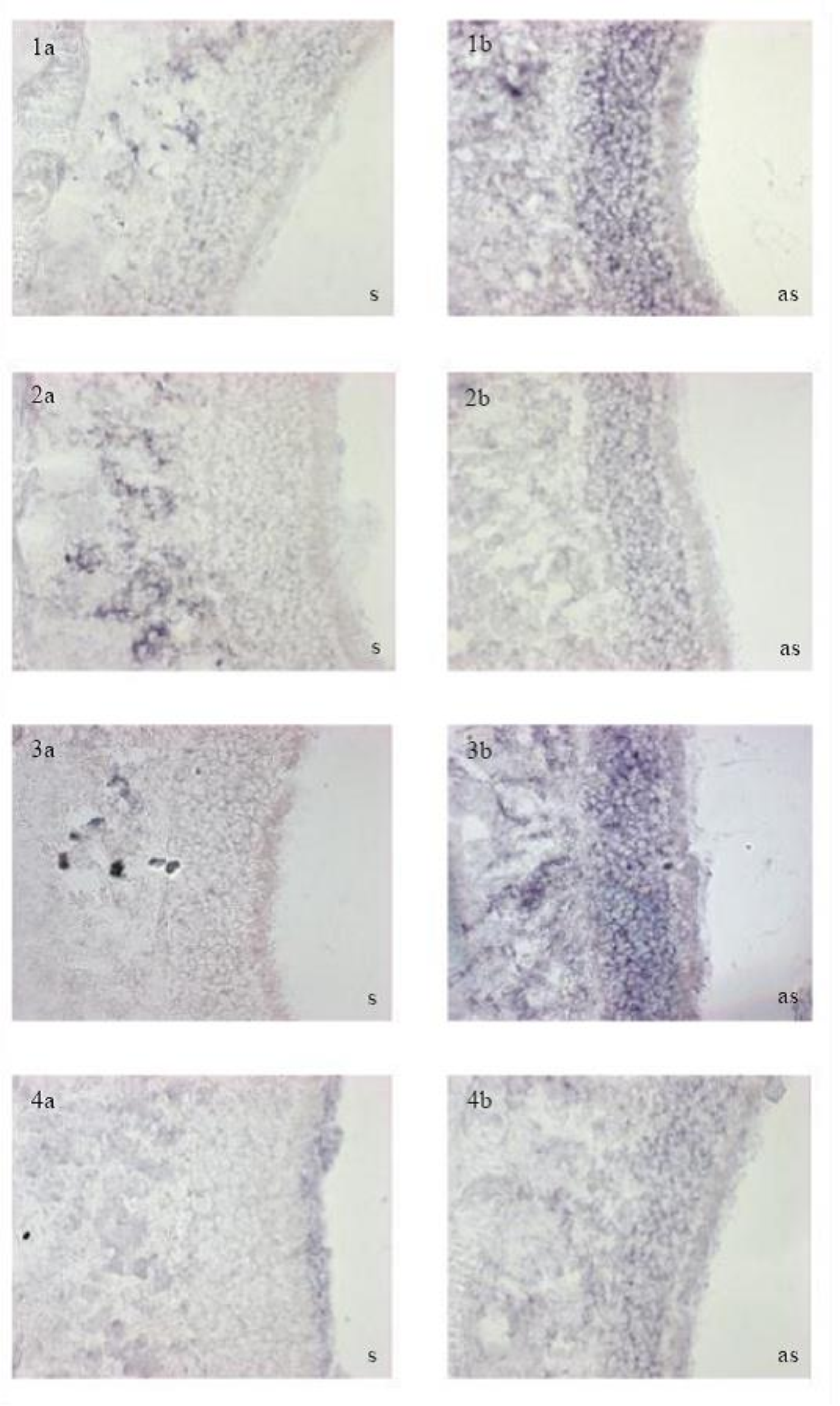

Supplement: S12 Fig — Expression of transcripts for p110α (1), p110β (2), p110γ (3) and p110δ (4) were detected in the mature ORN cell layer as predicted by the expression in sorted ORNs RNA-Seq data. Sense (a) and antisense (b) RNA probes were tested in parallel and show the antisense specific staining (TIF) [file pone.0113170.s012.tif]

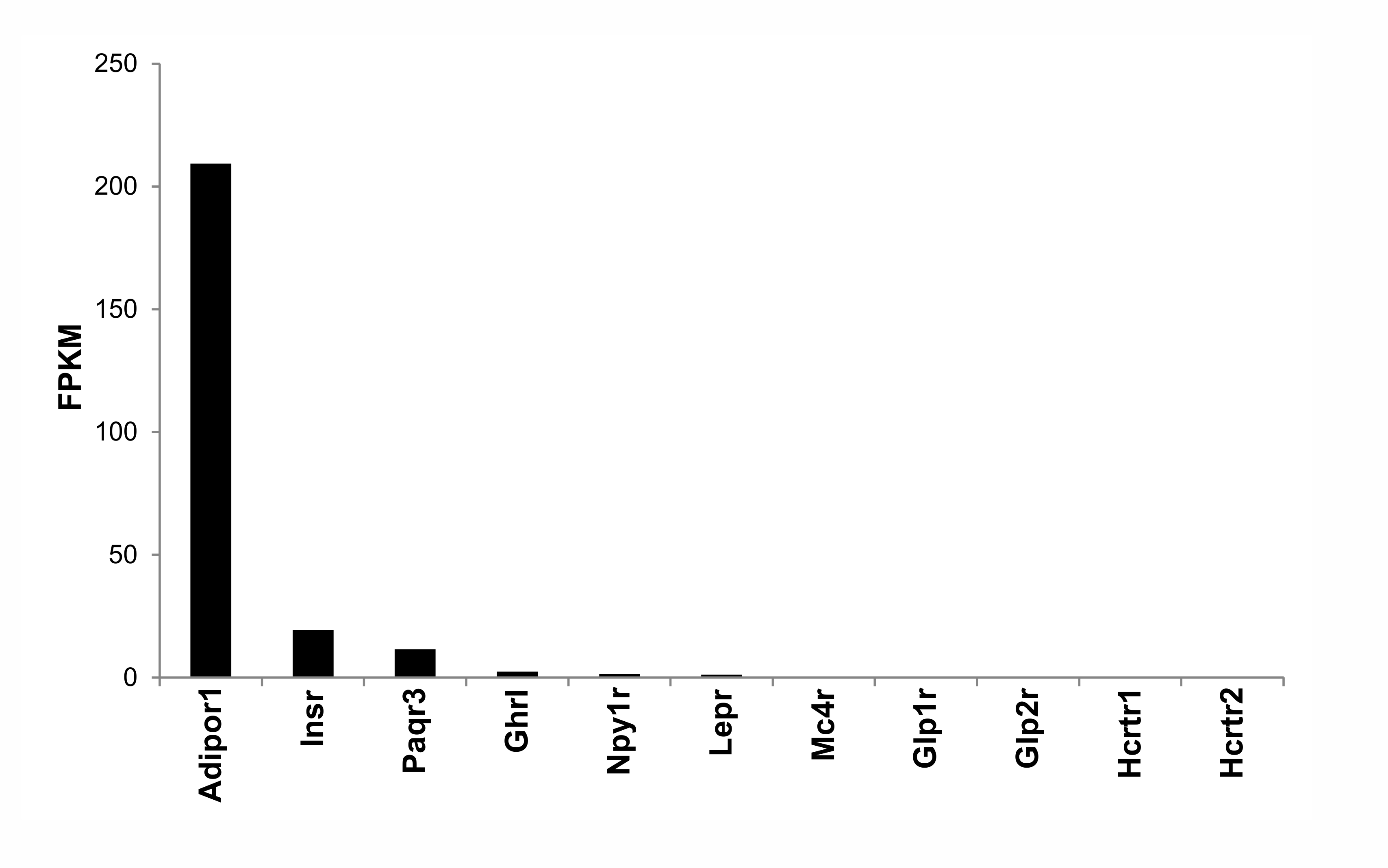

Supplement: S13 Fig — Bar chart showing the expression level of receptor genes regulating food intake in the OE. Adipor1 is by far the most highly expressed gene. Receptors for insulin, leptin or ghrelin, are weakly expressed. FPKM values are presented exemplary from CD1 male OE. Adipor1: adiponectin receptor1, Insr: insulin receptor, Paqr3: progestin and adipoQ receptor family member III, Ghrl: ghrelin receptor, Lepr: leptin receptor, Npy1r: neuropeptide Y receptor, Glp1r/Glp2r: Glucagon-like peptide receptor, Hctr1/Hctr2: orexin receptors, Mc3r/Mc4r: melanocortin receptors. (TIF) [file pone.0113170.s013.tif]
