# Supplementary material for: Deep Sequencing of the Murine Olfactory Receptor Neuron Transcriptome
Source: PLoS One. 2015 Jan 15;10(1):e0113170. doi: 10.1371/journal.pone.0113170 (PMC4295871; doi:10.1371/journal.pone.0113170)
Supplement: S4 Table — (DOCX) [file pone.0113170.s017.docx]

| **Supplementary Table 2a.** Raw data sets of RNASeq used in this study | | | | |
| --- | --- | --- | --- | --- |
| **Tissue** | **Source** | **Accession Number** | **Technology** | **Reference** |
| **Brain** | Sequence Read Archieve | SRR006488 | Illumina/Solexa | Mortazavi et al., 2008 |
|  | Sequence Read Archieve | SRR006489 | 25-bp reads for poly(A)-selected RNA |  |
| **Muscle** | Sequence Read Archieve | SRR001361 | Illumina/Solexa | Mortazavi et al., 2008 |
|  | Sequence Read Archieve | SRR001362 | 25-bp reads for poly(A)-selected RNA |  |
|  | Sequence Read Archieve | SRR006492 |  |  |
| **Liver** | Sequence Read Archieve | SRR006490 | Illumina/Solexa | Mortazavi et al., 2008 |
|  | Sequence Read Archieve | SRR006491 | 25-bp reads for poly(A)-selected RNA |  |
|  | Sequence Read Archieve | SRR001360 |  |  |
|  | Sequence Read Archieve | SRR001359 |  |  |
| **Testes** | Sequence Read Archieve | SRR037136 | Illumina/Genome Analyzer II | Harr and Turner, 2010 |
|  | Sequence Read Archieve | SRR037137 | 46-bp reads from mRNA |  |
|  | Sequence Read Archieve | SRR037138 |  |  |
